# Identification of a physiologic vasculogenic fibroblast state to achieve tissue repair

**DOI:** 10.1038/s41467-023-36665-z

**Published:** 2023-02-28

**Authors:** Durba Pal, Subhadip Ghatak, Kanhaiya Singh, Ahmed Safwat Abouhashem, Manishekhar Kumar, Mohamed S El Masry, Sujit K. Mohanty, Ravichand Palakurti, Yashika Rustagi, Saba Tabasum, Dolly K. Khona, Savita Khanna, Sedat Kacar, Rajneesh Srivastava, Pramod Bhasme, Sumit S. Verma, Edward Hernandez, Anu Sharma, Diamond Reese, Priyanka Verma, Nandini Ghosh, Mahadeo Gorain, Jun Wan, Sheng Liu, Yunlong Liu, Natalia Higuita Castro, Surya C. Gnyawali, William Lawrence, Jordan Moore, Daniel Gallego Perez, Sashwati Roy, Mervin C. Yoder, Chandan K. Sen

**Affiliations:** 1 Indiana Center for Regenerative Medicine & Engineering, Department of Surgery, Indiana University School of Medicine, Indianapolis, IN 46202 USA; 2 Department of Surgery, The Ohio State University, Columbus, OH 43210 USA; 3 Department of Biomedical Engineering, Indian Institute of Technology Ropar, Rupnagar, Punjab 140001 India; 4 Center for Computational Biology and Bioinformatics (CCBB), Indiana University School of Medicine, Indianapolis, IN 46202 USA; 5 Department of Biomedical Engineering, The Ohio State University, Columbus, OH 43210 USA

**Keywords:** miRNAs, Bioinformatics, Gene delivery

## Abstract

Tissue injury to skin diminishes miR-200b in dermal fibroblasts. Fibroblasts are widely reported to directly reprogram into endothelial-like cells and we hypothesized that miR-200b inhibition may cause such changes. We transfected human dermal fibroblasts with anti-miR-200b oligonucleotide, then using single cell RNA sequencing, identified emergence of a vasculogenic subset with a distinct fibroblast transcriptome and demonstrated blood vessel forming function in vivo. Anti-miR-200b delivery to murine injury sites likewise enhanced tissue perfusion, wound closure, and vasculogenic fibroblast contribution to perfused vessels in a FLI1 dependent manner. Vasculogenic fibroblast subset emergence was blunted in delayed healing wounds of diabetic animals but, topical tissue nanotransfection of a single anti-miR-200b oligonucleotide was sufficient to restore FLI1 expression, vasculogenic fibroblast emergence, tissue perfusion, and wound healing. Augmenting a physiologic tissue injury adaptive response mechanism that produces a vasculogenic fibroblast state change opens new avenues for therapeutic tissue vascularization of ischemic wounds.

## Abstract (web summary)

Here, the authors report on the discovery of physiological vasculogenic fibroblasts capable of forming functional blood vessels. In vivo tissue reprogramming triggered by topical tissue nanotransfection (TNT) of a single anti-miR-200b oligonucleotide achieved therapeutic tissue vascularization.

## Introduction

Single cell RNA sequencing has propelled advances in understanding basal and activated fibroblast subset heterogeneity that varies with age^1^, disease^2,3^, injury^4^, and organs^5,6^. Certain myocardial and skeletal muscle fibroblast subsets become activated following ischemic injury to serve as pro-angiogenic fibroblasts that enhance tissue perfusion and reparative processes^3,7^. However, it is unclear if pro-angiogenic fibroblast subsets contribute to new blood vessel formation. Here, we report on the maiden identification of a physiological and inducible vasculogenic fibroblast which serves a critical role in the initiation of functional tissue vascularization.

## Results

### Inhibition of miR-200b in cultured dermal fibroblasts

**Fig. 1:**
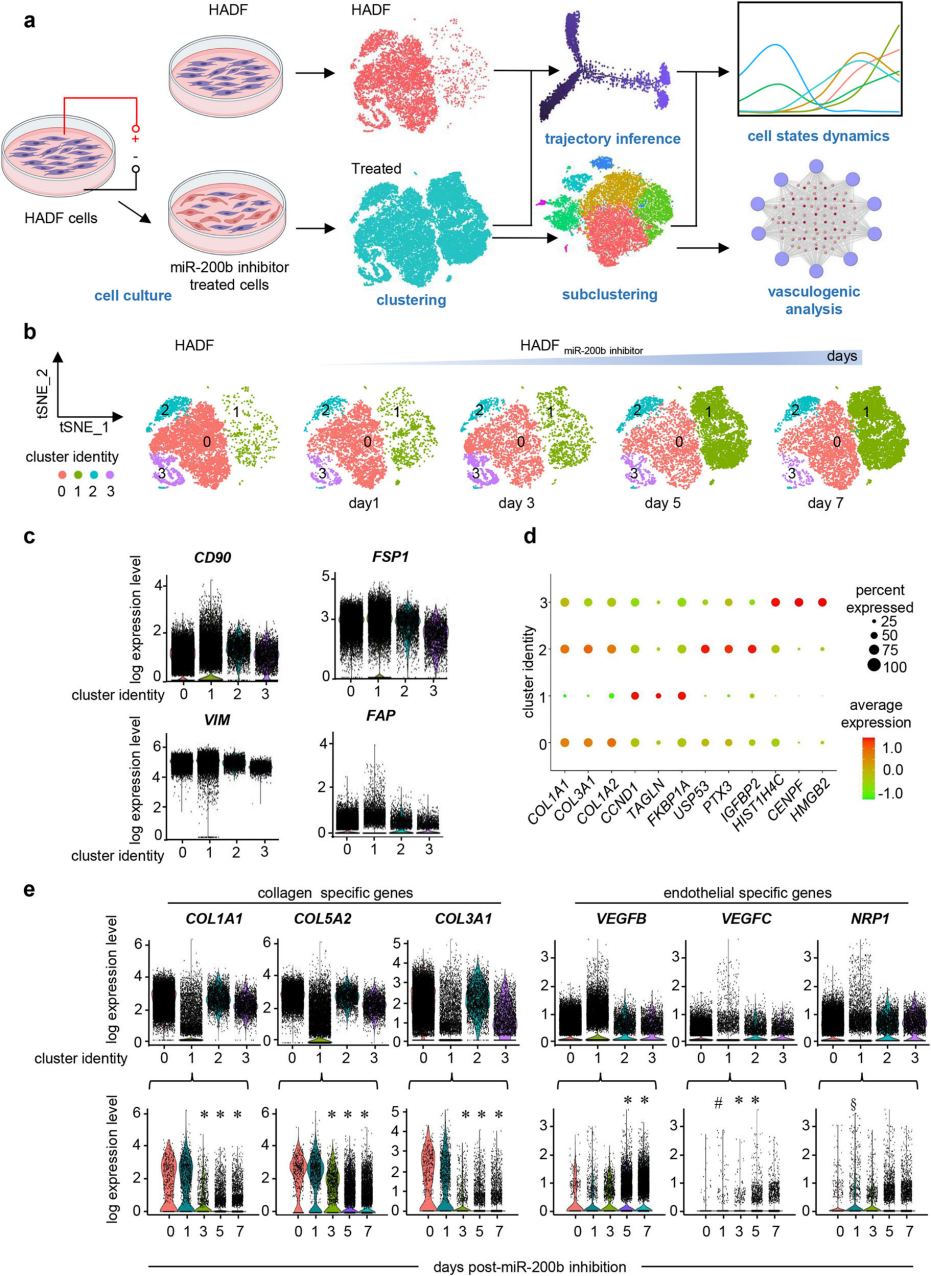
Single-cell RNA sequencing analysis reveals temporal gain of a vasculogenic cluster post miR-200b inhibition. **a** Schematic diagram of nanoelectroporation delivery of miR-200b inhibitor in human adult dermal fibroblasts (HADF) and workflow for obtaining and analyzing scRNA-seq data from fibroblasts (HADF cells). **b** t-SNE plots for HADF cells at different time interval (days 1, 3, 5, and 7) post anti-miR-200b transfection with cells colored according to 4 main clusters. The initial dataset contained 40,212 cells out of which 36,308 cells met quality control measures and were chosen for further downstream analysis. Unsupervised clustering identified 4 cell clusters (clusters 0–3). See Supplementary Fig. 2 c and d for control inhibitor treated HADF cells. **c** Violin plots expression of fibroblast markers (CD90, FSP1, Vimentin, Fibroblast activation protein alpha 1) in different clusters of fibroblasts. Cluster 1 progressively lost characteristic fibroblast gene expression. **d** Dot plot of representative genes for identification of the four cellular clusters in HADF cells post-miR-200b inhibition. **e** Violin plots showing lower expression of collagen genes (COL1A1, COL5A2, COL3A1) and higher expression of VEGF family genes (VEGFB, VEGFC, NRP1) in cluster 1 (upper panel) and their expression within cluster 1 cells over time post anti-miR-200b transfection (lower panel). *P < 0.001; ^#^*P* < 0.048; ^§^*P* < 0.005. Data in **e** was analyzed by one-way analysis of variance with the *post-hoc* Bonferroni multiple comparison test. Fig 1a created with BioRender.com.

**Fig. 2:**
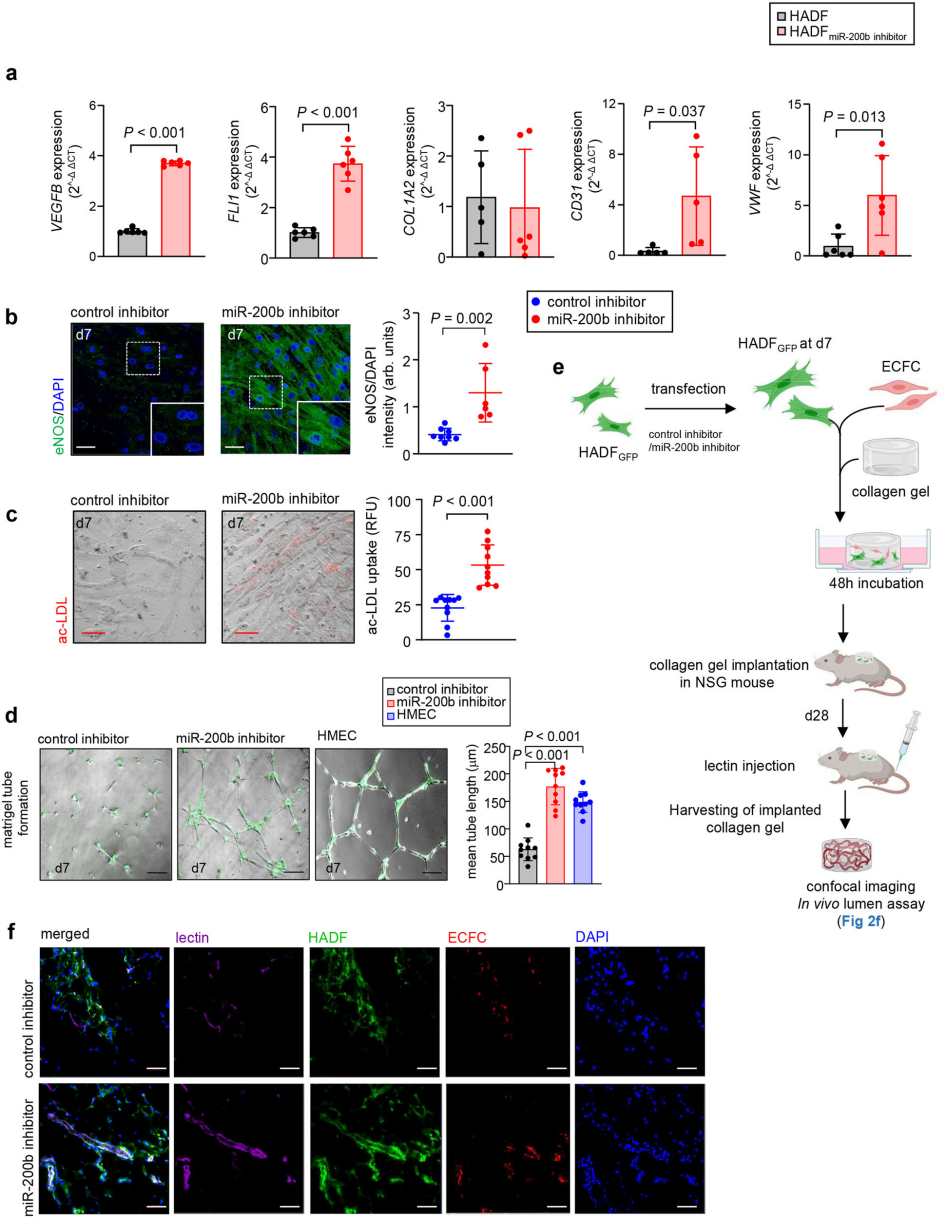
Anti-sense oligonucleotide inhibition of miR-200b in dermal fibroblasts induces vasculogenic state. **a** Quantitative gene expression of key representative transcripts from the cDNA prepared and used for single cell RNA sequencing at day 7 post-miR-200b inhibition. Data are mean ± S.D (*n* = 5–6). **b** Immunofluorescence of eNOS expression in HADF transfected with control or miR-200b inhibitor at d7 post in vitro TNT. Scale, 200 µm. Data are mean ± S.D (*n* = 6–8). **c** Immunocytochemistry of acLDL uptake in HADF transfected with control or miR-200b inhibitor at d7 post in vitro TNT. Scale, 200 µm. Data are mean ± S.D (*n* = 8–10). **d** Representative images showing in vitro Matrigel tube formation by vasculogenic fibroblasts and its analysis. HMEC were used as positive control. Scale, 200 µm. Results represent mean ± S.D (*n* = 10). **e** Schematic diagram showing the experimental design for in vivo collagen gel assay. **f** Immunofluorescence confocal image of in vivo collagen gel assay showing lectin perfused vessels of HADF origin treated with miR 200b inhibitor at day 28. Such perfused vessels were absent in control groups where HADF were treated with only control inhibitor. Scale, 50 µm. Data in **a**–**c** were analyzed by two-tailed unpaired Student’s *t* test. Data in **d** was analyzed by one-way analysis of variance with the *post-hoc* Bonferroni multiple comparison test. Fig 2e created with BioRender.com.

MiR-200b abundance is sharply lowered at the wound-edge of patient skin^8^ (Supplementary Fig. 1a). Murine skin wounding induced a transient 7-day deficit of miR-200b in dermal fibroblast rich tissue (Supplementary Fig. 1b) but, not in K14 + epithelial or F4/80+ macrophage elements (Supplementary Fig. 1c). To understand the significance of lowered miR-200b abundance in fibroblasts, anti-miR-200b oligonucleotide was delivered in vitro to human adult dermal fibroblasts (HADF) (Fig. 1a) via in vitro TNT^9^ with high efficiency (86.21 ± 4.05%; *n* = 5). To interrogate single cell HADF transcriptome complexity, all cells from anti-miR-200b treated samples (days 1, 3, 5, and 7) were compared to pre-treated reference control HADF cells (day 0) (Fig. 1b). The initial dataset contained 40,212 cells and the 36,308 cells that met quality control parameters underwent downstream analysis. Unsupervised clustering identified 4 clusters (Fig. 1b). Compositional analysis revealed cluster 1 cells increased > 10-fold in frequency over 7 days post-transfection but clusters 0, 2, and 3 decreased over time (Fig. 1b). Differentially expressed genes significantly increased over time (Supplementary Fig. 1d) indicative of an activated transcriptionally responsive process^10^. Reactome pathway enrichment for the differentially expressed genes revealed that collagen formation/degradation pathways were among several pathways significantly downregulated over time (Supplementary Fig. 1e, f). However, all 4 clusters displayed well-known fibroblast marker gene transcripts^11–13^ such as CD90^14^, FSP1 (S100A4)^15^, vimentin^16^, and fibroblast activation protein alpha 1 (FAP)^17^ (Fig. 1c). Among the 4 clusters, cluster 1 uniquely and temporally lost collagen gene transcripts (Fig. 1d, e), while significantly gaining endothelial gene transcripts (Fig. 1e, Supplementary Fig. 2a, b), including numerous VEGF pathway genes that play important roles in angiogenesis and vasculogenesis^14,18,19^. Quantitative validation of endothelial gene transcripts in cluster 1, was confirmed without Col1A1 transcript abundance change (Fig. 2a). As an additional control, HADF cells were treated with a control or anti-miR-200b oligonucleotide by TNT. A significant difference in endothelial transcripts at 7 days post-transfection was detected for all clusters combined and for cluster 1, clarifying that the induced gain in endothelial transcripts was not related to transfection of any oligonucleotide into HADF (Supplementary Fig. 2c, d). Analysis of cell surface proteins in clusters 0-4 (Supplementary Fig. 3a) identified cluster 1 as beta 2 microglobulin high (B2M^hi^) and integrin alpha 2 low (ITGA2^lo^) while all remaining clusters were B2M^lo^ and ITGA2^hi^. Flow cytometric sorting of these subsets identified the B2M^hi^ITGA2^lo^ cells displaying the lowest miR-200b abundance and highest FLI1 expression among the anti-miR-200b post-transfected day 3 HADF (Supplementary Fig. 3b, c). No significant differences were observed in cell cycle genes when compared to cluster 0 and 1 at 1- and 7-days post-transfection (Supplementary Fig. 3e). Akin to the function of endothelial cells, cluster 1 cells also displayed endothelial nitric oxide synthase (eNOS) expression (Fig. 2b), ingested acetylated-low density lipoprotein (ac-LDL) (Fig. 2c), and formed branching tubular structures when plated on Matrigel (Fig. 2d). These results reflected acquisition of angiogenic properties in treated fibroblasts but did not formally prove development of lumenized vessels de novo from endothelial precursors^20^. Thus, commercially available HADF expressing green fluorescent protein (GFP) were treated with anti-miR-200b or control oligonucleotides and suspended along with tdTomato+ cord blood endothelial colony forming cells (ECFC) (1:1 ratio with ECFC as positive control) in collagen gels, cultured in vitro for 48 h, and implanted within the subcutaneous space of immunodeficient mice. Four weeks later, confirmation of intravenously delivered lectin perfused chimeric capillary vessels comprised of human vasculogenic fibroblasts (VF) and some tdTomato+ ECFC cells were detected in 6 of 9 test mice (Fig. 2e, f). No HADF-GFP control cells formed vessels (*n* = 6). Thus, cultured HADF, treated with TNT delivered anti-miR-200b oligonucleotides displayed acquisition of the capacity to form perfused human blood vessels for at least one month in immunodeficient mice. However, these COL1A2 + VEGFR2 + VF continued to remodel and contract 3D collagen gels as effectively as control HADF cells in contrast to equal numbers of HMEC control cells that lacked gel contraction capacity (χ2 = 61; *p* =< 0.0001; *n* = 37). Thus, some HADF attained vasculogenic capacity and yet retained functional features of a fibroblast cell upon TNT mediated anti-miR-200b delivery.

### HADF enters new states upon miR-200b inhibition

**Fig. 3:**
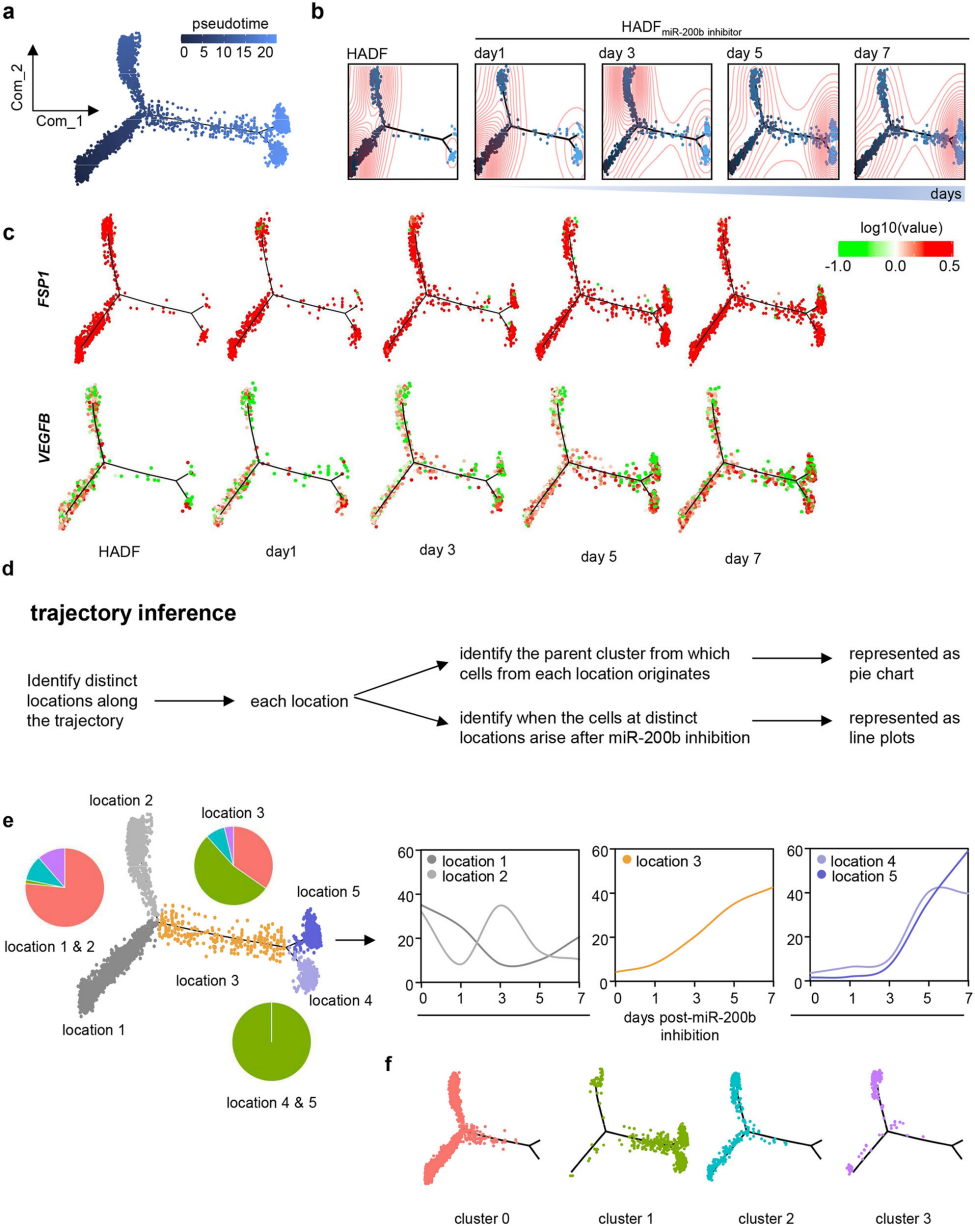
Single-cell trajectory analysis identify fibroblast subpopulation state change. **a** Single cell trajectory analysis shows distribution of HADF cells presented in Fig. 1B. **b** Pseudotime analyses reveal putative trajectories of HADF acquired before and after inhibition of miR-200b at different time points. Pseudotime ordering on fibroblasts arranged them into two major trajectories, followed by one intermediate branch ending into a bifurcation seen in the vasculogenic fibroblasts. Untreated cells (HADF) were mainly distributed in the left side of the trajectory, while the number of cells increases over time post anti-miR-200b treatment in the intermediate and right side. Red circles represent the cells density estimation. **c** Relative expression level of FSP1 and VEGFB along the trajectory showing that the terminal states maintain expression of FSP1, but the anti-miR-200b transfected cells have higher expression of VEGFB. **d** Schematic representation of the trajectory analysis over different time points. **e** Left panel: Cells were identified to be at 5 locations along the trajectory. Location 1 and 2 which have more cells from the untreated sample contain cells mainly coming from main cluster 0 (f). Locations 4 and 5 which have more cells from the anti-miR-200b treated samples contain cells mainly coming from main cluster 1 (f). The intermediate location (location 3) contains cells mainly coming from main clusters 0 and 1 (f). Right panel: line plots representing the percentage of the originating sample in each location showing that cells at location 3 started rising after day 1 post anti-miR-200b treatment and the cells at 2 terminal locations (4 and 5) are increasing after day 3 post anti-miR-200b treatment. **f** The newly formed cluster is scattered along the intermediate part of the trajectory and along the 2 terminal branches. Cells are color coded based on the originating cluster. Statistical methods are provided in detail in Methods.

Anti-miR-200b treated HADF subsets were subsequently examined by pseudotemporal order based on change of single-cell transcriptomes using monocle 2 (Fig. 3a). We randomly chose 6,000 of the 36,308 cells for trajectory inference (Fig. 3b)^21^. Parental HADF cell density was greatest on the left branches of the trajectory, while anti-miR-200b transfected cells were scattered progressively over the middle and accumulated within the right branches over time (Fig. 3b, c). Compositional analysis of cell trajectory over time (Fig. 3d, e) revealed cluster 0 distributed on left side (locations 1 and 2) (Figs. 1b, 3e), while cells on right side (locations 4 and 5) were solely comprised from cluster 1 cells (Figs. 1b, 3e). To identify reprogramming dynamics, temporal changes in subsets (Figs. 1b, 3e) in each of those 5 locations was quantitated. The percentage of cells at locations 1 & 2 decreased upon anti-miR-200b treatment over time. The percentage of cells along the middle part of the trajectory (location 3) increased after day 1, while cells distributed on the right 2 terminal locations (4 and 5), did not start to increase until day 3 (Fig. 3e, f). These data support the de novo emergence of VF in cluster 1 via an adaptive state change from other non-vasculogenic clusters.

### Further subset identification in cluster 1 cells

**Fig. 4:**
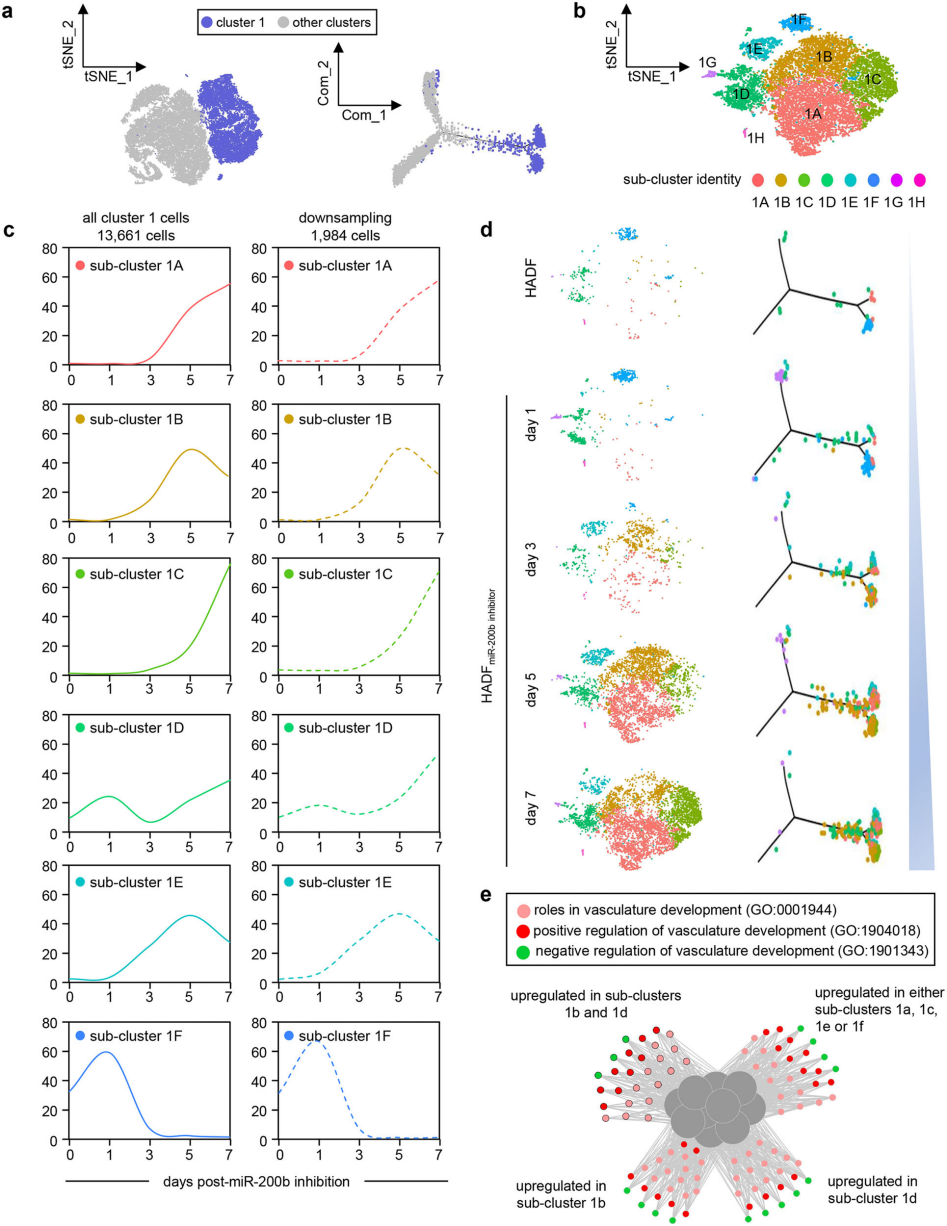
Subclustering of identified cluster 1 of vasculogenic fibroblasts cells showing cells entering into new states over time post miR-200b inhibition. **a** tSNE and psuedotime plots showing identification and selection of cells from cluster 1 colored with violet, while other cells are gray. **b** t-SNE plot showing 8 subclusters identified within vasculogenic cluster 1 cells (from 1A to 1G). **c** Line plots showing the percentage of each subcluster at different time points (parent HADF cells and post anti-miR-200b treated cells at day 1, 3, 5 and 7. Left panel represents line plots from all cluster 1 cells. Right panel represents line plots from the subset of cells that were used for the trajectory inference. **d** t-SNE plots for cluster 1 subclusters for each sample. Subcluster 1 F that was present at the destination location in parent HADF cells and at day 1 post anti-miR-200b treatment faded and was displaced by predominant new subclusters 1B, 1C and 1D at days 3, 5, and 7 post-ASO treatment. **e** Network of the top GO biological processes (with adjusted *p* < 0.05; Bonferroni correction) enriched for the differentially expressed genes (logFC + −0.1; adjusted *p* < 0.05). The center gray circles denote vasculature development, blood vessel development, circulatory system development, blood vessel morphogenesis, tube morphogenesis, angiogenesis, tube development, anatomical structure formation involved in morphogenesis, anatomical structure morphogenesis, regulation of angiogenesis. Genes in the left upper corner with black border were found to be upregulated in subclusters 1B and 1D. Genes in the bottom left and right corners were found to be upregulated in either subcluster 1B or 1D, respectively. Genes in the right upper corner were found to be upregulated in subclusters 1A, 1C, 1E, and 1F. Statistical methods are provided in detail in Methods.

Further clustering of the newly formed VF cells (cluster 1) identified 8 subclusters (1A to 1H) (Fig. 4a, b, Supplementary Fig. 4a). Subclusters with <150 cells (1G and 1H) were excluded, as they represented <1% of cells analyzed. The remaining subclusters (1A to 1F) revealed temporal changes in percentage of each subcluster identified within parent HADF and over time (Fig. 4c, d). Cells assigned to subcluster 1F declined to 1.4% and 0.3% at days 5 and 7 post anti-miR-200b treatment, respectively (Fig. 4c, d), ruling out that the emergence of the cluster 1 state (Fig. 1b) was expanded from the pre-existing 1F parental HADF. Those subclusters of cluster 1 absent in parent HADF that increased after treatment appeared as 1E → 1B → 1 A → 1 C → 1D (Fig. 4c, d). To further identify which VF cluster 1 subclusters were enriched for vasculogenic properties, we employed Gene Ontology (GO) enrichment analysis (EMBL-EBI; homo sapiens) and all subclusters (1A-1F) were enriched in vasculature development, tube development, tube morphogenesis, circulatory system development, blood vessel morphogenesis, and angiogenesis (Fig. 4d, e). To further characterize these subclusters, we represented the expression of genes having roles in vasculature development, positive or negative regulation of vasculature development, and the specific collagen pathway genes identified in Supplementary Fig. 1f (Fig. 4e, Supplementary Fig. 4b, c). Representative genes increased in expression in subclusters 1A-E included cell division cycle 42 (CDC42), integrin beta-1 (ITGB1), and filamin A (FLNA)(Supplementary Fig. 4c); all required for vasculogenic behavior including endothelial cell adhesion, lumen formation, cell-cell junction and/or focal adhesion formation, respectively^22–24^. Both location 4 and 5 (Fig. 3e, f) had similar vasculogenic and positive regulation of angiogenesis pathways enrichment (Supplementary Fig. 4c), however, the bottom branch (location 4) was differentially more enriched in anti-angiogenic genes and collagen formation pathway genes (than location 5) (Supplementary Fig. 4c) reflecting subset heterogeneity. Representative anti-angiogenic genes include serpin family F1 (SERPINF1), Secreted Protein, Acidic and Rich in Cysteine (SPARC), and plasma membrane calcium transporting ATPase 4 (ATP2B4); all known to play roles in anti-angiogenesis^25–27^. The top 23 expressed positive regulators of vasculogenesis, 6 collagen production genes within cluster 1, and 13 other vasculogenic genes of cluster 1 VF (Supplementary Fig. 4b) were subsequently selected as markers for the human VF (Supplementary Table 2) and used to corroborate VF transcriptome emergence in murine skin post-injury and anti-miR-200b treatment. Clear enrichment of subclusters 1 A and 1B were observed over time (Fig. 4b–d), so to determine the origin of these subclusters, cluster 0 was subclustered (see methods for details) into 6 subclusters (0A-F) (Supplementary Fig. 4d). Of interest, subclusters 0B and 0 C did not emerge until day 3 and predominated at day 7 post-transfection (Supplementary Fig. 4e). Trajectory inference was performed in the main clusters and cells were ordered after manually specifying the trajectory roots to start from untreated cells (HADF). Trajectory analysis indicated that subclusters 0B and 0 C are the precursors of subclusters 1 A and 1B (Supplementary Fig. 4f–h). Heat map of some DEG comparing 0B and 0 C with 1 A and 1B are highlighted (Supplementary Fig. 4i). Comparison of other known fibroblast transcripts over the pseudotime related to fibrosis and mechanotransduction was also performed (using published datasets highlighted in methods) with expression levels (Supplementary Table 3) depicting the changes over time from subclusters 0B and 0 C to 1 A and 1B (Supplementary Fig. 4j, k). Finally, we sought to determine how the VF described herein may be communicating with other cells within the wound-edge environment. We downloaded a publicly available single cell RNA-seq dataset sampled from diabetic patients with healing (*n* = 7) and non-healing (*n* = 4) diabetic wound skin tissue from the Gene Expression Omnibus (GEO) with accession number GSE165816 and re-analyzed using Seurat v4.0^28^ package in R. Quality filtered data (full details of data flow in methods) of 33,814 cells were normalized using SCTransform and integrated using reciprocal principal component analysis (RPCA) function in Seurat. Further, unsupervised analysis of the integrated samples was performed using principal component analysis (PCA). Nine clusters of cells were identified and subsequently annotated to different cell types based on the top ten highly expressed and established marker genes documented in PanglaoDB^29^ (Supplementary Fig. 5a). Cluster 0, consisted of 9981 cells expressing DCN, COL1A1, and COL1A2, was characterized as fibroblast cells and was further subjected to subcluster analysis at 0.1 resolution and visualized in UMAP plot (Supplementary Fig. 5a, b). Signature genes of VF cells investigated across these subclusters identified seven genes significantly abundant in subcluster 0 (student’s *t*-test, *p* ≤ 0.01, log2 fold change >0.30) compared to other subclusters (Supplementary Fig. 5c). Distribution of cells representing the VF score (computed based on significant VF genes, see methods) was illustrated in UMAP plot (Supplementary Fig. 5d). Based on VF score, the fibroblast subclusters were further classified into VF and nonVF cells as shown in respective UMAP plots (Supplementary Fig. 5e, f). Bar graph showing the total number of interactions and weighted interaction strength of VF and nonVF included connectome using CellChat package in R (Supplementary Fig. 5g). The inferred cell-cell communication networks were compared and differential connectome of VF over nonVF was illustrated in network plot (Supplementary Fig. 5h). CellChat derived communication network of VF included connectome and nonVF included connectome were shown as communication network (Supplementary Fig. 5i). Top 10% differential connectome from the analyses further indicate strong interaction of VF with endothelial cells (cluster 3), smooth muscle cells 1-2 and basal cells/keratinocytes. Top four signaling pathways included noncanonical WNT (ncWNT), heparin sulfate proteoglycans (HSPG), agrin (AGRN) and WNT were significantly enriched only in VF included connectome (Supplementary Fig. 5j).

### miR-200b inhibition de-silences FLI1 and triggers a vasculogenic state change

To identify molecular targets of miR-200b that induce the VF state change, we performed in silico analyses using TargetScan, miRanda, and Diana-MicroT algorithms. Given miR-200b has numerous target genes^30,31^, we focused on identifying transcription factors known to be important for fibroblast to endothelial conversion, such as FLI1 and ETV2^9,32^. The 3′-untranslated region (3′-UTRs) of FLI1 transcription factor contained binding sites for miR-200b and FLI1 was validated to increase in VF (Fig. 2a and Supplementary Fig. 6a), but no miR-200b binding sites to ETV2 3′-UTRs were identified. MiR-200b inhibitor and mimic were validated in treated HADF (Supplementary Fig. 6b, c), then shown to significantly modulate FLI1 expression (Supplementary Fig. 6d). Delivery of miR-200b mimic significantly suppressed FLI1-3′-UTR reporter luciferase activity (Supplementary Fig. 6e), but this effect was blocked using a mutated FLI1-3′-UTR lacking miR-200b binding sites (Supplementary Fig. 6a–e). To examine the significance of FLI1^33^ in inducing a vasculogenic state change caused by miR-200b inhibition, HADF were transfected with either anti-miR-200b or FLI1 siRNA alone or in combination (Supplementary Fig. 6f). FLI1 knockdown significantly blunted the ability of miR-200b inhibition to upregulate FLI1 expression compared to HADF treated with the combination of siRNA-control and anti-miR-200b inhibitor (Supplementary Fig. 6f). Significant functional abrogation of the increased capillary tube and branching formation induced by treating HADF with the miR-200b inhibitor was demonstrated by FLI1 knockdown (Supplementary Fig. 6g). Finally, overexpression of FLI1 in HADF significantly increased mean tube length and branching nodes compared to plasmid control treated HADF (Supplementary Fig. 6h). These observations confirmed that the vasculogenic state change induced by miR-200b inhibition in HADF in vitro was FLI1-dependent and led to lineage tracing studies in transgenic mice for in vivo validation.

### Lineage tracing mouse studies for direct in vivo activation of a fibroblast state change

**Fig. 5:**
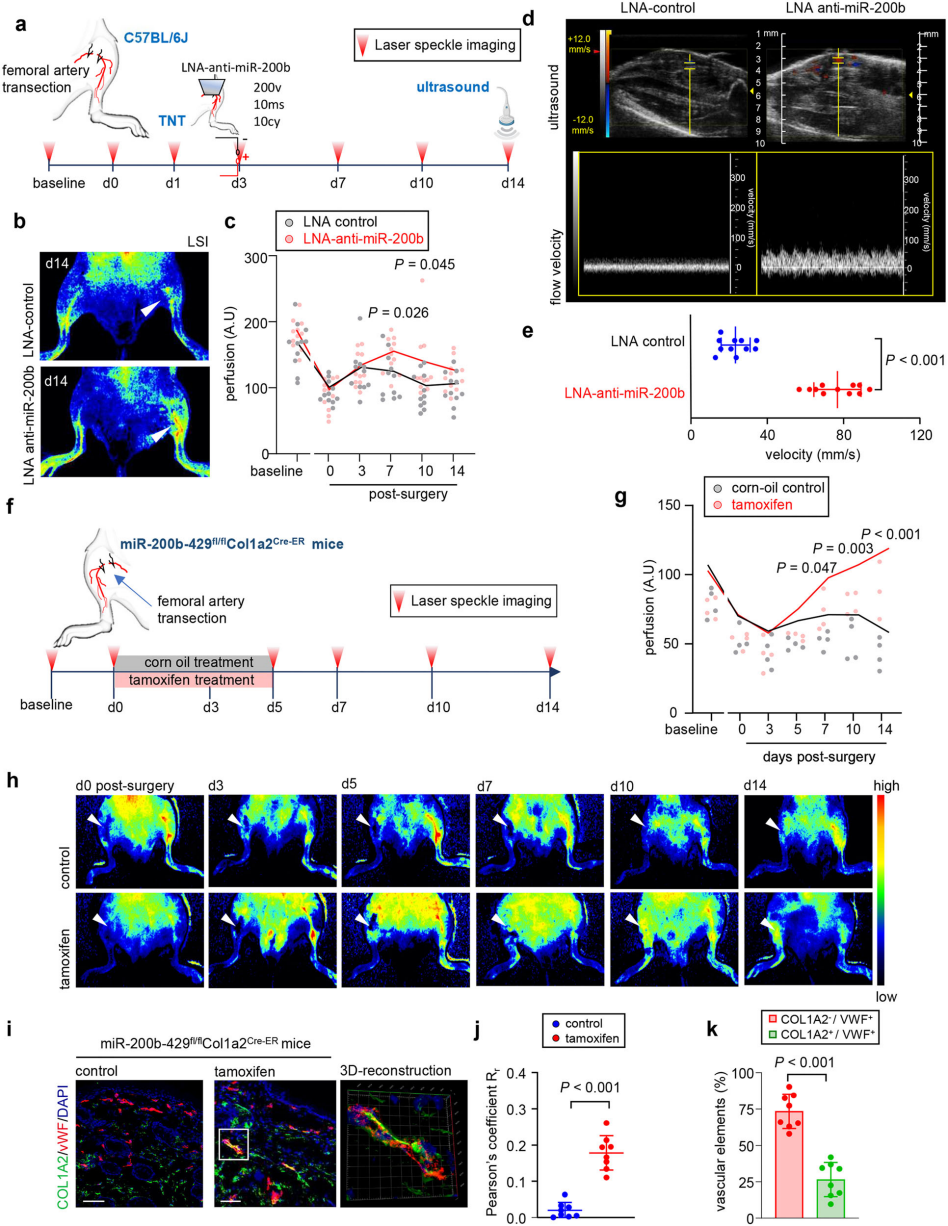
In vivo vasculogenic fate change of dermal fibroblasts by wound induced suppression of miR-200b. **a** Schematic diagram showing hind-limb ischemia and tissue nanotransfection technology in C57BL/6 mice. **b**, **c** Representative day 14 laser speckle image (b) and quantification (**c**) of hind limb perfusion at different time points post-surgery in C57BL/6 mice treated with either control LNA or LNA-anti-miR200b by tissue nanotransfection. The lines represent the mean, and the dots represent the individual value (*n* = 11). **d**, **e** Representative ultrasound and flow velocity images (d) and quantification of flow velocity (**e**) of feeder vessels supplying blood to hind limb in C57BL/6 mice treated with either control LNA or LNA-anti-miR200b post-hindlimb surgery (*n* = 11). **f** Schematic diagram of hind-limb (HL) surgery experiment in miR-200b-429^fl/fl^Col1a2^creER^ mice. **g**, **h** Hind-limb perfusion (**g**) and representative images (**h**) of corn oil (control) and tamoxifen treated miR-200b-429^fl/fl^Col1a2^creER^ mice at different time points post-surgery (*n* = 4). **i** Immunofluorescence confocal image of day 14 wound-edge tissue in miR-200b-429^fl/fl^Col1a2^creER^ mice stained for COL1A2 and vWF. The right panel shows the 3D-rendering of the inset in tamoxifen treated day 14 miR-200b- 429^fl/fl^Col1a2^creER^ mice. Scale, 100 µm. **j** Colocalization of COL1A2 and vWF was determined by Pearson correlation (r). Data expressed as mean ± S.D (*n* = 8). **k** The COL1A2 and vWF colocalized vascular elements were quantified in tamoxifen treated miR-200b-429^fl/fl^Col1a2^creER^ mice and plotted graphically. Data expressed as mean ± S.D (*n* = 8). Data in **c**, **e**, **g**, **j**, **k** were analyzed by two-tailed unpaired Student’s *t* test. Fig 5a, f were created with BioRender.com.

Skin injury (Supplementary Fig. 7a) diminished abundance of miR-200b^8^, however, FLI1 abundance concomitantly peaked at day 9 (Supplementary Fig. 7a, b). To test the therapeutic significance of miR-200b inhibition, we studied the dysregulated physiology of the ischemic hind limb of instrumented C57BL6 mice (Fig. 5a). TNT-based anti-miR-200b delivery, rescued the induced hindlimb ischemia with significantly improved perfusion as measured by laser speckle imaging (Fig. 5b, c) and high-resolution ultrasound (Fig. 5d–e). Interestingly, significant increases in CD31 + vWF+ vascular elements emerged in the ischemic tissue with little to no contribution of other non-endothelial local cells such as keratinocytes and macrophages (Supplementary Fig. 7c, e). To search for direct evidence of VF cell state conversion in vivo, ischemic hind limb studies were performed in a fibroblast restricted inducible miR-200b-429^fl/fl^-Col1a2^CreER^ mouse strain (Supplementary Fig. 7f–h); the Col1a2^CreER^ transgenic mouse was chosen since this gene was the most highly expressed collagen gene in cluster 1 VF (Fig. 1c, d). Tamoxifen treatment following surgery induces Cre recombinase in Col1A2+ fibroblasts and excision of miR-200b-429 (Fig. 5f); an effect not seen in corn oil treated controls (Fig. 5f). Tamoxifen-treated conditional knockout of miR-200b in fibroblasts enhanced perfusion of the ischemic limb by day 5 post-surgery (Fig. 5g, h). This rescue was associated with increased abundance of vWF+ VF (Fig. 5i-j) which comprised up to 25% of the total vascular elements of the tissue (Fig. 5k). In a second approach, the miR-200b-429^fl/fl^-Col1a2^CreER^ mice were pretreated for 5 days (day −10 to −5) with tamoxifen or control corn oil (Supplementary Fig. 7i) before artery removal. The purpose of this study was to confirm significant loss of miR-200b in dermal fibroblasts isolated by laser capture microdissection (LCM) (Supplementary Fig. 7j) prior to induction of the ischemic injury (to assure Col1a2 driver expression was restricted to dermal fibroblasts and not reflective of post-wounding Col1a2 upregulation in other lineages, like activated endothelial cells or macrophages^34,35^). In the tamoxifen pretreated miR-200b-429fl/fl-Col1a2CreER mice (Supplementary Fig. 7i), perfusion increased significantly by day 3 post-ischemic injury compared to corn oil treated mice and remained increased throughout the two-week study (Supplementary Fig. 7k, l). Confirmation for VF origin from Col1A2 expressing fibroblast cells was obtained by breeding ROSA^mT/mG^ mice with miR-200b-429^fl/fl^-Col1a2^CreER^ mice (Supplementary Fig. 7h). In these mice, tamoxifen treatment not only leads to Cre excision of miR-200b-429 but also the constitutive expression of ROSA driven tdTomato in fibroblasts with onset of permanent GFP expression. Indeed, as above (Supplementary Fig. 7k, l), tamoxifen treatment led to significantly enhanced perfusion (Supplementary Fig. 7m, n) in animals subjected to ischemic injury. This rescue was associated with increased abundance of GFP^+^CD31^+^ VF (Supplementary Fig. 7o) which comprised up to 26% of the total vascular elements of the tissue (Supplementary Fig. 7o). Additional support for these results were also obtained by using a second fibroblast lineage tracing mouse strain (*Fsp1-Cre*:R26R^tdTomato^)^36^ in which tdTomato reporter expression in skin is constitutively expressed in fibroblasts; FSP1 was validated to be expressed by all cluster 1 VF cells (Fig. 1c). These mice were transfected with locked nucleic acid (LNA) anti-miR-200b topically delivered by TNT 24 h post-surgical removal of the femoral artery (Supplementary Fig. 7q). On day 5 post-injury, wound-edge tissue showed marked emergence of fibroblasts with near undetectable miR-200b abundance (Supplementary Fig. 7r, s). Wound-edge tdTomato+ fibroblasts displayed emergence of the endothelial marker CD31 (Supplementary Fig. 7t, u) and tdTomato^+^CD31^+^ co-expressing VF comprised nearly 30% of the total CD31^+^ elements in tissue (Supplementary Fig.7v) corroborating the level of vessel contribution of VF in the treated miR-200b-429^fl/fl^-Col1a2^CreER^ mice (Fig. 5i–k). Of interest, if these same mice were subjected to excisional wounding but transfected with control oligonucleotide or miR-200b mimic, significant increase in VF were seen in control treated and significantly diminished VF were quantified in miR-200b mimic treated murine wound-edge tissue (Supplementary Fig. 7w). Thus, lowering of miR-200b abundance within fibroblasts, using genetic or topical approaches results in substantial in vivo activation of fibroblasts into a VF state that increases tissue perfusion. Whether deeper skeletal muscle fibroblasts or adventitial femoral artery fibroblasts contributed to this improved recovery cannot be ruled out in this hindlimb model that involves injury to skin and femoral artery and diminished perfusion to muscle tissue. Thus, we subsequently employed a full thickness cutaneous injury model to examine the role of anti-miR-200b treatment and impact on skin perfusion and wound closure.

### FLI1 required for in vivo reprogramming of dermal fibroblasts into vasculogenic fibroblasts

To test the role of FLI1 in the VF state conversion at the site of injury, a Cre/loxP regulated RNA interference approach was utilized to obtain conditional Fsp1 fibroblast-specific gene knockdown in mice^37^. Four loxP flanked Fli1 shRNA expression cassettes were validated to knockdown FLI1 expression (Supplementary Fig. 8a–c) in murine dermal fibroblasts in vitro and 3 were pooled and used for lentiviral transduction at the wound-edge of *Fsp1-Cre*:R26R^tdTomato^ wounded mice (Supplementary Fig. 8d). Anti-sense inhibition of miR-200b (in combination with control shRNA LV) in wound-edge tissue significantly enhanced perfusion and wound closure (Supplementary Fig. 8e, f). In contrast, miR-200b inhibitor administration with concomitant FLI1 knockdown restricted to the wound-edge fibroblasts, significantly diminished wound perfusion (Supplementary Fig. 8e) and impaired wound closure (Supplementary Fig. 8f). These findings mirror the human HADF responses to miR-200b inhibition in vitro (Supplementary Fig. 6a, e–g), but raised the question if the human VF gene expression profile identified in vitro (Supplementary Table. 2) was reflected in the murine dermal VF emerging in vivo?

### Topical anti-miR-200b-LNA induces emergence of vasculogenic fibroblasts in cutaneous wounds

**Fig. 6:**
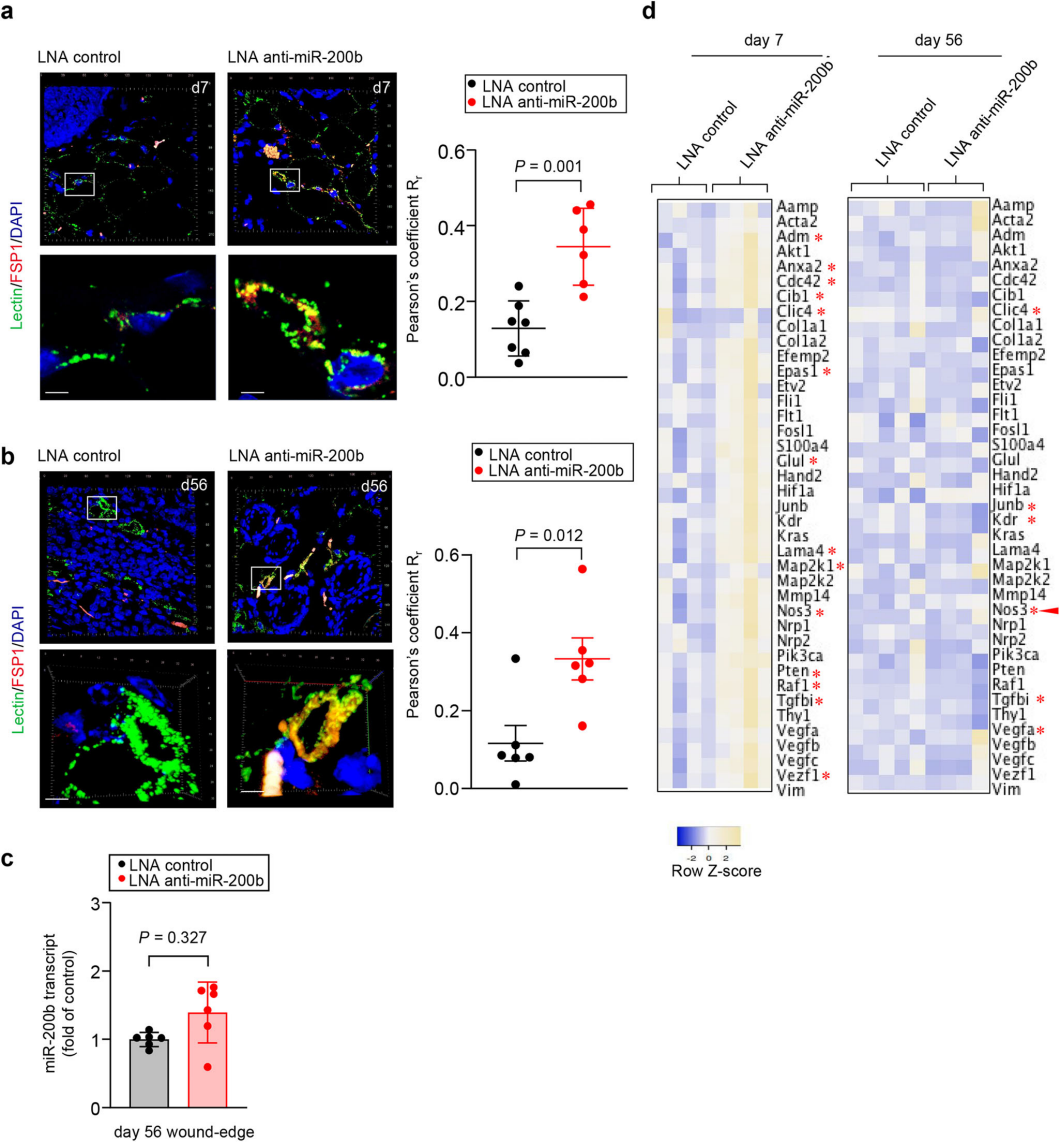
Topical LNA-anti-miR-200b induces emergence of vasculogenic fibroblasts in cutaneous wounds. **a**, **b** Immunofluorescence confocal image of day 7 wounds in Fsp1-Cre tdTomato mice treated with LNA control and LNA anti-miR 200b inhibitor showing lectin perfused Fsp1 positive vessels. Scale, 20 µm. The images showing the colocalization of lectin and Fsp1 in the 3D cross-sectional view rendered by Zen software (*n* = 6–7). **c** miR-200b expression in wound-edge tissue of Fsp1-Cre tdTomato mice treated with LNA control and LNA anti-miR 200b inhibitor at day 56 (*n* = 6–7). **d** Heat map showing gene expression in laser captured samples in Fsp1-Cre tdTomato mice treated with LNA control and LNA anti-miR 200b inhibitor at day 7 and day 56 (d7, *n* = 4; d56, *n* = 5) The red asterisks indicate the gene that were upregulated at day 7 and day 56. The red arrow indicates gene that were upregulated both in day 7 and day 56. *p < 0.05. Data expressed as mean ± S.D. Data in **a**–**d** were analyzed by two-tailed unpaired Student’s *t* test.

*Fsp1-Cre*:R26R^tdTomato^ mice were subjected to paired punch biopsy wound induction on the dorsum (Supplementary Fig. 7a). Anti-miR-200b-LNA was delivered by TNT and animals were sacrificed at 7 and 56 days. While some physiologic emergence of tdTomato^+^ cells that co-localized with lectin perfused vessels was apparent, as might be expected, treatment with anti-miR-200b significantly increased such events at day 7 (Fig. 6a). TdTomato^+^ VF persisted in the anti-miR-200b treated wounds into the tissue remodeling phase at day 56 post-wounding (Fig. 6b) despite the miR-200b abundance returning to normal (Fig. 6c). Murine VF (tdTomato^+^lectin^+^) (day 7 and 56) were recovered by LCM and RNA isolated for assay of the panel of transcripts identified in cluster 1 VF cells (Supplementary Table 2). Numerous transcripts were identified at day 7 and 56 in the murine VF that corroborated the cluster 1 panel generated in vitro for a human VF state (Fig. 6d). Of interest, the expression of collagen producing genes were similar in sham control and treated murine VF at day 7 and 56 (Fig. 6d). In contrast, numerous vasculogenesis genes were significantly higher in the day 7 treated animals compared to controls, but the number of such genes diminished in the day 56 treated animals indicating a change in the VF state even though these cells persisted within perfused vessels in the remodeling tissue (Fig. 6b).

### Topical anti-miR-200b-LNA rescues delayed diabetic wound closure

**Fig. 7:**
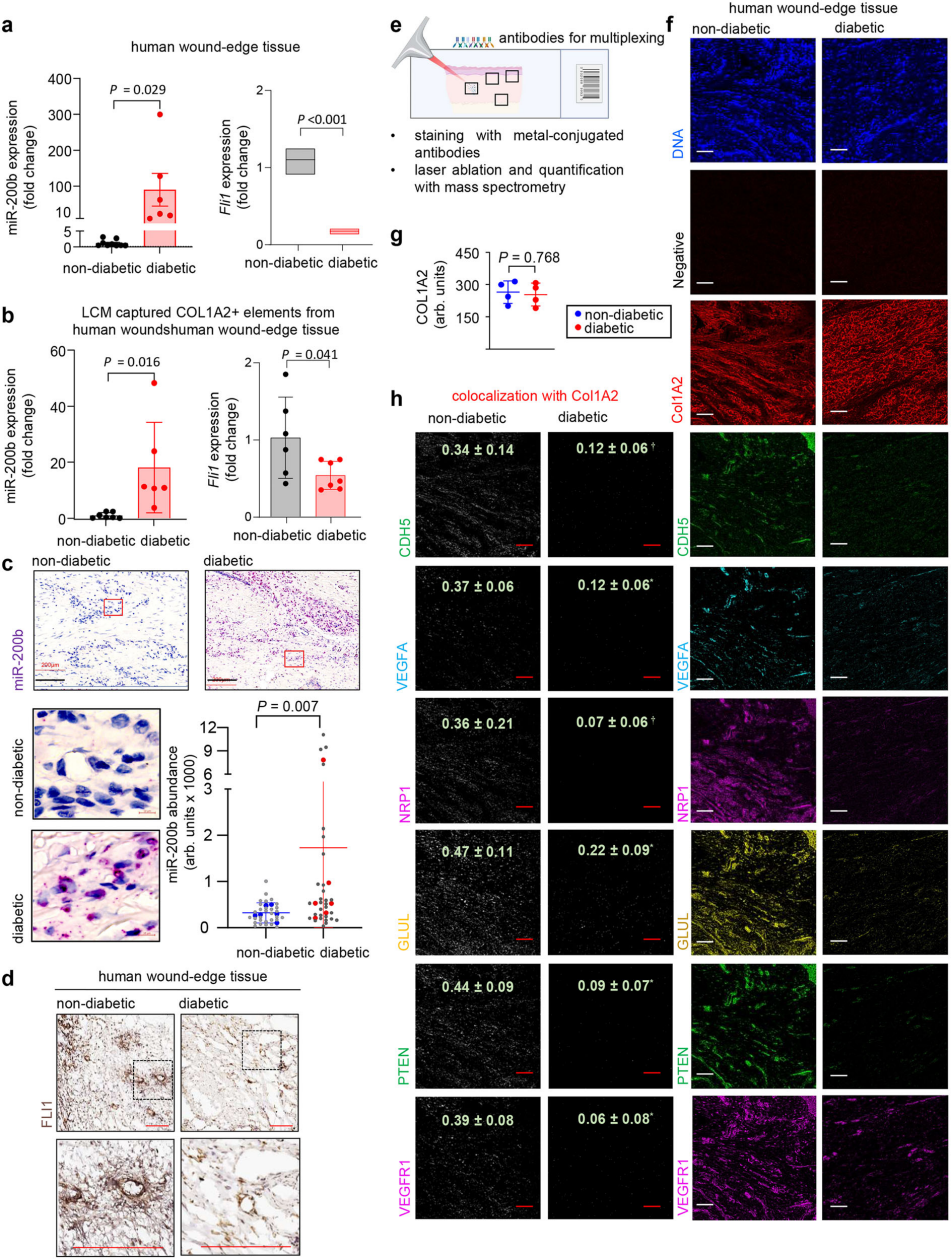
Topical LNA-anti-miR-200b induces emergence of vasculogenic fibroblasts in cutaneous wounds. **a**, **b** miR-200b (left) and *FLI1* (right) expression in human wound-edge tissue (**a**) and LCM captured COL1A2^+^ elements (b) from non-diabetic and diabetic subjects. Data expressed as mean ± S.D (*n* = 6). The line inside the box represents the mean in Fig. 7a (right). **c** Representative in situ staining of miR-200b (top) with higher (bottom) magnification and quantification in human wound-edge tissue of non-diabetic and diabetic subjects. Scale, 200 µm (*n* = 6). In the graph, each dot corresponds to one quantified ROI, except the blue and red dots, which correspond to the mean of each human subjects. At least 4 ROI per section. **d** Representative immunohistochemistry of FLI1 represented in lower (top) and higher (bottom) magnification in human wound-edge tissue of non-diabetic and diabetic subjects. Scale, 200 µm. **e** Schematic procedure to acquire highly multiplexed IMC from diabetic and non-diabetic wound-edge tissue. **f** Distribution of signal in a spatial context. The intensity of each antibody were displayed alone. DNA was used and positive control. Scale bar, 20 μm (*n* = 4). **g** The intensity of COL1A2 in both non-diabetic and dibetic human wound-edge was found to be comparable (*n* = 4). **h** The colocalization on COL1A2 with other vasculogenic fibroblasts markers were analysed in image J using color thresholding and extracted as white dots on the image. The number represents the Pearson’s correlation coefficeint quantified in image J using Coloc2 plugin. Data expressed as mean ± S.D. Data in **a**, **b**, **c**, **g**, and **h** were analyzed by two-tailed unpaired Student’s *t* test. Fig 7e created with BioRender.com.

**Fig. 8:**
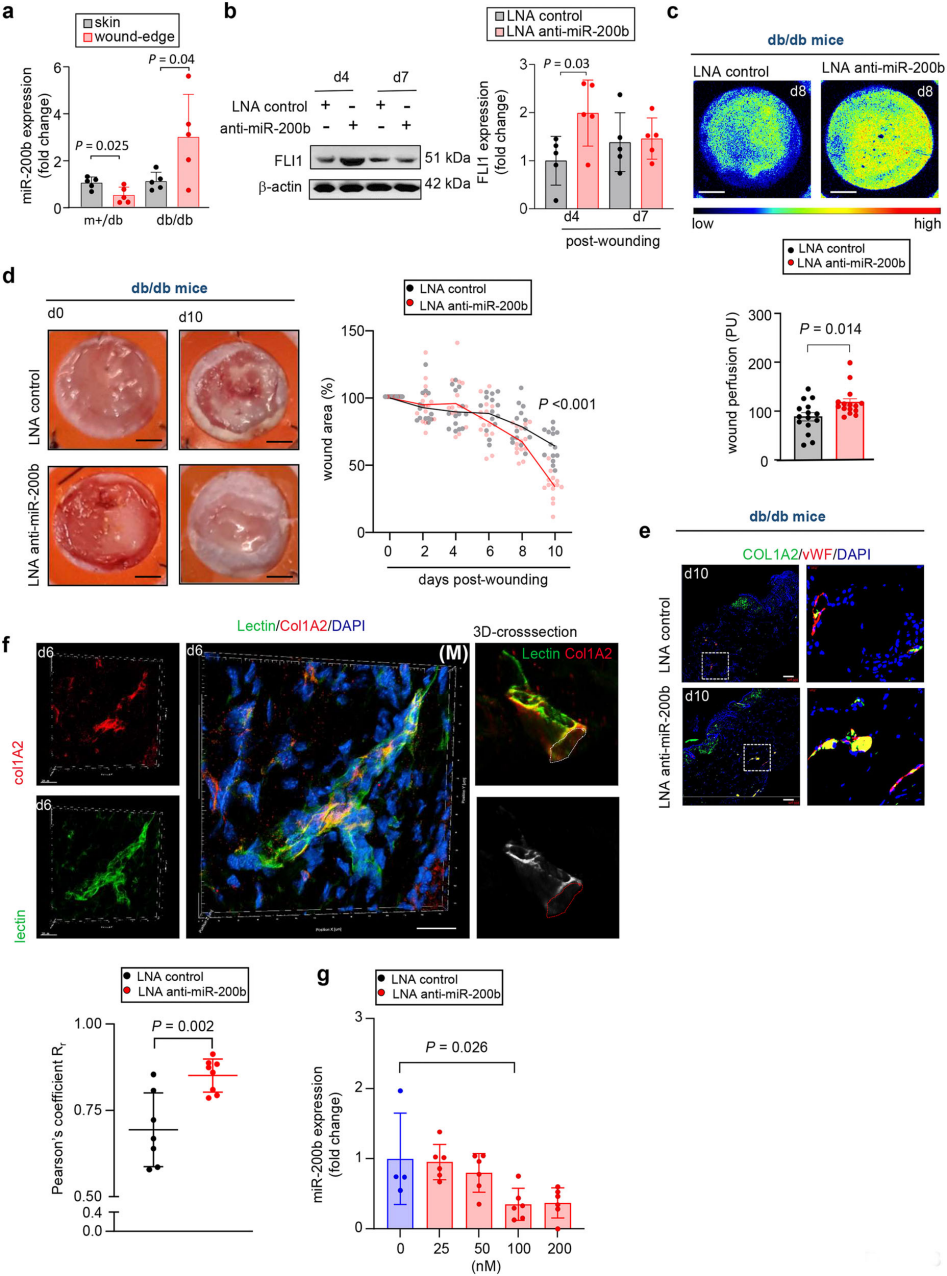
Topical anti-sense oligonucleotide inhibition of miR-200b at wound-edge improves diabetic wound healing. **a** miR-200b expression in skin and wound-edge tissue of non-diabetic (db/+) and diabetic (db/db) mice. Data expressed as mean ± S.D (*n* = 5). **b** Western blot analysis and densitometric quantification of FLI1 expression at wound-edge tissue of db/db mice. β-actin serves as a loading control. Data expressed as mean ± S.D (*n* = 5). **c** Representative cutaneous blood perfusion images and quantification at day 8 wound-edge tissue of db/db mice treated with either LNA-control or LNA-anti-miR-200b inhibitor. Scale, 2 mm (*n* = 15). **d** Digital photograph of db/db mice wound-edge tissue at day 0 and day 10 treated with either LNA-control or LNA-anti-miR-200b inhibitor. Scale, 2 mm. Digital planimetry of the wound area was quantified using ImageJ software and plotted graphically. The line represents the mean wound area (*n* = 14). **e** Immunofluorescence confocal image of day 10 wound-edge tissue in db/db mice stained for COL1A2 and vWF. Colocalization of COL1A2 and vWF was determined by Pearson correlation (r). Results represent mean ± S.D (*n* = 7,8). **f** Immunofluorescence confocal image of day 6 wounds in db/db mice treated with LNA-anti-miR-200b inhibitor showing lectin perfused Col1A2 positive vessles. Scale, 20 µm. The left panels show individual channels. The middle panel shows the 3D-reconstruction image of the perfused vessels. The right panels showing the colocalization of lectin and Col1A2 in the 3D cross-sectional view rendered by IMARIS (Bitplane) software (M), indicate movies for that frame in the supplement (Movie S1). **g** miR-200b expression at day 5 wound-edge tissue of diabetic (db/db) mice using different doses of LNA-anti-miR-200b. Data expressed as mean ± S.D (*n* = 4–6). Data in **a**–**d**, **f** were analyzed by two-tailed unpaired Student’s *t* test. Data in **g** was analyzed by one-way analysis of variance with the *post-hoc* Bonferroni multiple comparison test.

Impaired wound healing is a common and major diabetic complication^38^. Compared to non-diabetic human subjects, the wound-edge tissue of diabetic patients showed remarkably elevated miR-200b abundance while *FLI1* mRNA and protein abundance were concomitantly low (Fig. 7a). LCM captured COL1A2 expressing fibroblasts in the wound-edge also displayed elevated miR-200b abundance and diminished *FLI1* expression (Fig. 7b) and wound-edge in situ hybridization displayed significantly higher miR-200b nuclear localization in diabetic than in non-diabetic human subjects (Fig. 7c). Significantly less FLI1 expression was noted in diabetic than non-diabetic wound tissue (Fig. 7d), Numerous cell surface proteins identified in the list of VF proteins (based on Supplementary Table 2) reflecting endothelial antigens, but not COL1A2), were significantly diminished in wound-edge of diabetic subjects compared to non-diabetic subjects (Fig. 7e–g and Supplementary Fig. 9a). Co-localization of COL1A2 and all the endothelial antigens (evidence of VF) revealed significantly diminished VF in diabetic compared to non-diabetic tissues (Fig. 7h). Demographics of the study patients are displayed (Supplementary Fig. 9b). To test this pathway in diabetic mice, leptin receptor deficient B6.BKS.Leprdb/db(db/db) mice^39^ served as our type II diabetic model and non-diabetic m^+^/db littermates as controls (Supplementary Fig. 10a). In db/db mice, wounding failed to suppress miR-200b expression at the wound-edge (Fig. 8a) compared to the m^+^/db controls at day 3, though miR-200b abundance at the wound-edge returned to normal skin abundance by day 7 and retained this level until day 11 (Supplementary Fig. 10b) and FLI1 abundance was low days 7–11 in db/db compared to control mice (Supplementary Fig. 10c). TNT delivered anti-miR-200b caused lowering of miR-200b abundance at the wound-edge of db/db mice for up to 7 days post-wounding (Supplementary Fig. 10d), increased expression of FLI1 (Fig. 8b), increased perfusion at the wound site (Fig. 8c and Supplementary Fig. 10e) and enhanced wound closure (Fig. 8d) overcoming the delayed wound healing of db/db mice. The TNT delivery of the anti-miR-200b oligonucleotide also led to increased abundance of Col1A2^+^vWF^+^ fibroblasts (Fig. 8e) and increases in Col1A2^+^ VF comprising blood vessels that were documented to be perfused with intravenously injected lectin in the db/db mice (Fig. 8f, Supplementary Movie 1). Importantly, TNT delivery of the LNA anti-miR-200b was optimized at 100 nM dosing (Fig. 8g). TNT delivery of the anti-miR-200b oligonucleotide in control m^+^/db non-diabetic mice also significantly increased perfusion to the wounds (Supplementary Fig.10f), enhanced wound closure (Supplementary Fig. 10g), and emergence of VF (Supplementary Fig. 10h). TNT delivery of Fli1 plasmid alone significantly increased perfusion (Supplementary Fig.10i) and wound closure in db/db mice (Supplementary Fig. 10j) compared to control plasmid. Confirmation of the improved wound healing in the db/db mice with the anti-miR-200b TNT treatment was documented by significant increases in collagen deposition at the wound site (Supplementary Fig. 10k) and quantitative analytical histologic support for enhanced wound closure (Supplementary Fig. 10l–p). Thus, a single administration of anti-miR-200b oligonucleotide via TNT to the wound-edge of diabetic db/db mice significantly enhanced wound closure via emergence of vasculogenic VF that improved perfusion to the wound site.

## Discussion

Fibroblasts were identified more than 150 years ago, but these resilient and adaptive extracellular matrices (ECM) producing and remodeling cells, have challenged scientists to find unambiguous lineage specific markers that define them^13,40–42^. In the present work, we combined single-cell RNA sequencing of anti-miR-200b treated HADF with in vivo lineage specific murine fibroblast functional and molecular analysis and identified a transcriptomic signature of human VF that was corroborated in murine dermal fibroblasts. This VF population was dependent upon FLI1 expression and deletion of FLI1 abundance in dermal fibroblasts significantly blunted the anti-miR-200b induced increase in skin wound tissue perfusion and healing.

TNT is an electromotive gene transfer technology that delivers plasmids, RNA, and oligonucleotides to live tissue in vivo causing direct conversion of tissue function^9,43–47^. Topical TNT treatment with a single anti-miR-200b in skin augmented the dysregulated physiologic cellular state change in diabetic mice and resulted in emergence of a VF subset that promoted improved perfusion of the injured tissue (via formed vessels) and overcame delayed wound healing. The present results demonstrate participation of VF as part of a defined physiologic response to cutaneous injury via formation of perfused blood vessels and clarify the mechanism as a FLI1 transcription factor dependent state, not fate, change.

## Methods

### Study approvals

All human tissue-based experiments were reviewed and approved by Institutional Review Board (IRB) of Indiana University (IRB # 1906520102, 1807426080, 1505714405, and 12516) or The Ohio State University (IRB # 2004H0278). Declaration of Helsinki protocols was followed, and patients gave their written informed consent. Additionally, under IRB–approved protocols, surgically discarded and de-identified samples were collected. This included the collection of wound tissue obtained from individuals undergoing elective surgeries. In the cases of surgically discarded and de-identified samples, informed consent was not required from the subjects as per federal guidelines category # 4 listed in 45 CFR 46.101(b)(1) through 46.101(b)(6).

All animal studies were performed in accordance with protocols approved by the Laboratory Animal Care and Use Committee of Indiana University and The Ohio State University. Animals were euthanized by inhalation of 100% CO2 overdose delivered in an induction chamber at a controlled rate of such that 30–50% of the volume of the chambers is displaced per minute.

### Reagents

All tissue culture materials were either obtained from Gibco-BRL/Life Technologies, Gaithersburg, MA, or Lonza, Allendale, NJ. Low Density Lipoprotein from Human Plasma, Acetylated, Alexa Fluor 594 Conjugate (Alexa Fluor 594 Ac-LDL) (cat. no. L35353) and Calcein AM (cat. no. C3099) were purchased from Molecular Probes, Thermo Fisher Scientific, Waltham, MA. Cultrex PathClear Reduced Growth Factor BME (cat. no. 3433-005-01) was purchased from R&D Systems, Minneapolis, MN. Secrete-Pair Dual luminescence assay kit (cat. no. SPDA-D010), and SimpleChIP Plus Enzymatic Chromatin IP Kit (Agarose Beads) (cat. no. 9004) were purchased from GeneCopoeia from Cell Signaling Technology (Danvers, MA) respectively. Streptozocin (cat. no. S0130) and any other reagents unless mentioned were procured from Sigma, St. Louis, MO. A list of all the primers is provided in Supplementary Table 1.

### Non-viral Tissue nanotransfection device fabrication

Tissue nanotransfection (TNT) devices were fabricated from thinned (~200 μm) double-side polished (100) silicon wafers using standard cleanroom fabrication technologies. Briefly, a ~1.5 μm thick layer of AZ5214E was spin coated on wafer surface. Nanopores were subsequently patterned on the photoresist via projection lithography. Such pores were then used as etch masks to drill ~10 μm deep nanochannels on the silicon surface by deep reactive ion etching (DRIE) using a combination of SF6/C4F8 gases. Microscale reservoirs were then etched on the backside of the wafers via contact photolithography and DRIE in order to gain fluidic access to the nanochannels. Finally, a ~50 nm thick insulating layer of silicon nitride was deposited on the wafer surface^45–48^. Because this approach does not require any laboratory infrastructure, it may be applied at the point-of-care with minimal training^46,49^.

### Cell culture and in vitro nanoelectroporation

Primary human adult dermal fibroblasts (ATCC, Manassas, VA, cat. no. PCS-201-012) were expanded in fibroblast basal medium (ATCC cat. no. PCS-201-030) supplemented with fibroblast growth kit-serum-free (ATCC, cat. no. PCS-201-040) containing Penicillin-Streptomycin (10,000 U/mL) solution (Gibco/Life Technologies, Waltham, MA, cat. no. 15140122) at 37 °C in humidified chamber consisting of 95% air and 5% CO_2_. Human dermal microvascular endothelial cells (HMECs)^50^ were cultured in MCDB-131 medium (Gibco/Life Technologies, cat. no. 10372-019) supplemented with 10% FBS, 10 mM L-glutamine and 100 IU/mL penicillin and 0.1 mg/mL streptomycin.

In vitro nanoelectroporation was conducted via 3D Nanochannel Electroporation (NEP) as described previously^51^. Briefly, the cells were first grown to full confluency overnight on the 3D NEP device. Subsequently, a pulsed electric field was used to deliver control or miR-200b inhibitor (50 nM) into the cells. The cells were then harvested 24 h after miRNA delivery, placed in EBM-2 basal medium (Lonza, cat. no. CC-3156) supplemented with EGM-2 MV SingleQuot kit components (Lonza, cat. no. CC-414 Supplementary 7) and processed further for additional experiments.

### miR inhibitors/mimic and siRNA transfection

Cells were seeded in 12-well plate at density 0.1 × 10^6^ cells/well in antibiotic free medium for 24 h prior to transfection. When the confluency was approximately 70%, transfection was achieved by liposome-mediated delivery of miR-200b inhibitor (100 nM) or miR-200b mimic (50 nM), or siRNA smart pool for human FLI1 (100 nM) using DharmaFECT 1 transfection reagent (GE Dharmacon) and OptiMEM serum-free medium (Invitrogen, Thermo Fisher Scientific, Waltham, MA)^52,53^. Samples were collected after 72 h of transfection for further experiments.

### Flow cytometry sorting and analysis

HADF cells were transfected with miR-200b inhibitor or control inhibitor through NEP. HADF cells (1 × 10^6^) were harvested before or after control or miR-200b inhibitor transfection, resuspended in PBS-containing 2% FBS and 2 mM EDTA, and then stained with fluorochrome-labeled antibodies (5 μl per test) for 30 min at room temperature (RT). The expression of different fibroblast and endothelial markers in control and miR-200b inhibitor transfected HADF cells were assessed through flow cytometry (BD LSR II, BD Accuri C6 or Aria III flow cytometer) using the following antibodies: APC conjugated anti-human CD144 (VECadherin) (Biolegend, 348508; Clone: BV9; dilution-5 μl per 10^6^ cells), PE conjugated anti-human CD31 (Biolegend, 303106; dilution- 5 μl per 10^6^ cells), PE conjugated anti-human CD309 (VEGFR2) (Biolegend,359904); Clone: 7D4-6; dilution- 5 μl per 10^6^ cells), FITC conjugated anti-human CD90 (Thy1);(Biolegend, 328107; Clone: 5E10; dilution-5 μl per 10^6^ cells), APC conjugated anti-human CD49B (Biolegend, 359310-100 test; dilution − 1:200 per 10^6^ cells); PE anti-human β2-microglobulin (Biolegend, 316306; dilution − 1:200 per 10^6^ cells). BD FACS Diva software v.8 was used for cell sorting. All data were analyzed with FlowJo software (version 10.0.7).

### Ac-LDL uptake assays

HADF cells were transfected with either control or miR-200b inhibitor. On day 7 and day 28, cells were incubated with AlexaFluor 594-labeled Ac-LDL (10 μg/ml) in DMEM at 37 °C for 4 h. On termination of incubations, cells were washed in phosphate buffered saline (PBS) and fixed with 4% paraformaldehyde for 30 min. HMEC were used as positive control cells. The uptake of Ac-LDL was analyzed by fluorescence microscopy using the Zen software (Zeiss)^9,54^.

### In vitro angiogenesis assay

In vitro angiogenesis was assessed by the tube formation ability on Matrigel as described previously^8,54^. Briefly, HADF cells were transfected with either control or miR-200b inhibitor. After day 7 and day 28 post-transfection, 0.05 × 10^6^ cells were seeded on 4-well plates pre-coated with Matrigel. HMEC were used as positive control cells. The angiogenic property was assessed by measuring the tube length after 8 h of cell seeding using the Zen software (Zeiss).

### 3D Collagen lumen assay

For in vivo assay, the collagen plugs were prepared of final 1 ml volume with 1 million miR-200b inhibitor treated-GFP-HADF cells mixed with equal number of tdTomato-ECFCs. Briefly, GFP-HADF cells (GFP Expressing Human Dermal Fibroblasts-Adult, Angio-proteomie, Cat no. cAP-0008-adGFP) were transfected with miR-200b inhibitor for 7 days. The tdTomato ECFC (Endothelial colony forming cells) were used as positive control and were mixed in equal number with miR-200b inhibitor treated-GFP-HADF in collagen matrices (2 mg/ml Rat tail type I collagen; Corning cat. no. CB-40236), 150 µg/ml human fibronectin (Gibco cat no. 33016015), 1.5 mg/ml Sodium bicarbonate (Gibco), 10% FBS, 25 mM HEPES (Gibco cat. no. 15630-080), 30% EBM-2 supplemented with cEGM-2 and 10% FBS. Additionally, 200 ng/mL Stromal Cell-Derived Factor 1α/pre-B Cell Growth Stimulating Factor human (SDF-1 alpha, Sigma cat. no. S1577) was also added to the gel^55^. Both the cells mixed in 1:1 ratio, suspended in collagen matrix on ice, pipetted into wells of a 4 well plate (100 µl/well) were allowed to polymerize at 37 °C for 30 min. The prepared collagen plugs were inserted into the abdomen of NSG mouse (32 weeks). After 28 days, the NSG mice were perfused with lectin conjugated with Alexa flour 647. The collagen plug was surgically removed and fixed in 4% paraformaldehyde before imaging. The collagen plug was imaged using LSM 880 with Airyscan (Carl Zeiss, Jena, Germany).

### Morphometric analysis of vessel networks

For morphometric analysis of vessel networks, the collagen tissue constructs were fixed in 3% paraformaldehyde. The constructs were imaged using confocal microscopy (Zeiss LSM 880) in combined fluorescence and reflection modes for visualization of lumen formation. Image stacks were collected, and the image files were imported into Imaris (Bitplane) for 3D reconstruction^56^.

### Single cell RNA-sequencing analysis

#### Raw data processing and quality control

CellRanger v3.0.2 (http://support.10xgenomics.com/) was utilized to process the raw sequence data generated. Briefly, the FASTQ files were aligned to the human reference genome hg38 with RNAseq aligner STAR. The gene expression level of individual genes were quantified based on the number of UMIs (unique molecular identifiers) detected in each cell. The generated filtered gene-cell barcode metrices were used for further analysis. Seurat package (v.3.1.1) in R (v.3.5.1) was used for preprocessing and visualization^28,57,58^. The initial dataset contained 40,212 cells from 5 samples (HADF and 4 samples transfected with anti-miR-200b at day 1, 3, 5, and 7). Gene expression values were log normalized and scaled to 10,000 transcripts per cell. The top 2000 variable genes were identified. Then, principal component analysis (PCA) was performed. For quality control, cells with more than 10% mitochondrial RNA, less than 200 or more than 8000 detected transcripts and cells with less than 500 or more than 60,000 of total number of counts were excluded. Additionally, genes that were detected in fewer than 3 cells were excluded. After quality control, 36,308 cells were maintained for downstream analysis. PCA was performed again after filtration and the top 15 principal components were used for clustering the cells, resulting in four main clusters.

#### Identification of transcriptional changes after anti-miR-200b transfection

Wilcoxon Rank Sum test was used to perform DEG analysis. The overall transcriptional change, resulting from anti-miR-200b transfection, was identified by comparing gene expression in all the cells in each time point (day 1, day 3, day 5 and day 7 post anti-miR-200b transfection) with the parent HADF cells. Genes with logFC + −0.2 chosen to calculate pathways activity in each anti-miR-200b treated sample using Reactome^59^. After clustering and identification of cluster 1 as a newly formed cluster, we identified the differentially expressed genes in each cluster by comparing each one of the 4 resulting clusters with the rest of the cells (log fold change + −0.1, adjusted *p* < 0.05 and the gene to be found in at least 20% of the cluster cells or the rest of the cells).

To account for the effect of transfection, additional samples were analyzed using control transfection (miRIDIAN microRNA hairpin inhibitor negative control; control inhibitor, CI) along with miRIDIAN microRNA hsa-miR-200b-3p hairpin inhibitor (MI) at two different time points (d1 and d7 post-transfection). Cells with a total number of detected genes between 200 and 8,000; total number of counts between 500 and 60,000; and mitochondrial gene expression percentage less than 10% were retained for the downstream analysis. Next, normalization was performed using log transformation with 10,000 as a scaling factor. The top 2000 variable genes across all samples were used to perform PCA. Then, the first 15 dimensions were used to find neighbors and clustering using 0.05 as a resolution. Cluster 1 was validated to be consistent with cluster 1 in Fig. 1b by expression level of COL1A1- (log2FC −1.85), COL3A1- (log2FC −1.48), COL5A2- (log2FC −0.86) and VEGFB + (log2FC 0.33). The differential expression analysis was performed using the Wilcoxon Rank Sum test to compare gene expression differences between (i) all cells (MI day 1 versus CI day 1 and MI day 7 versus CI day 7) and (ii) at the level of cluster 1 identified in the main Fig. 1b (MI day 1 versus CI day 1 and MI day 7 versus CI day 7).

#### Identification of small subset of cells might have a role in vasculature development after anti-miR-200b transfection

To reveal heterogeneity within the new cell population (cluster 1), another round of clustering using higher resolution (0.25) was performed after subsetting cluster one cells from the rest of the clusters using all the samples combined. This analysis identified 8 distinct clusters reflecting distinct functional identities. Clusters with less than 150 cells (6 and 7) were removed and then the differentially expressed genes between subclusters were identified using Wilcoxon Rank Sum test in which each cluster was compared to the rest of the cells (same cutoff values as for main cluster markers identification). Vasculature development GO term (GO:0001944; The process whose specific outcome is the progression of the vasculature over time) was downloaded from EMBL-EBI database including 392 genes for homo sapiens to check the involvement of cluster 1 subclusters in the vasculature process. We first identified the differentially expressed genes among each subcluster using same parameters as main clusters analysis. We next checked the percentage of upregulated and downregulated genes found in the GO term vasculature development in each subcluster with positive and negative markers. Additionally, GO:1904018 (166 genes) for positive regulation of vasculature development, and GO:1901343 (115 genes) for negative regulation of vasculature development were used to classify the differentially expressed genes among the subclusters to generate the heatmap. To check what other biological processes the resulting genes (100 genes that were found differentially expressed between subclusters and having a role in vasculature development, positive or negative regulation of vasculature development) were enriched using g:profiler^60^ using Bonferroni correction for multiple testing correction. Cytoscape (v3.7.1) was used to visualize the resulting top 10 biological processes along with the genes^61^.

#### Subclustering of cluster 0 cells and identification of precursor for cluster 1

To investigate in detail, cells attributed to cluster 0 (17,483 cells, Fig. 1b) were extracted from the Seurat object. The top 2000 highly variable genes among cluster 0 cells were identified and scaled. Then, PCA was performed, and the top 15 dimensions were used for plotting tSNE and clustering using resolution 0.3. The second round of clustering was performed to identify the heterogeneity of the cluster 0 cells, which resulted in 6 sub-cluster (0-A to 0-F). Interestingly, the sub-clusters 0 C and 0B emerged at day 3 onwards. After subclustering the cluster 0 cells, we aimed to identify the precursors of the cluster 1 cells within the subclusters of the cluster 0. Trajectory inference was performed including all cells from the 4 main identified clusters using Monocle 3 library in R to learn a principal graph describing the transition between cluster 0 and cluster 1 subclusters (v1.0.0). PCA was performed on the log normalized gene expression values using 20 dimensions followed by UMAP. The function learn graph was used to identify possible paths cells can utilize while transitioning between different states. Cells were ordered after manually specifying the trajectory roots to start from untreated cells.

#### Pseudotime analysis reveals new states transition post anti-miR200b transfection

Monocle package in R was used to infer the trajectory and to order the cells across the pseudotime. It uses a reversed graph embedding algorithm to identify the sequence of change in gene expression each cell must go through across developmental processes^21^. To determine the genes to be used for ordering the cells, pseudo-bulk comparison was performed between all the cells in day 7 versus all the HADF cells using the top 2000 variable genes across cells from all samples. This resulted in identification of 579 differentially expressed genes to be used for ordering (minimal log fold change + −0.1, adjusted *p* < 0.05 and the gene to be expressed in at least 20% of either HADF or day 7 post anti-miR200b transfected cells). A sample of 6000 cells was randomly picked from all 36,308 cells. We then calculated the percentage of cells in each location along the trajectory to identify when parent HADF cells started shifting from old states to new ones. To identify cell state dynamics during state transition (cluster 1 subclusters), we calculated the percentage of cells found in each of the subclusters within each sample (parent and treated cells). We performed this analysis using all the cells 36,308 or using the sample of 6000 cells to validate their representation of the whole population. We then ordered the subclusters rising overtime based on the clusters which reached >30% increase first compared to their percentage at time zero (parent HADF). Next, to identify the classic fibroblast transcripts related to fibrosis and mechanotransduction along the pseudotime, a combined score was calculated using the AddModuleScore function in Seurat for skin fibrosis (gene set taken from Chang Gu et al. 2020)^62^, and mechanotransduction (gene set taken from Xingchen Li et al. 2020 and Fabiana Martino et al. 2018)^63,64^. Gene list used for plotting these scores on pseudotime along with their expression levels is provided in the supplementary information (Supplementary Table 3). For skin fibrosis score, 34,430 cells had score >0, while 1878 cells had score <0. For mechanotransduction score, 36,248 cells have score >0, while 60 cells have score <0.

#### Cell-cycle scoring

To check the cycling difference between the vasculogenic cluster and parent cluster different clusters post miR-200b inhibition, cell cycle analyses were performed to identify the ratio of dividing cells and non-proliferating cells within clusters 0 and 1. Assigning cell cycle phase for each cell within clusters 0 and 1 was performed using the function CellCycleScoring in Seurat^28,65^. The canonical marker genes for S and G2/M phases as previously published were used to calculate cell cycle scores^66^. Cells expressing markers for either S or G2/M phases were assigned to their corresponding phase (high proliferation score), while cells not expressing mitotic marker genes were assigned to G1 phase. Next, the number of cells in each cell cycle phase within clusters 0 and 1 was counted. There was no significant change was observed between the cells present in proliferation phase (G2M and S phase) between the clusters 0 and 1 (Ordinary two-way ANOVA test, *p* > 0.05).

#### CellChat analysis

To investigate the cell communication of VFs in other cell types at the wound-edge, we utilized the publicly available single cell RNA-seq dataset of 14 samples from 11 diabetic patients with healing (*n* = 7) and non-healing (*n* = 4) diabetic foot ulcer (DFU). Processed single cell datasets were downloaded from Gene Expression Omnibus (GEO) with accession number GSE165816 and re-analyzed using Seurat v4.0 package in R^28^. Downloaded samples were examined for read quality in R and stored as Seurat objects. Cells with high mitochondrial content, that depicts the mitochondrial read contamination or dying status, were computed using “PercentageFeatureSet” function in Seurat. Quality filtering of the samples was performed using “subset” module in Seurat. Cells qualifying multiple thresholds including mitochondrial content <15%, cells expressing >200 genes, and genes uniquely expressed in >3 cells were considered for downstream analysis. Quality filtered data of 33814 cells were normalized using the SCTransform and integrated using reciprocal principal component analysis (rpca) function in Seurat. Further, unsupervised analysis of the integrated samples was performed using principal component analysis (PCA). Principal components thus obtained were projected to identify the cluster of cells using “FindNeighbors” followed by “RunUMAP” (Uniform Manifold Approximation and Projection) and “FindClusters” (at 0.25 resolution) analyses in Seurat. Nine cluster of cells were identified and subsequently annotated to different cell types based on the top ten highly expressed and established marker genes documented in PanglaoDB^29^. Cluster 0, consisted of 9981 cells expressing DCN, COL1A1 and COL1A2, was characterized as fibroblast of human diabetic wound samples. This cluster was isolated using “subset” module in Seurat. To further identify the distribution of VF cell population in isolated fibroblast cluster, sub-clustering analysis was performed. Three fibroblast subclusters resulting from subcluster analysis at 0.1 resolution were visualized in UMAP plot. Signature genes of VF cells investigated across these subclusters identified eight genes significantly abundant in subcluster 0 (student’s *t*-test, *p* ≤ 0.01, log2 fold change >0.30) compared to other subclusters. These significant genes were selected to characterize VFs as a particular cell state across fibroblast subclusters by using “AddModuleScore” function of Seurat and VF score was computed. Based on the VF score, the fibroblast was further subset into VF like (VF = 33.64% or 3358 cells) and non-VF like (non-VF = 66.36% or 6623) cells.

Next, to investigate the role of VF in communication with the other cell types identified in DFU samples, CellChat was employed^67^. The connectome thus established for VF with cell types was compared with non-VF cells directed connectome with other cell types. Top 10% differential connectome from the analyses further indicated strong interaction of VF with endothelial cells (cluster 3), smooth muscle cells 1–2 and basal cells/keratinocytes. These connectomes were further explored for significantly enriched signaling pathways ranked based on relative information flow (cumulative interaction probability among all pairs of cell types).

### miRNA target luciferase reporter assay

HADF cells were transfected with 100 ng of human *FLI1*–3′UTR or a mutant vector for 48 h using Lipofectamine LTX/Plus reagent^68^. Firefly luciferase was cloned under the control of cytomegalovirus (CMV) promoter. For normalization, cells were co-transfected with Renilla plasmid (10 ng). Cells were lysed, and luciferase activity was measured using dual-luciferase reporter assay system (Promega, Madison, WI) according to manufacturer’s protocol. Data were presented as ratio of firefly to Renilla luciferase activity (FL/RL).

### Human samples

Human skin and wound biopsy samples were obtained from healthy adult human subjects or chronic wound patients. The wounds are interpreted as chronic if not closed within 4 weeks of onset^69^.

### Animal and in vivo gene delivery

Male C57BL/6 mice (8–12 weeks old) were obtained from Harlan Laboratory, Indianapolis, IN. Mice homozygous (BKS.Cg-m^+/+^Lepr^db/J^, or db/db; stock no 000642) for spontaneous mutation of the leptin receptor (Lepr^db^) or their respective non-diabetic lean control littermates m + /db (8–12 weeks old) were obtained from Jackson Laboratory, Bar Harbor, ME. FSP1-Cre mouse was a kind gift from Dr. Arjun Deb (University of California, Los Angeles, California 90095, USA). *Fsp1-Cre* mice were crossed with the R26RtdTomato mice (JAX) carrying floxed tdTomato allele. Since, FSP1 is specifically expressed in fibroblasts, the progeny of these mice (*Fsp1-Cre*:R26R^tdTomato^) would have the red fluorescent protein tdTomato expressed specifically in the fibroblasts^36^. Both male and female *Fsp1-Cre*:R26R^tdTomato^ mice (8–12 weeks old) were used in the experiment. Floxed miR-200b-429 mice (miR-200b-429^fl/fl^ were generated by introducing *loxP* sites around miR-200b-429 gene in C57BL/6 J mice purchased from Jackson Laboratory (Bar Harbor, Me). Fibroblast-specific miR-200b knockout mice were generated by breeding our miR-200b-429^*fl/fl*^ with tamoxifen-inducible Col1a2-CreER mice (Stock: 029235, Jackson Laboratories) to obtain fibroblast specific inducible miR-200b knockout progeny [miR-200b-429^fl/fl^-Col1a2^CreER^]. For lineage tracing experiments, miR-200b-429^fl/fl^-Col1a2^CreER^ mice were crossed with ROSA^mT/mG^ mice (Stock: 007576, Jackson Laboratories).

For inducible miR-200b knockout in fibroblasts, Tamoxifen (Sigma-Aldrich, St. Louis, Mo.) was dissolved in equivolume mixture of 100% ethanol and corn oil (Ward’s Science, Canada) to achieve a concentration of 200 mg/ml followed by incubation at 70 °C for 1 h. Unilateral hind-limb ischemia was induced via occlusion and subsequent transection of the femoral artery followed by transection as describe previously^23^. miR-200b deletion was induced in miR-200b-429^fl/fl^-Col1a2^CreER^ or miR-200b-429^fl/fl^-Col1a2^CreER^ ROSA^mT/mG^ mice by applying tamoxifen, or corn oil as control. Tamoxifen or corn oil was applied on ischemic hind limb after the surgery for 5 consecutive days (50 µl/animal). Mice were monitored closely for any adverse reactions to tamoxifen treatment. Perfusion in the hindlimbs was checked by laser speckle during and after ischemic surgery as well as post tamoxifen treatment for 14 days.

*Fsp1-Cre:*R26R^tdTomato^ mice were made diabetic by intraperitoneal injection of streptozotocin (STZ; 50 mg/kg body weight for 5 days) or the vehicle, citrate buffer (0.05 M sodium citrate, pH 4.5) and blood glucose levels were assessed regularly using Accu-Chek glucometer (Roche, Basel, Switzerland). Mice with blood glucose levels >20 mmol/L were defined as diabetic and chosen for experiments. The animals were tagged and grouped randomly using a computer-based algorithm (www.random.org). None of the animals with appropriate genotype were excluded from the study.

Animals were naired 48 h prior to the transfection. Solutions containing either miRCURY LNA microRNA Power Inhibitors of miR-200b or negative control were loaded (100 nM) in the reservoir and tissue nanotransfection was performed as described previously^9^. Delivery of shRNA lentiviral particles (LV) (1×10^7^cfu/mL; 50 µl per wound) was achieved by intradermal injection.

### Murine hind-limb ischemic surgery and wound creation

Mice (8–10 weeks old) were anesthetized with 1–3% isoflurane for hind-limb ischemic surgery and wounding experiments. Unilateral hind-limb ischemia was induced via occlusion and subsequent transection of the femoral artery as described previously^9,70^. Briefly, proximal and distal end occlusions of femoral artery were induced with 7-0 silk suture followed by complete transection. Laser speckle imaging was conducted 2 h post-surgery to confirm successful blood flow occlusion.

For wound experiments, two 6 mm circular excisional wounds were created on the dorsal skin by a punch biopsy, equidistant from the midline and adjacent to the 4 limbs. The wounds were splinted with a silicon sheet to prevent contraction thereby allowing wounds to heal through granulation and re-epithelialization^9,54,71^. Each wound was digitally photographed, and area was analyzed by the ImageJ software. Skin from age-matched unwounded animals served as controls.

The animals were euthanized at the indicated time and wound-edges were collected for analyses. For wound-edge harvest, 1–1.5 mm of the tissue from the leading edge of the wounded skin was excised around the entire wound. The tissues were snap frozen and collected either in 4% paraformaldehyde or in optimal cutting temperature (OCT) compound.

### Laser speckle perfusion imaging

Perfusion imaging was performed using Laser Speckle Perfusion imaging system (Perimed Inc., Sweden). Color coded perfusion maps were acquired at all time points and average perfusion was calculated using PimSoft v1.4 software (Perimed Inc., Sweden)^72^. The wound-edge and wound bed tissue regions were chosen as region of interests (ROI). From the real-time graphs obtained, time-of-interest (TOI) was chosen to include lower peak regions and to exclude motion related artifacts. The mean and standard deviation of perfusion data were obtained from the selected TOI perfusion data.

### Ultrasound based imaging and characterization of blood vessels

Vevo-2100 system (Visual Sonics, Toronto, ON, Canada) was used to obtain ultrasound images of newly formed blood vessels on color-mode with a MS550D linear array probe^54,73–75^. Doppler color flow imaging was implemented to monitor and quantify blood flow characteristics under systole and diastole.

### Laser capture microdissection (LCM)

Laser capture microdissection was performed using the laser microdissection system from PALM Technologies (Bernreid, Germany) as described previously by our group^53,68,76^. Briefly, sections were stained with hematoxylin for 30 s, subsequently washed with DEPC-H_2_O and dehydrated in ethanol as described previously^77^. Dermal fibroblasts rich fraction was identified based on morphology, cut and captured under a 20X ocular lens. Fibroblast from *Fsp1*-*Cre*:R26R^tdTomato^ mice were captured based on the red fluorescence. The samples were catapulted into 25 μl of cell direct lysis extraction buffer (Invitrogen). Approximately 10,00,000 μm^2^ of tissue area was captured into each cap and the lysate was then stored at −80 °C for further processing.

### RNA extraction and real-time quantitative PCR

RNA from cells or wound edge tissue sample was extracted by using miRVana miRNA isolation kit (Ambion, Thermo Fisher Scientific, cat. no. AM1560) according to the manufacturer’s instructions. The RNA quantity was measured using a NanoDrop ND-1000 spectrophotometer (NanoDrop Technologies, Wilmington, DE), and RNA integrity was checked using RNA6000 NanoAssay on Agilent BioAnalyzer 2100 (Agilent Technologies, Santa Clara, CA)^47^. RNA was reverse transcribed using SuperScript III First-Strand Synthesis System (Invitrogen, ThermoFisher Scientific, cat. no. 18080051). Quantitation of mRNA expression was studied using SYBR green–based real-time PCR (Applied Biosystems) by using gene-specific primers. Melting curve analysis was performed after the final extension to ensure the specificity of the products. miRNA expression was quantified using specific TaqMan assays for miRNAs and the TaqMan miRNA reverse transcription kit (Applied Biosystems, ThermoFisher Scientific, Foster City, CA, cat. no. 4366596) followed by real time PCR using the Universal PCR Master Mix (Applied Biosystems, cat. no. 4304437). 18 S rRNA or U6 snRNA were used as housekeeping control. Relative expression of each gene represented as fold change over the control.

### RT2 profiler PCR array

Forty-four genes were analyzed using the RT2 Profiler PCR Array (Qiagen, USA). Real-time PCR was performed using RT2 SYBR Green/ROX PCR Master Mix (Qiagen). The three steps of the cycling program were 95 °C for 10 min for 1 cycle, followed by 50 cycles of 95 °C for 15 s and 60 °C for 60 s. This process was repeated for 50 cycles using the QuantStudio 3 Real Time PCR (Applied Biosystems, USA).

### In situ hybridization assay and quantification

In situ hybridization of miR-200b was performed using the miRNAscope HD Assay Red (ACD Biosystems, Newark, CA) was performed in FFPE human skin tissues as per manufacturer’s instructions with the following protocol adjustments. The tissues were baked for 1 h at 60 °C before fixation in 12% formaldehyde (2 h at RT). Five micrometers (μm) tissue sections were hybridized with the custom designed probe against miR-200b-3p. Small RNA integrity and signal specificity were confirmed with a positive control probe U6 (PN 727871-S1), and a negative control probe scramble (PN 727881-S1). Images were scanned using Zeiss Axio Scan.Z1 (20X bright field) and analyzed using Zen Blue 2.3 software. miR-200b-3p was successfully detected in the human skin tissue samples and not in the negative control. For quantification, the analysis of the chromogenic miRNAscope substrate was done using QuPath software v0.2.3 (https://qupath.github.io/.;open source for bioimage analysis^78^. Image analyses were done following the workflow guidelines provided by the company and the QuPath’s documentation (https://qupath.readthedocs.io/en/latest/index.html) and introductory video tutorials (https://www.youtube.com/c/QuPath/playlists). Five ROIs from each sample were randomly selected from the dermis for the analysis.

### Imaging mass cytometry

All antibodies in the panel were initially tested by immunofluorescence and conjugated to metals using the Maxpar Multimetal Labeling Kit (Fluidigm) according to the manufacturer’s protocol^79^. Data acquisition was performed on a Helios time-of-flight mass cytometer coupled to the Hyperion Imaging System (Fluidigm). Before laser ablation, optical images of slides were acquired using the Hyperion v1.0.560.6 software. Laser ablation was performed at a resolution of approximately 1 mm and a frequency of 200 Hz.

### Image processing and analysis

The images were rendered and exported using MCD Viewer (Fluidigm). The threshold of the non-diabetic and diabetic sections for each channel were normalized and exported as TIFF images. To reduce the noise-to-signal ratio, all images for every channel were processed by automated LUT enhancement and the intensity per unit area was quantified using ImageJ (v1.53 T, 24 Aug 2022, NIH). The co-localization signal was first adjusted for overlapping color threshold and analyzed using Coloc2 plugin.

### Immunohistochemistry (IHC), immunocytochemistry (ICC) and microscopy

Immunostaining was performed on cryosections of tissue samples using specific antibodies as described previously^74,80^. Briefly, OCT embedded tissue were cryosectioned (10 μm), fixed with cold acetone, blocked with 10% normal goat serum (NGS) and incubated with specific antibodies overnight at 4 °C. For immunocytochemistry, cells (0.1 × 10^6^ cells/well) were seeded on a coverslip, fixed with ICC fixation buffer (BD Biosciences, San Jose, CA; cat. no. 550010) prior to blocking with 10% NGS. IHC and ICC were performed using antibodies against anti-FLI-1 (Abcam, ab15289; dilution- 1:50), anti-S100A4/Anti-FSP-1 (Abcam, ab27957; dilution-1:200), anti-CD105 (Abcam, ab107595; dilution-1:500), anti-CD31 (BD Pharmingen, 550274; dilution-1:200), anti-Vwf (Abcam, ab6994; dilution-1:200), Keratin 14 antibody (Biolegends, 905301; Clone: Poly19053, dilution – 1:200), F4/80 antibody (Biorad, MCA497; Clone: Cl:A3-1, dilution − 1:200). Signal was visualized by subsequent incubation with either biotinylated–tagged for DAB staining and fluorescence-tagged appropriate secondary antibodies [Alexa 488-tagged α-rabbit (Thermo Fisher Scientific; A32731, dilution 1:200); Alexa 568-tagged α-rabbit, (Thermo Fisher Scientific; A11011, dilution 1:200)]. Fluorescent stained slides were counter stained with DAPI. Images were captured by fluorescence microscope (Axiovert 200 M; Carl Zeiss Microscopy GmbH, Germany) or Confocal microscope (Olympus FV1000). Comparative analysis for identifying vasculogenic fibroblasts or endogenous vascular endothelium was performed using Axiovision Rel 4.8 software and Olympus FV1000 software. The number of all CD31^+^ elements or FSP-1^+^CD31^+^ elements (Supplementary Fig. 7v) was counted using the automatic particle counting tool feature of ImageJ after adoption of a fixed threshold for every image.

### Western blots

Western blot was performed as described previously^54,76^. Briefly, known concentration of tissue extract or cell lysates were resolved on SDS-PAGE and transferred to PVDF membranes (GE Healthcare Bio-Sciences, Pittsburgh, PA, cat no. IPVH00010). The membranes were first blocked in 10% skimmed milk and incubated with specific primary antibodies overnight at 4 °C. Signal was visualized using corresponding horseradish peroxidase–conjugated secondary antibody (Amersham; NA934; dilution 1:3,000) and ECL Plus Western Blotting Detection Reagents (Amersham GE Healthcare, Pittsburgh, PA). GAPDH (Sigma, G9295; Clone: 71.1; dilution 1:30,000) or β-actin (Sigma, A5441; Clone: AC-15; dilution 1:5000) served as loading control.

### Quantification, statistical analysis, and reproducibility

Samples were coded and data analysis was performed in a blinded fashion. Student’s *t* test (two-tailed) was used to determine significant differences. Comparisons among multiple groups were tested using analysis of variance (ANOVA). Data are presented as mean ± SEM as reported in figure legends. Comparisons among multiple groups were tested using ANOVA in-built function in GraphPad Prism 8.4.2. *p* < 0.05 was considered statistically significant. All representative data shown in the manuscripts are independently performed at least three times with similar results.

### Reporting summary

Further information on research design is available in the Nature Portfolio Reporting Summary linked to this article.

## Supplementary information

Supplementary Information

Description of Additional Supplementary Files

Supplementary Movie 1

Reporting Summary

## Data Availability

Source data are provided with this paper. The raw single cell RNA sequencing data generated in this study have been deposited in the GEO database under accession number GSE167406. Processed single cell datasets were downloaded from Gene Expression Omnibus (GEO) with accession number GSE165816. The other data that support the findings of this study are available from the corresponding author upon request. Source data are provided with this paper.

## Source data

Source Data

## Code availability

Scripts for the downstream analysis of the single-cell data are available on GitHub (https://github.com/ChandanKSenLab/VF_Project).
