# Supplementary material for: Identification of a physiologic vasculogenic fibroblast state to achieve tissue repair
Source: Nat Commun. 2023 Feb 28;14:1129. doi: 10.1038/s41467-023-36665-z (PMC9975176; doi:10.1038/s41467-023-36665-z)
Supplement: Supplementary file 1 — Supplementary Information [file 41467_2023_36665_MOESM1_ESM.pdf]

Identification of a physiologic vasculogenic fibroblast state to achieve tissue repair

Data Supplement

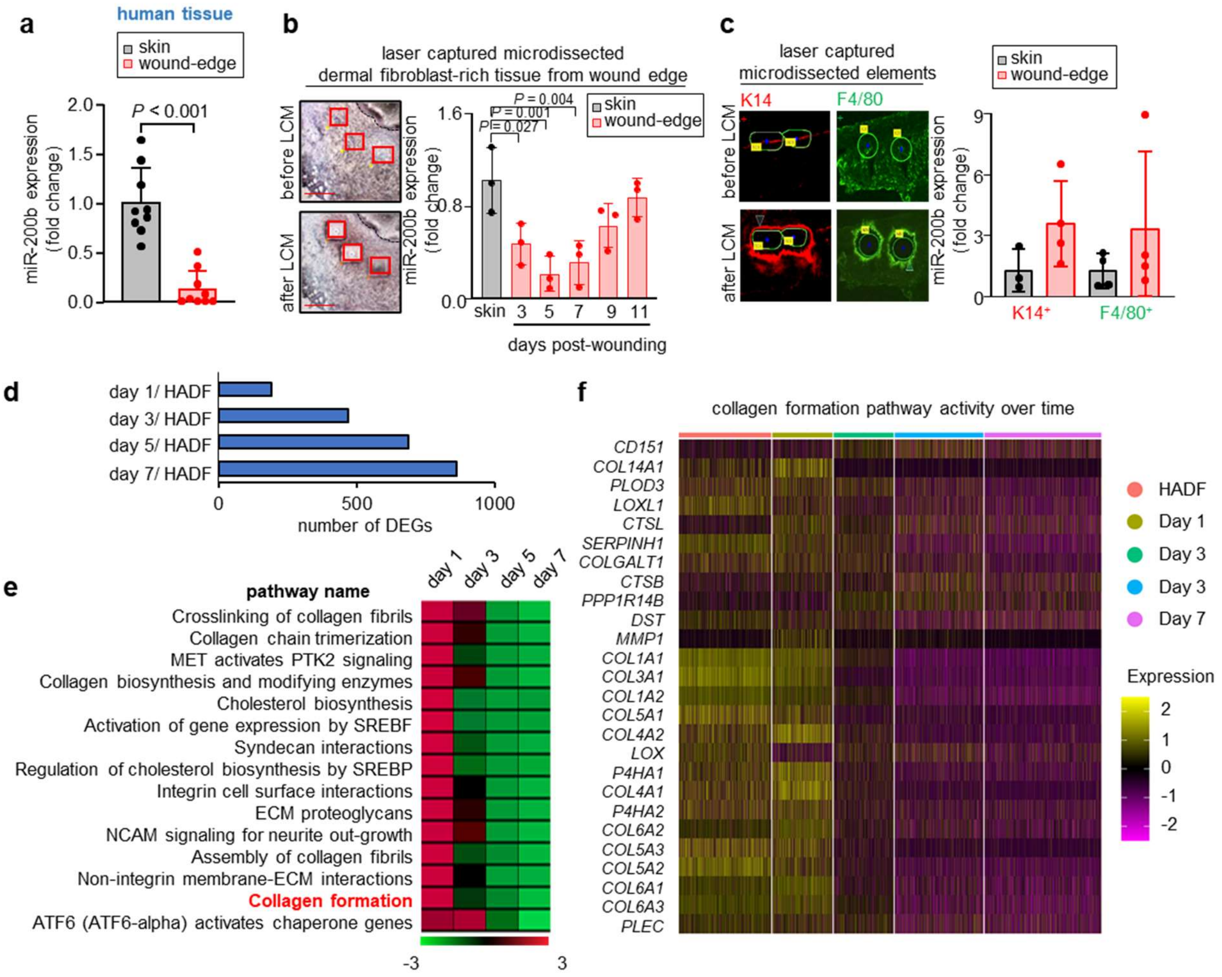

Supplementary Fig. 1 | Direct reprogramming of dermal fibroblasts to endothelial cells by anti-miR200b oligonucleotide. Related to Figure 1. a) miR-200b expression in human skin and wound-edge tissue. Results represent mean ± S.D. (n = 9). b) miR-200b expression in dermal fibroblast-rich skin and wound-edge tissue of C57BL/6 mice collected by Laser Captured Microdissection (LCM) at different days post-wounding. Results represent mean ± S.D. (n = 3). c) miR-200b expression in K14$^+$ (left) and F4/80$^+$ (right) laser captured microdissected elements from skin and d5 wound-edge tissue of C57BL/6 mice. Results represent mean ± S.D. (n = 3- 4). d) Bar plot showing increase in the total number of differentially expressed genes with time for each sample post anti-miR-200b treatment at day 1, 3, 5 and 7 in comparison to parent HADF cells. e) Heatmap of the top 15 downregulated pathways at day 7 post anti-miR-200b treatment showed

downregulation of collagen formation pathway. f) Heatmap representing the relative expression of genes which were enriched in the collagen formation pathway in each sample (before and after anti-miR-200b treatment). Columns represent individual cells. Rows represent genes. Data in a, and c were analyzed by two-tailed unpaired Student's t test. Data in b were analyzed by one-way analysis of variance with the *post-hoc* Bonferroni multiple comparison test.

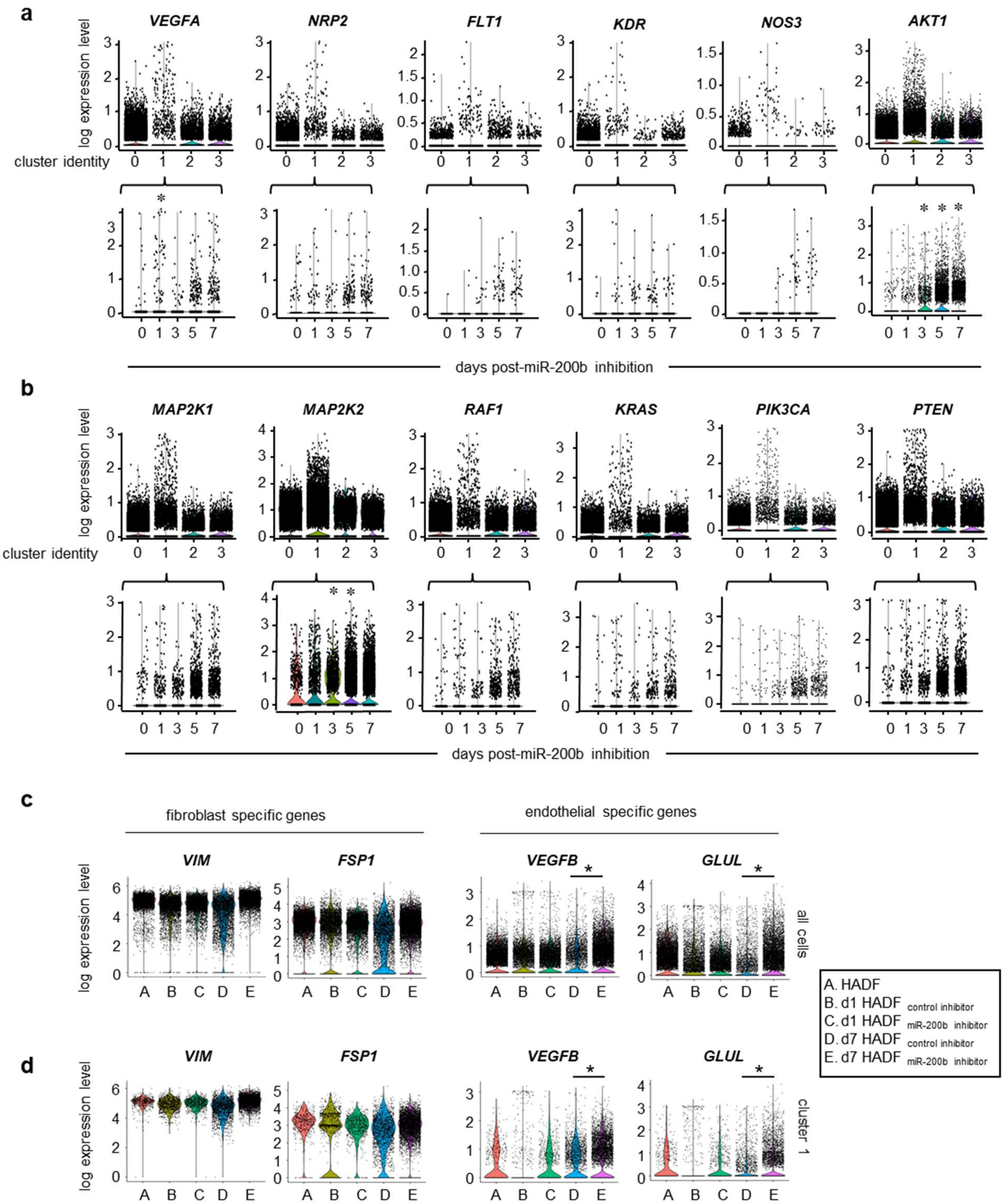
a
VEGFA
NRP2
FLT1
KDR
NOS3
AKT1
log expression level
cluster identity
days post-miR-200b inhibition
b
MAP2K1
MAP2K2
RAF1
KRAS
PIK3CA
PTEN
log expression level
cluster identity
days post-miR-200b inhibition
c
fibroblast specific genes
endothelial specific genes
VIM
FSP1
VEGFB
GLUL
log expression level
all cells
A. HADF
B. d1 HADF control inhibitor
C. d1 HADF miR-200b inhibitor
D. d7 HADF control inhibitor
E. d7 HADF miR-200b inhibitor
d
VIM
FSP1
VEGFB
GLUL
log expression level
cluster 1

Supplementary Fig. 2 | Single-cell analysis reveals temporal upregulation of VEGF pathway in certain clusters of vasculogenic fibroblasts post miR-200b inhibition. Related to Figure 1. a-b) Violin plots showing expression level of VEGF pathway genes in the 4 main clusters (a) and their expression within cluster 1 cells over time post anti-miR-200b treatment (b). c-d) Violin plots showing expression levels of selected fibroblasts and vasculogenic pathway genes in different samples treated with control inhibitor and miR-200b inhibitor at days 1 and 7 in all cells (c) and in cluster 1 (d). * $P < 0.001$ Wilcoxon ranked test.

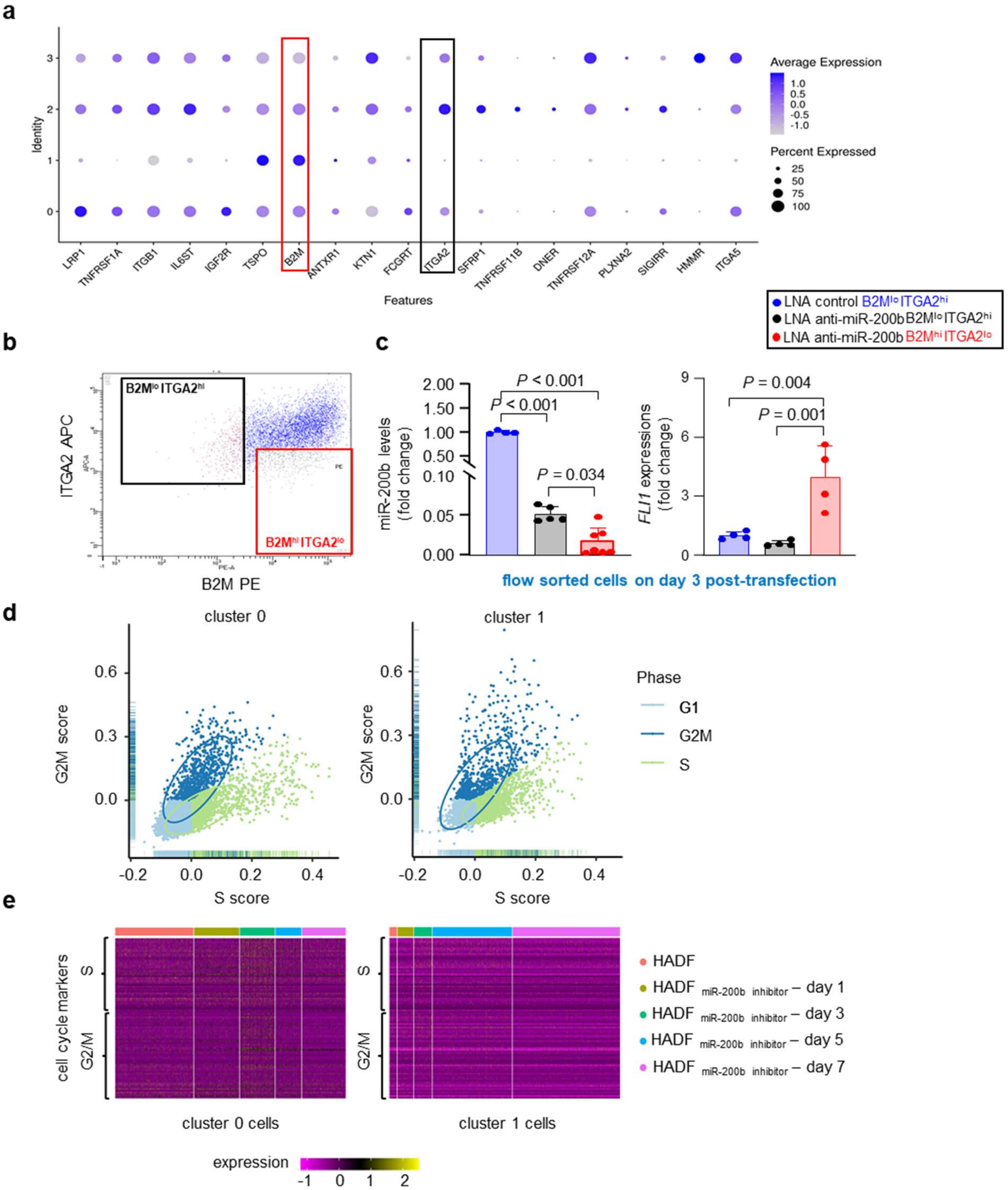

a
Identity
Features
LRP1
TNFRSF1A
ITGB1
IL6ST
IGF2R
TSPO
B2M
ANTXR1
KTN1
FCGRT
ITGA2
SFRP1
TNFRSF11B
DNER
TNFRSF12A
PLXNA2
SIGIRR
HMMR
ITGA5
Average Expression
1.0
0.5
0.0
-0.5
-1.0
Percent Expressed
25
50
75
100
LNA control B2M^{lo}ITGA2^{hi}
LNA anti-miR-200b B2M^{lo}ITGA2^{hi}
LNA anti-miR-200b B2M^{hi}ITGA2^{lo}
b
B2M^{lo} ITGA2^{hi}
B2M^{hi} ITGA2^{lo}
ITGA2 APC
B2M PE
c
miR-200b levels (fold change)
P < 0.001
P < 0.001
P = 0.034
$FLI1$ expressions (fold change)
P = 0.004
P = 0.001
flow sorted cells on day 3 post-transfection
d
cluster 0
cluster 1
G2M score
S score
Phase
G1
G2M
S
e
cell cycle markers
S
G2/M
cluster 0 cells
cluster 1 cells
HADF
HADF miR-200b inhibitor – day 1
HADF miR-200b inhibitor – day 3
HADF miR-200b inhibitor – day 5
HADF miR-200b inhibitor – day 7
expression
-1
0
1
2

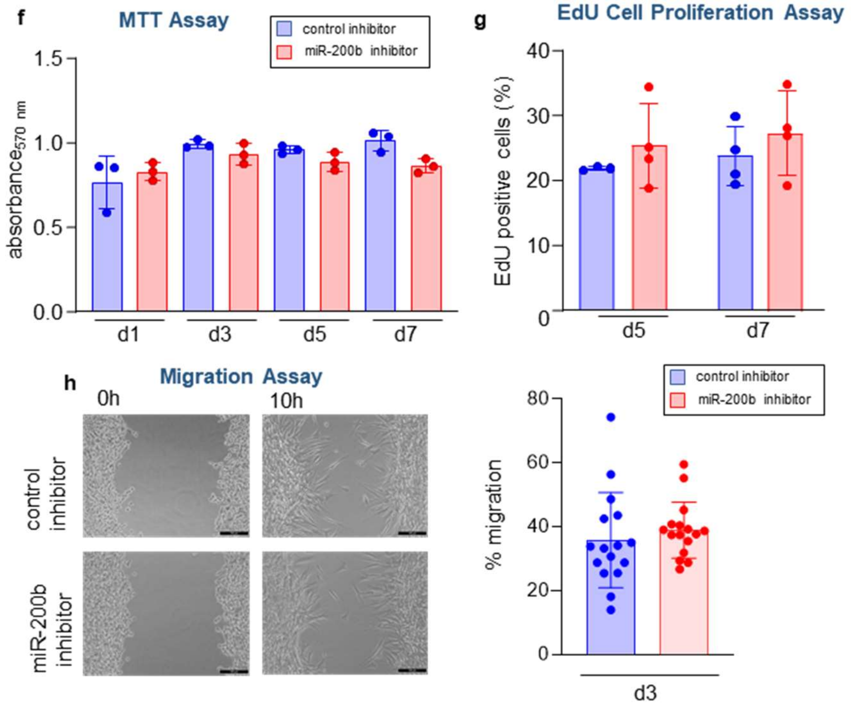

Supplementary Fig. 3 | Defining clusters 1 and other clusters in vasculogenic fibroblasts. a) Dot plot representing different plasma membrane proteins specific to clusters post-miR-200b inhibition. b) Flow sorting of cluster 1(B2M^{hi}ITGA2^{lo}) and rest other clusters (B2M^{lo}ITGA2^{hi}). c) miR-200b and FLI1 expression in B2M^{hi}ITGA2^{lo} cells and rest other clusters B2M^{lo}ITGA2^{hi}. HADF cells treated with control inhibitor were sorted for B2M^{lo}ITGA2^{hi} served as control. Results represent mean ± S.D. (n = 4-7). d) Scatter plots representing cell cycle scores for cluster 0 (left) and cluster 1 cells (right). e) Heatmaps representing expression of cell cycle markers for cluster 0 and cluster 1 over time. f-h) MTT assay (f) and EdU cell proliferation assay (g) and migration assay (h) of HADF transfected with either control inhibitor or miR-200b inhibitor at different time points post- transfection. No significance difference was observed between control inhibitor and miR-200b inhibitor at any time point. Data expressed as mean ± S.D. (f: n = 3; g: n = 3-4; h: n = 16), non-parametric student's t test.

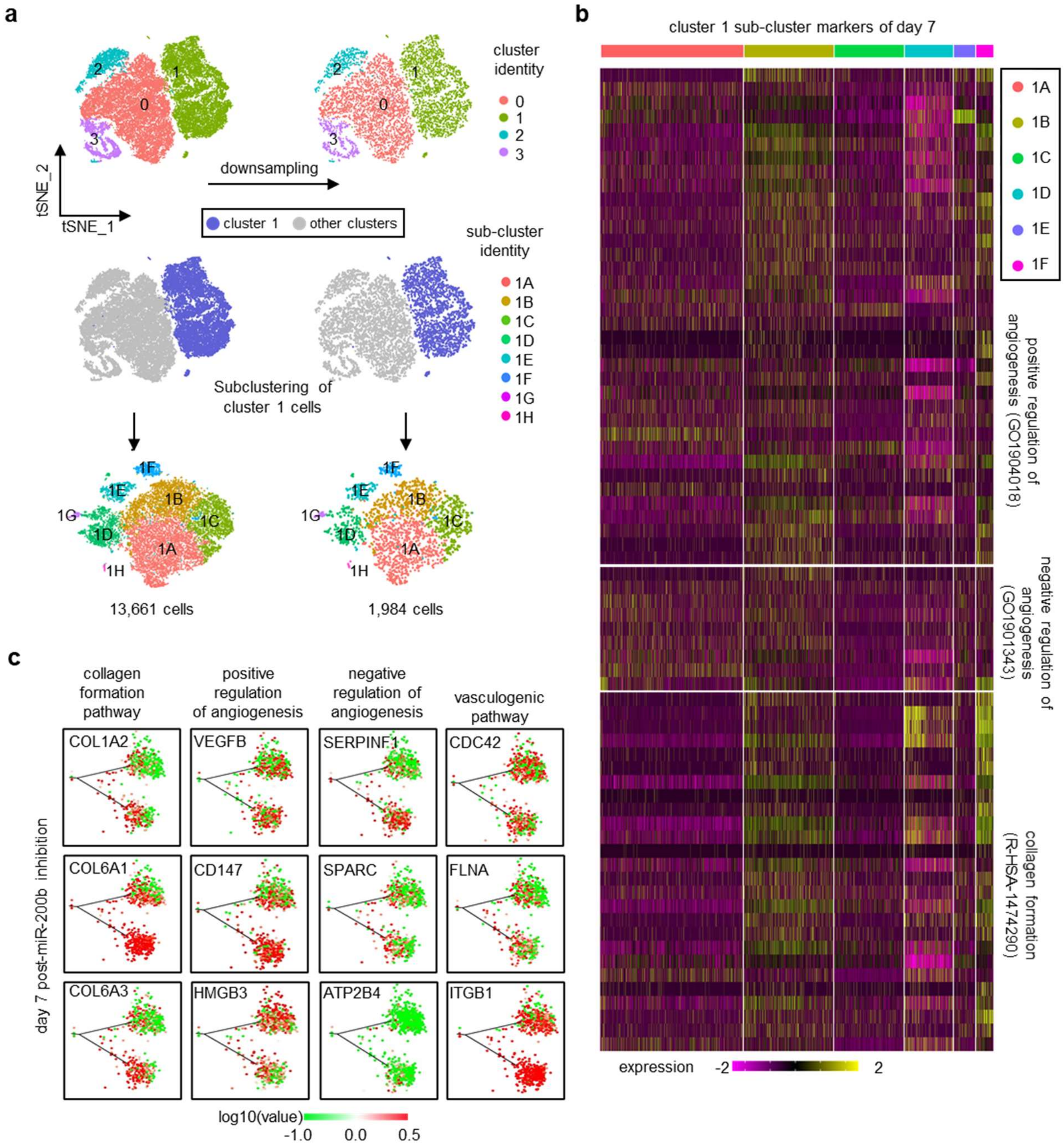

a
cluster identity
0
1
2
3
tSNE_2
tSNE_1
downsampling
cluster 1
other clusters
sub-cluster identity
1A
1B
1C
1D
1E
1F
1G
1H
Subclustering of cluster 1 cells
13,661 cells
1,984 cells
b
cluster 1 sub-cluster markers of day 7
1A
1B
1C
1D
1E
1F
positive regulation of angiogenesis (GO1904018)
negative regulation of angiogenesis (GO1901343)
collagen formation (R-HSA-1474290)
expression -2 2
c
collagen formation pathway
positive regulation of angiogenesis
negative regulation of angiogenesis
vasculogenic pathway
day 7 post-miR-200b inhibition
COL1A2
VEGFB
SERPINF1
CDC42
COL6A1
CD147
SPARC
FLNA
COL6A3
HMGB3
ATP2B4
ITGB1
log10(value)
-1.0
0.0
0.5

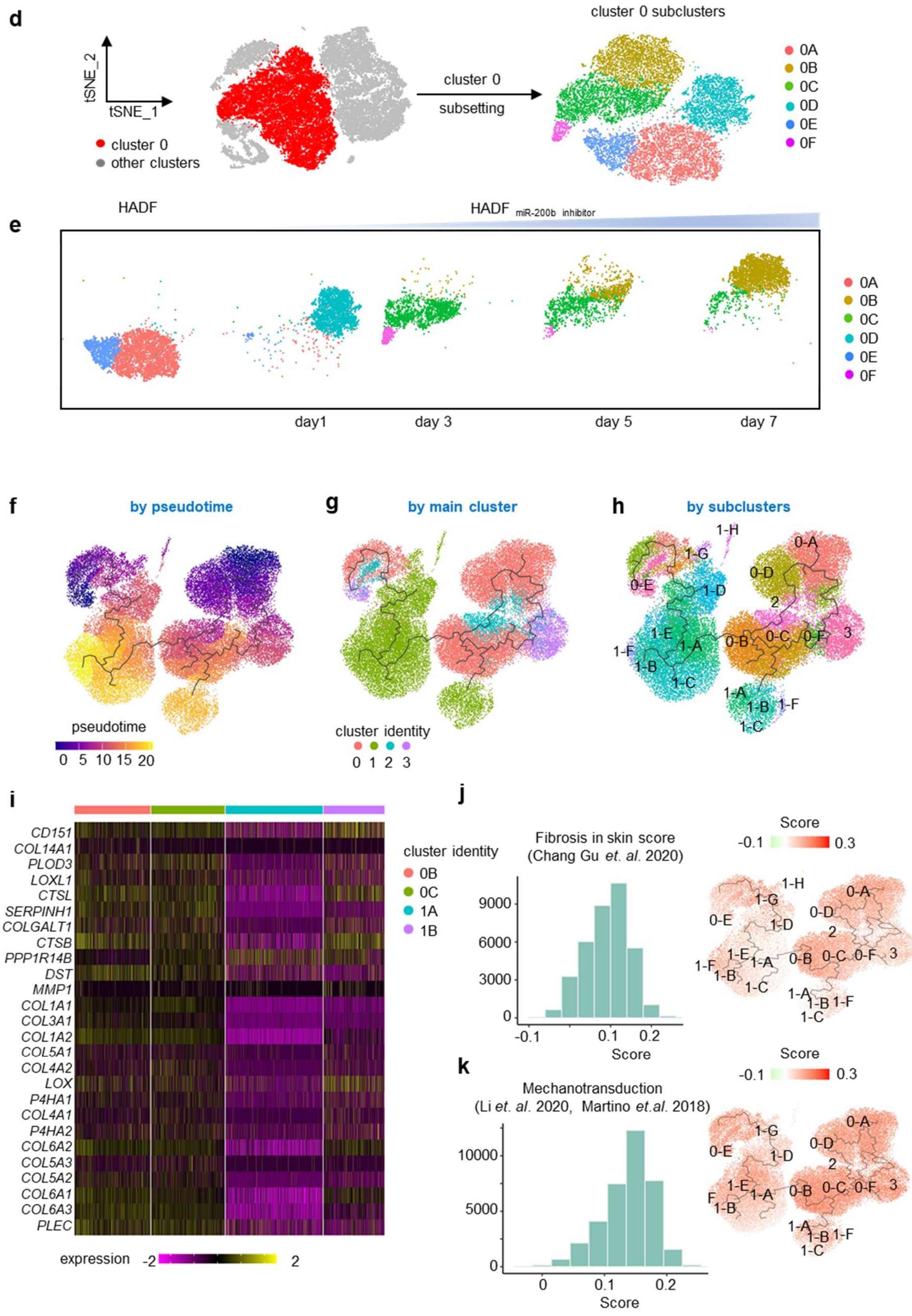
d
cluster 0 subclusters
tSNE_2
tSNE_1
cluster 0
other clusters
cluster 0
subsetting
0A
0B
0C
0D
0E
0F
e
HADF
HADF miR-200b inhibitor
day1
day 3
day 5
day 7
f
by pseudotime
pseudotime
0 5 10 15 20
g
by main cluster
cluster identity
0 1 2 3
h
by subclusters
i
cluster identity
0B
0C
1A
1B
CD151
COL14A1
PLOD3
LOXL1
CTSL
SERPINH1
COLGALT1
CTSB
PPP1R14B
DST
MMP1
COL1A1
COL3A1
COL1A2
COL5A1
COL4A2
LOX
P4HA1
COL4A1
P4HA2
COL6A2
COL5A3
COL5A2
COL6A1
COL6A3
PLEC
expression -2 2
j
Fibrosis in skin score
(Chang Gu et. al. 2020)
Score
-0.1 0.3
k
Mechanotransduction
(Li et. al. 2020, Martino et.al. 2018)
Score
-0.1 0.3

Supplementary Fig. 4 | More vasculature development genes were identified to be upregulated in subclusters 1B and 1D. Related to Figure 4. a) Down sampling: to perform the trajectory inference, a subset of cells (right side; 6000 cells) were used from the original 36,308 cells (left side). b) Heatmap showing gene expression dynamics during the fibroblast state transition process identified in cluster 1 subclusters and have role in vasculogenesis regulation (upper and middle panel). Lower panel includes collagen formation pathway genes identified to be downregulated over time (figure SII-C in data supplement). Columns represent individual cells. Rows represent genes. c) Expression level of selected genes from representing collagen formation pathway, positive and negative regulation of angiogenesis, and vasculogenic pathways over the terminal branches of the trajectory. Cells at the bottom of the trajectory were enriched more in collagen formation and anti-angiogenic genes compared to the cells present at the top of the trajectory. d) tSNE plot representing identification and subclustering of cluster. e) tSNE plot representing cluster 0 subclusters in each sample over time. f-h) UMAP plots representing the inferred trajectory using all cells colored by pseudotime (f), main clusters (g) and subclusters (h) respectively. Cluster 0B and 0C can represent the main precursors for cluster 1 population. i) Heatmap representing the relative expression of genes which were enriched in the collagen formation pathway in clusters 0B, 0C, 1A and 1B. Columns represent individual cells. Rows represent genes. j-k) Histograms (left) and UMAP plot representing inferred trajectory (right) representing distribution of calculated scores based on skin fibrosis (j) and mechanotransduction (k) respectively. Cells were colored by their calculated scores.

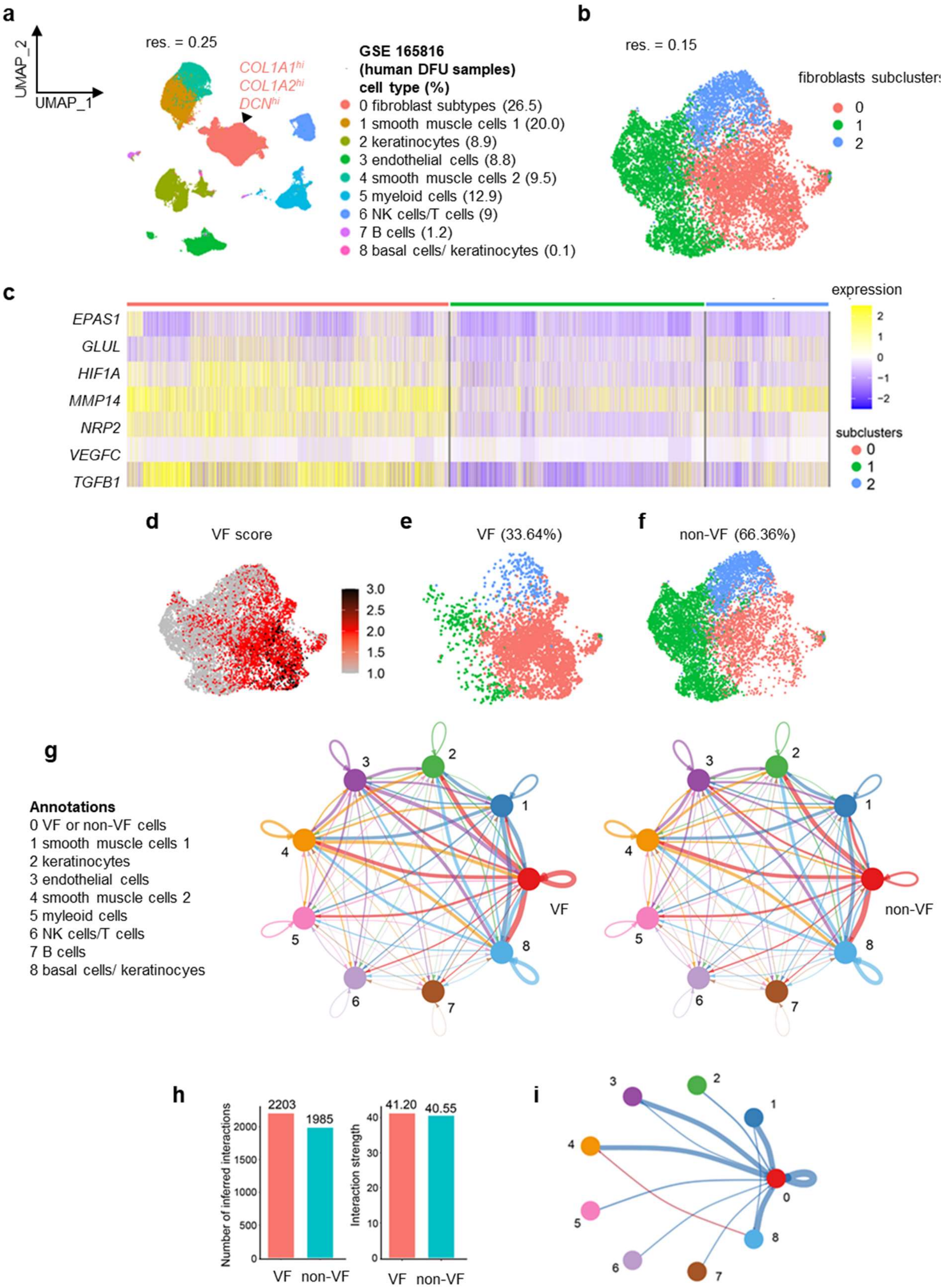

a
UMAP_2
UMAP_1
res. = 0.25
COL1A1hi
COL1A2hi
DCNhi
GSE 165816
(human DFU samples)
cell type (%)
0 fibroblast subtypes (26.5)
1 smooth muscle cells 1 (20.0)
2 keratinocytes (8.9)
3 endothelial cells (8.8)
4 smooth muscle cells 2 (9.5)
5 myeloid cells (12.9)
6 NK cells/T cells (9)
7 B cells (1.2)
8 basal cells/ keratinocytes (0.1)
b
res. = 0.15
fibroblasts subclusters
0
1
2
c
EPAS1
GLUL
HIF1A
MMP14
NRP2
VEGFC
TGFB1
expression
subclusters
d
VF score
e
VF (33.64%)
f
non-VF (66.36%)
g
Annotations
0 VF or non-VF cells
1 smooth muscle cells 1
2 keratinocytes
3 endothelial cells
4 smooth muscle cells 2
5 myleoid cells
6 NK cells/T cells
7 B cells
8 basal cells/ keratinocytes
VF
non-VF
h
Number of inferred interactions
2203
1985
Interaction strength
41.20
40.55
VF
non-VF
i

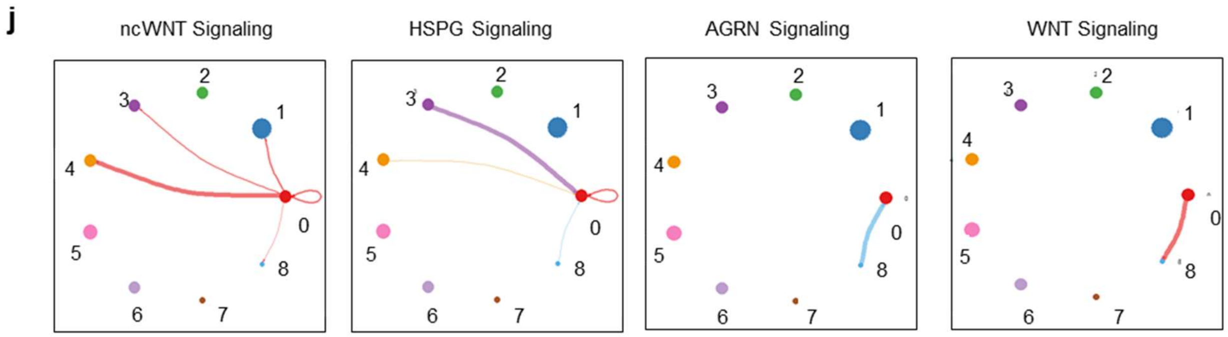

Supplementary Fig. 5 | Characterization of VF population in DFU samples. For this work, single-cell RNA seq data of 14 human DFU samples (n=11), were collected from Gene Expression Omnibus (GEO) with accession number GSE165816. All the processed scRNA-seq samples were quality filtered, normalized using 'SCT-transformation' and stored as Seurat objects in R. Further, these Seurat objects were integrated, and executed for clustering analysis (with resolution 0.25) using Seurat (a) Resulting cluster of cells were illustrated in UMAP plot, showing the 9 (0-8) clusters with distinct cluster identities. Cell type annotations based on cluster specific markers (from PanglaoDb) along with their abundances were documented. b) Three sub-clusters, identified in the re-clustering analysis of the fibroblast cluster using Seurat (at resolution = 0.10), were illustrated as UMAP plot. (c) Heatmap showing the relative expression of VF signature genes across fibroblast subclusters which were significantly elevated (Student's t-test, p-value ≤ 0.01, log2 fold change > 0.30) in sub-cluster 0 compared to other subclusters. d) Distribution of cells representing the VF score (computed based on significant VF genes) was illustrated in UMAP plot. (e-f) Based on VF score, the fibroblast subclusters were further classified into VF and non-VF cells as shown in respective UMAP plots. g) CellChat derived communication network of VF included connectome and non-VF included connectome were shown as communication network. h) Bar graph showing the total number of interactions and weighted interaction strength of VFS and non-VF included connectome using CellChat package in R. (i) The inferred cell-cell communication networks were compared and differential connectome

of VF over non-VF was illustrated in network plot. j) Top four signaling pathways (ncWNT, HSPG, AGRN and WNT) significantly enriched only in VF (not present in non-VF) included connectome was identified.

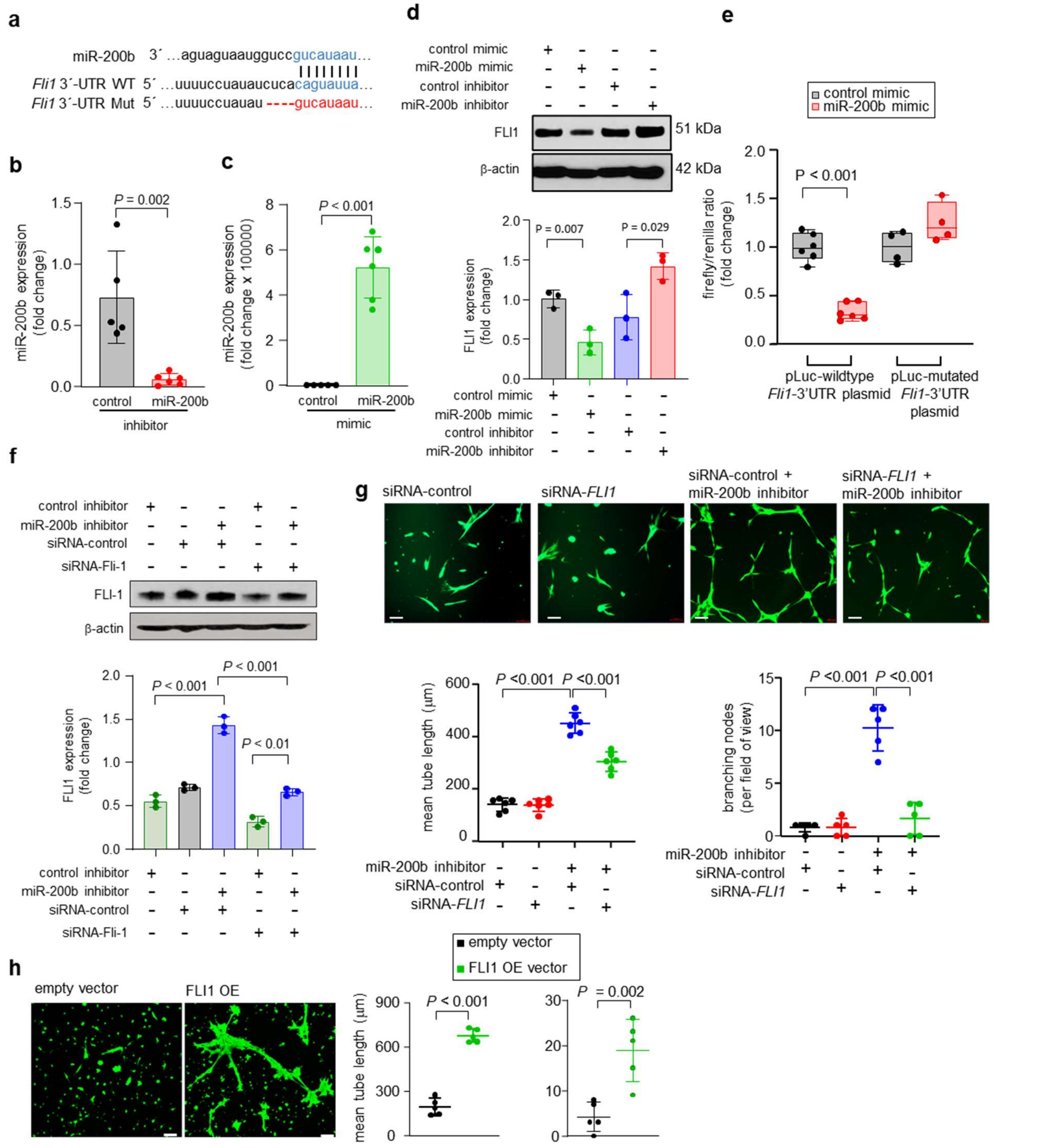

Supplementary Fig. 6 | miR-200b inhibition in fibroblasts upregulates FLI1 to promote the vasculogenic state. a) Predicted binding site in FLI1 3′-UTR for hsa-miR-200b as predicted by in silico studies. The mutated seed sequence of FLI1 3′-UTR was shown in red. b) (b-c) miR-200b expression in HADF after delivery of either miR-200b inhibitor (b) or mimic (c). Results represent mean ± S.D. (n=6). d) Western blot analysis (top) and densitometric quantification (bottom) of FLI1 in HADFs after transfecting with either miR-200b mimic or inhibitor. b-actin serves as loading control. Data expressed as mean ± S.D. (n=3). e) miR target reporter luciferase assay after delivery of miR-200b mimic in HADF transfected with either wild type FLI1 3′-UTR plasmid or mutated FLI1 3′-UTR plasmids. Results were normalized with renilla luciferase activity. Data are mean ± S.D. The line represents the mean and the whiskers represent the standard deviation. (n = 6,4). f) Western blot analysis (top) and densitometric quantification (bottom) of FLI1 in HADFs after transfecting with either miR-200b mimic or inhibitor in presence or absence of sh-FLI1 RNA. b-actin serves as loading control. Data expressed as mean ± S.D. (n=3). g) Matrigel tube formation (top) at day 7 post-in-vitro TNT of miR-200b inhibitor transfected HADF in presence or absence of control or *FLI1* siRNA. Scale, 100µm. Quantification of tube length (µm) and branching nodes of each group was plotted graphically (bottom). Data expressed as mean ± S.D.(n=5-6). h) Matrigel™ tube formation (left) at day 7 post-*in vitro* TNT of *FLI1* overexpression plasmid. Scale, 100µm. Quantification of tube length (µm) and branching nodes of each group was plotted graphically (bottom). Data expressed as mean ± S.D.(n=5). Data in b, c, d and h were analyzed by two-tailed unpaired Student's t test. Data in f and g were analyzed by one-way analysis of variance with the *post-hoc* Bonferroni multiple comparison test.

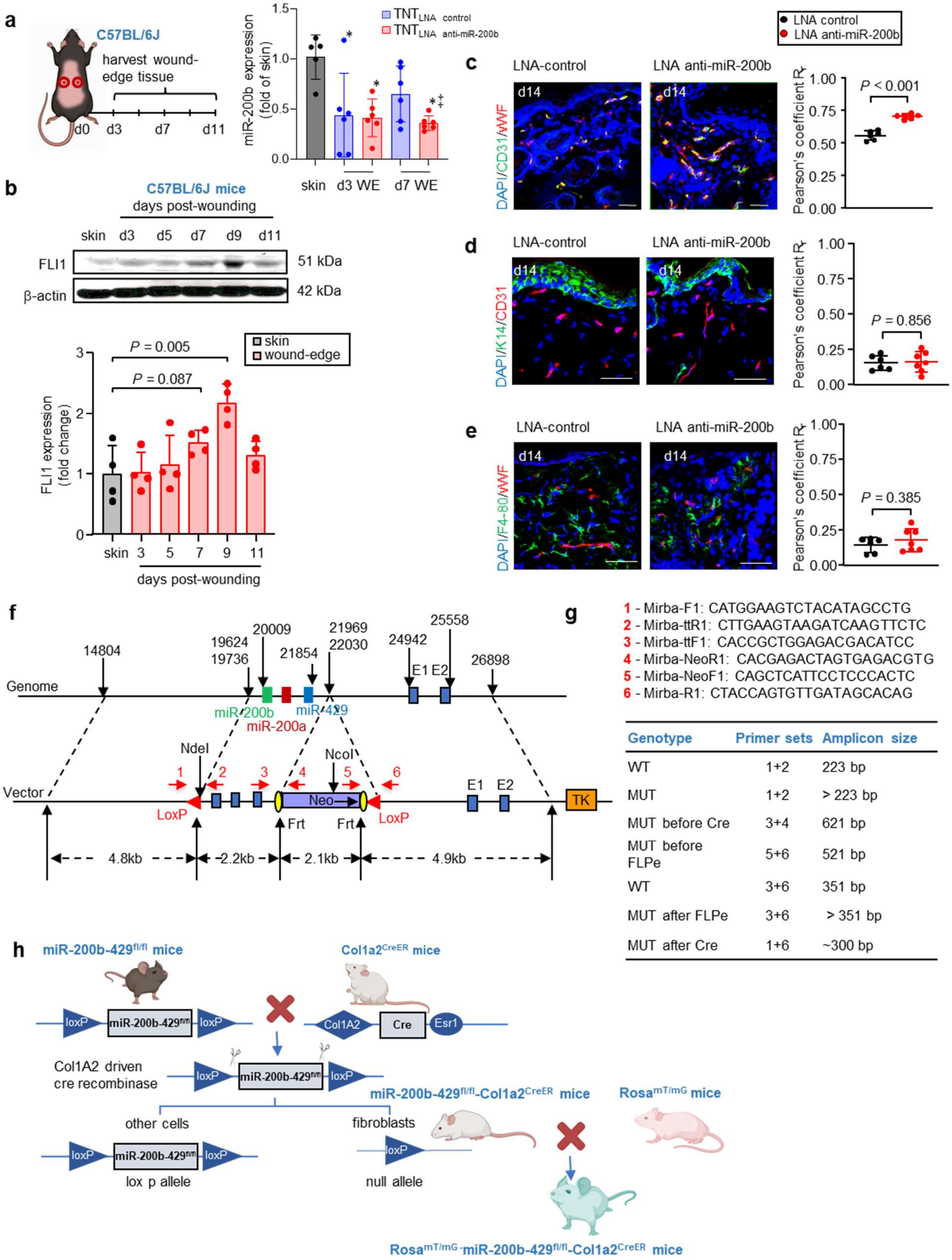

Genotype	Primer sets	Amplicon size
WT	1+2	223 bp
MUT	1+2	> 223 bp
MUT before Cre	3+4	621 bp
MUT before FLPe	5+6	521 bp
WT	3+6	351 bp
MUT after FLPe	3+6	> 351 bp
MUT after Cre	1+6	~300 bp

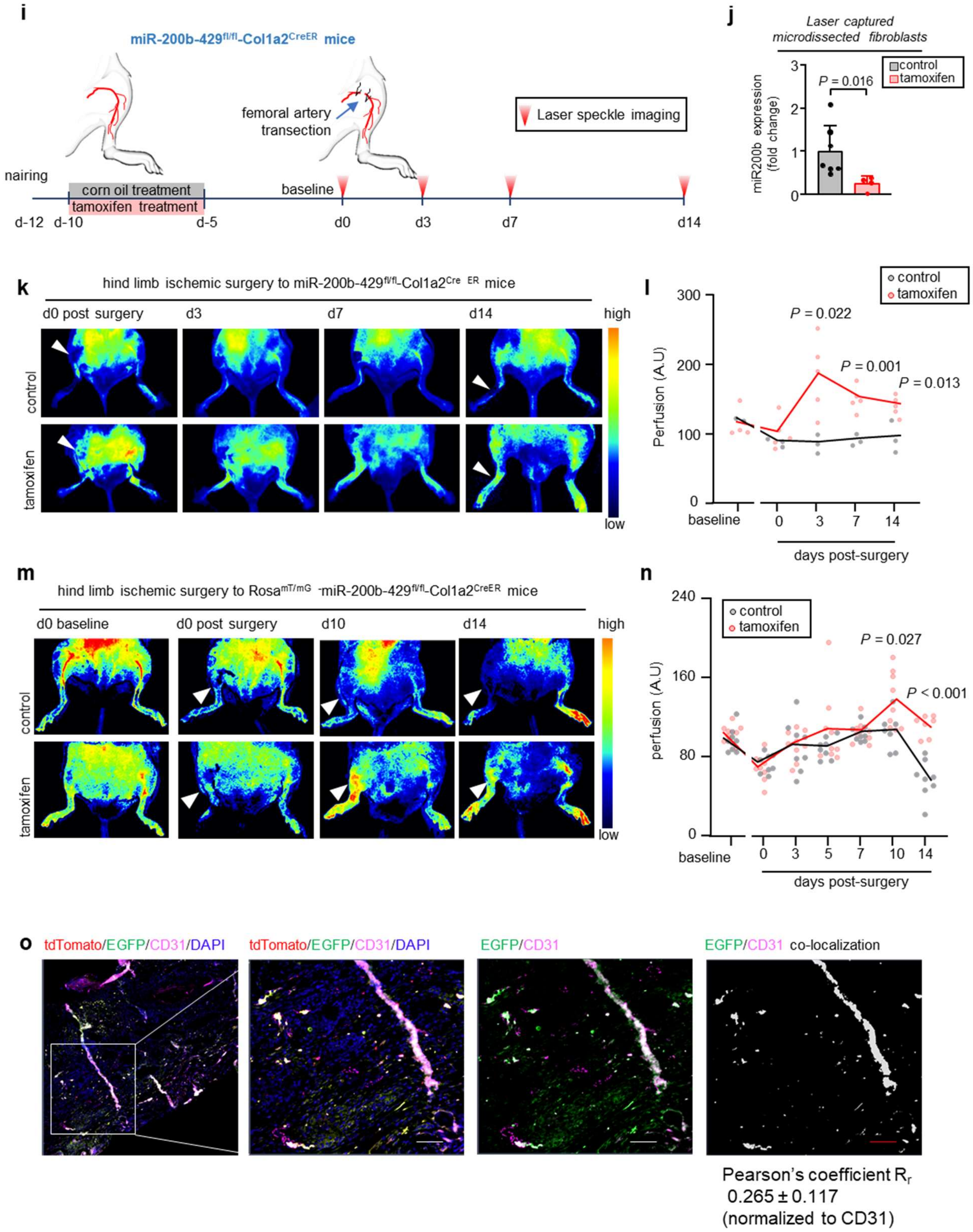

i
miR-200b-429fl/fl-Col1a2CreER mice
femoral artery transection
Laser speckle imaging
nairing
corn oil treatment
tamoxifen treatment
baseline
d-12 d-10 d-5 d0 d3 d7 d14
j
Laser captured microdissected fibroblasts
miR200b expression (fold change)
P = 0.016
control
tamoxifen
k
hind limb ischemic surgery to miR-200b-429fl/fl-Col1a2Cre ER mice
d0 post surgery
d3
d7
d14
high
low
control
tamoxifen
l
Perfusion (A.U)
P = 0.022
P = 0.001
P = 0.013
baseline
days post-surgery
m
hind limb ischemic surgery to RosamT/mG -miR-200b-429fl/fl-Col1a2CreER mice
d0 baseline
d0 post surgery
d10
d14
n
perfusion (A.U)
P = 0.027
P < 0.001
o
tdTomato/EGFP/CD31/DAPI
EGFP/CD31
EGFP/CD31 co-localization
Pearson's coefficient Rr
0.265 ± 0.117
(normalized to CD31)

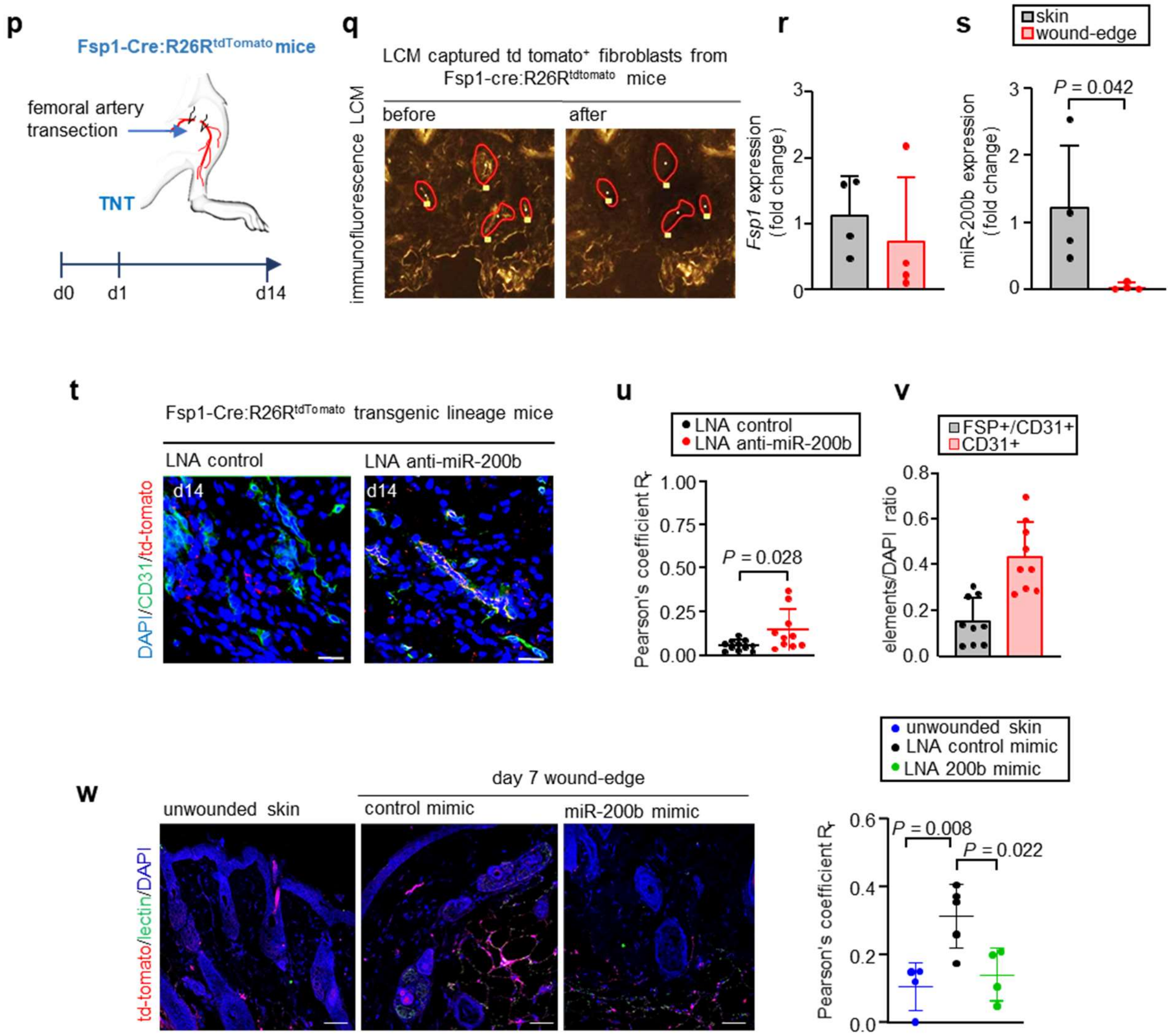

Supplementary Fig. 7 | Increased wound angiogenesis by anti-miR200b-LNA delivery. Related to Figure 6. a) Schematic diagram showing collection of wound-edge (WE) tissue from and miR-200b expression in skin and WE tissue of C57BL/6 mice treated with either LNA control and LNA anti-miR 200b inhibitor at day 3 and day 7. Data expressed as mean ± S.D. (n = 5-6). * P< 0.05 vs skin; ‡ P < 0.05 vs day 7 $TNT_{LNA\ control}$. b) FLI1 expression and densitometric quantification (bottom) of FLI1 post-wounding days in wound-edge tissue of C57BL/6 mice. β-actin serves as a loading control. Results represent mean ± S.D. (n = 4). c) Immunofluorescence

confocal images of hind-limb ischemic skin section (left) and colocalization quantitation (right) of CD31(green) and vWF (red) staining of anti-miR200b LNA treated C57BL/6 mice vs control LNA treated mice. Results represent mean ± S.D.(n = 6). Scale, 50μm. d) Immunofluorescence image of hind-limb ischemic skin section (left) and colocalization quantitation (right) of K14 (green) and red CD31 (red) staining of anti-miR200b LNA treated C57BL/6 mice vs control LNA treated mice. Results represent mean ± S.D.(n = 6). Scale, 50μm. e) Immunofluorescence confocal image of hind-limb ischemic skin section (left) and colocalization quantitation (right) of F4/80 (green) macrophage marker and vWF (red) staining of anti-miR200b LNA treated C57BL/6 mice vs control LNA treated mice. Results represent mean ± S.D. (n = 6-7). Scale, 50μm. f) Vector design for the generation of miR-200b-429$^{fl/fl}$Col1a2^{creER} mice. g) Primers and amplicon size used in the genotyping of miR-200b-429$^{fl/fl}$Col1a2^{creER} mice. h) Schematic diagram showing the breeding plan of miR-200b-429$^{fl/fl}$Col1a2^{creER} and ROSA$^{mT/mG}$ miR-200b-429$^{fl/fl}$Col1a2^{creER} mice. i) Schematic diagram of hind-limb (HL) surgery experiment in miR-200b-429$^{fl/fl}$Col1a2^{creER} mice. j) The abundance of miR-200b in fibroblasts post-tamoxifen application was shown graphically. Results represent mean ± S.D. (n = 7,4). k-l) Representative laser speckle perfusion images (k) and quantification (l) of corn-oil treated (top) and tamoxifen treated (bottom) in miR-200b-429$^{fl/fl}$-Col1a2^{CreER} mice at different time points post-surgery. The lines represent the mean, and the dots represent the individual value. (n = 3,5). m-n) Representative laser speckle perfusion images (k) and quantification (l) of corn-oil treated (top) and tamoxifen treated (bottom) in ROSA$^{mT/mG}$ miR-200b-429$^{fl/fl}$Col1a2^{creER} mice at different time points post-surgery. The lines represent the mean, and the dots represent the individual value. (n = 8). o) Colocalization of CD31 and EGFP was determined by Pearson correlation (r). (n = 5) p) Schematic diagram of hind-limb (HL) surgery experiment in FSP-1 cre mice. q-s) FSP1 and miR-200b expression in dermal fibroblast-rich skin

and wound-edge tissue of FSP1-cre mice collected by Laser Captured Microdissection (LCM). Results represent mean ± S.D. (n = 4). t) Immunofluorescence confocal image of day 14 wound-edge tissue in FSP1-cre mice stained for CD31. Scale, 50 µm. u) Colocalization of CD31 and td-tomato was determined by Pearson correlation (r). Data expressed as mean ± S.D. (n = 10). v) The CD31 and td-tomato colocalized vascular elements were quantified in FSP1-cre mice and plotted graphically. Data expressed as mean ± S.D. (n = 9) w) Colocalization of CD31 and td-tomato in unwounded skin and day 7 wound-edge transfected with control or miR-200b mimic was determined by Pearson correlation (r). Data expressed as mean ± S.D. (n = 4-5). Fig. S7a, S7h, S7i and S7p were created with BioRender.com. Data in a, c, d, e, j, l, n, r, s, u and v were analyzed by two-tailed unpaired Student's t test. Data in b and c were analyzed by one-way analysis of variance with the *post-hoc* Bonferroni multiple comparison test.

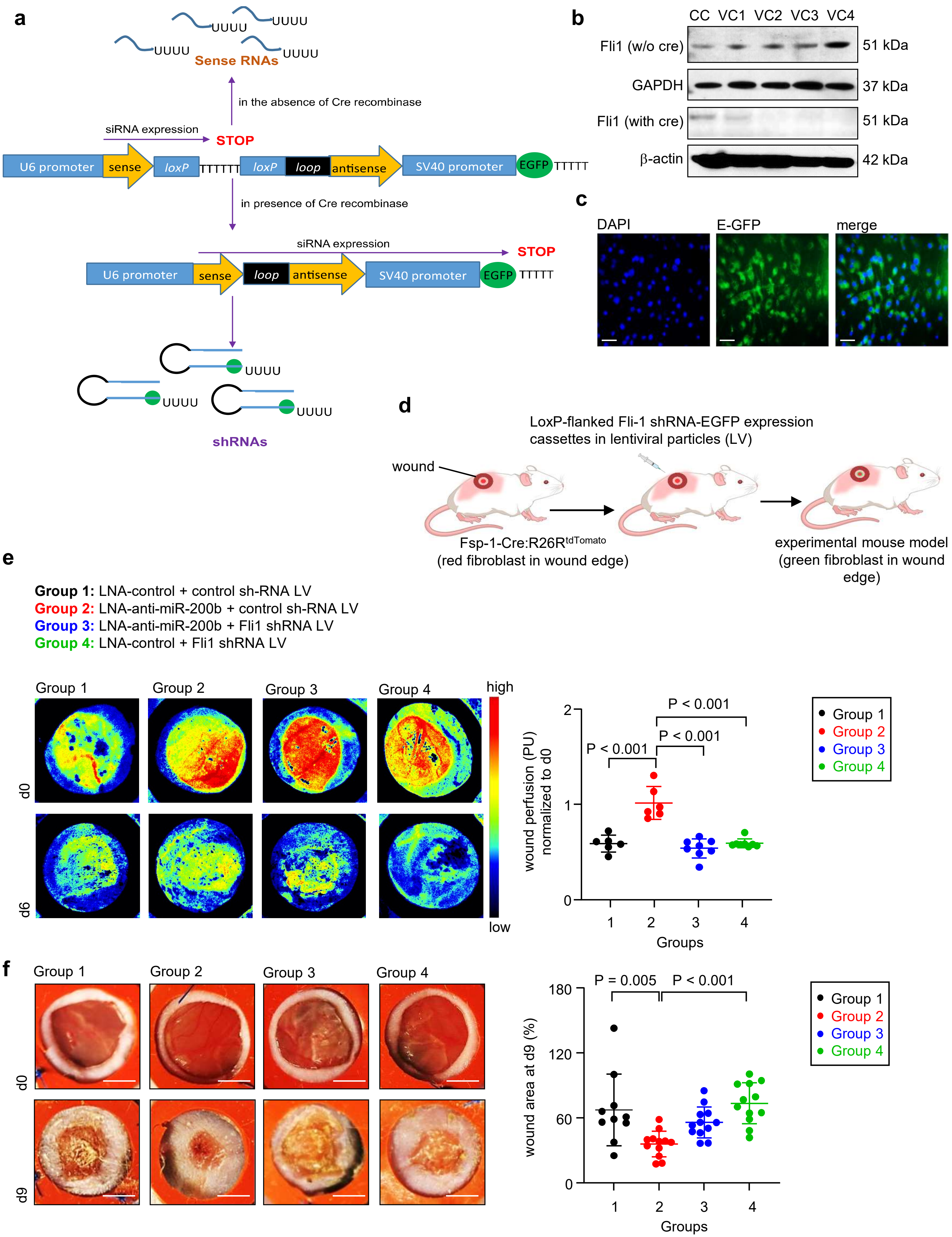

a
UUUU
UUUU
UUUU
UUUU
Sense RNAs
in the absence of Cre recombinase
siRNA expression
STOP
U6 promoter
sense
loxP
TTTTTT
loxP
loop
antisense
SV40 promoter
EGFP
TTTTT
in presence of Cre recombinase
siRNA expression
STOP
U6 promoter
sense
loop
antisense
SV40 promoter
EGFP
TTTTT
UUUU
UUUU
UUUU
shRNAs
b
CC VC1 VC2 VC3 VC4
Fli1 (w/o cre)
51 kDa
GAPDH
37 kDa
Fli1 (with cre)
51 kDa
β-actin
42 kDa
c
DAPI
E-GFP
merge
d
LoxP-flanked Fli-1 shRNA-EGFP expression cassettes in lentiviral particles (LV)
wound
Fsp-1-Cre:R26R^tdTomato
(red fibroblast in wound edge)
experimental mouse model (green fibroblast in wound edge)
e
Group 1: LNA-control + control sh-RNA LV
Group 2: LNA-anti-miR-200b + control sh-RNA LV
Group 3: LNA-anti-miR-200b + Fli1 shRNA LV
Group 4: LNA-control + Fli1 shRNA LV
Group 1
Group 2
Group 3
Group 4
d0
d6
high
low
wound perfusion (PU) normalized to d0
P < 0.001
P < 0.001
P < 0.001
0
1
2
1
2
3
4
Groups
Group 1
Group 2
Group 3
Group 4
f
Group 1
Group 2
Group 3
Group 4
d0
d9
wound area at d9 (%)
P = 0.005
P < 0.001
0
60
120
180
1
2
3
4
Groups
Group 1
Group 2
Group 3
Group 4

Supplementary Fig. 8 | FLI 1 involvement for direct *in vivo* reprogramming of dermal fibroblasts into vasculogenic fibroblasts. a) Schematic diagram showing Cre loxP regulated fibroblast specific Fli 1 shRNA expression. b) Four different FLI1 shRNA vectors were validated in dermal fibroblasts without and with Cre mediated deletion of the STOP cassette. Western blot analysis showing FLI1 protein expression in cells co-transfected with cre recombinase vector (pCSCre2) and each of four different FLI1 shRNA expression cassettes. CC, control shRNA construct and VC, lentiviral FLI1 shRNA constructs. c) Representative image showing E-GFP fluorescence in HADF-Cre cells transfected with LoxP flanked FLI1 shRNA expression cassettes. Scale, 100µm. d) Diagrammatic view of study design for targeted knocking down of FLI 1 at wound edge fibroblasts. e) Representative cutaneous blood perfusion images and quantification at day 6 wound-edge tissue of Fsp^{cre}tdTomato mice treated with either LNA-control or LNA-anti-miR-200b inhibitor in absence or presence of control or Fli1 sh-RNA lentiviral particles. Scale, 2mm. (n = 6) f) Digital photograph of Fsp^{cre}tdTomato mice wound-edge tissue at day 9 treated with either LNA-control or LNA-anti-miR-200b inhibitor in absence or presence of control or Fli1 sh-RNA lentiviral particles. Scale, 2mm. (n = 9-12). Fig. S8d created with BioRender.com. Data in e and f were analyzed by one-way analysis of variance with the *post-hoc* Bonferroni multiple comparison test.

a

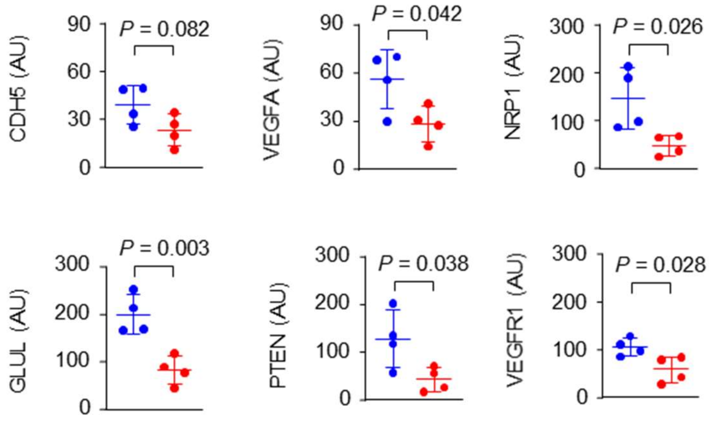

b

Table 1: Demographics and clinical characteristics of chronic wound patients

Characteristics	Non-Diabetic (n=10)	Diabetic (n=12)
**Age**§	59 (48-78)	65 (48-79)
**Race**†		
White	6	6
African American	1	3
Not reported*	3	3
**Sex**		
Male	6	8
Female	1	2
Not reported*	3	2
**HbA1c**§	5.35 (5.2 - 5.5)	9.2 (5.4 - 13.1)
**Wound Location**		
Foot	2	10
Ischium	6	
Sacrum	1	
Not reported*	1	2

§ Data reported as median.

† Race was reported by patients

* Surgically discarded and de-identified samples

Supplementary Fig. 9 | Quantification of imaging mass cytometry data and demographics of human subjects. a) The Imaging mass cytometry data exported from the MCD viewers were quantified in ImageJ software and plotted graphically as mean ± S.D. (n=4) b) Table showing the age, race, sex, HbA1c and wound locations of human wound samples used in the study. Data in a, were analyzed by two-tailed unpaired Student's t test.

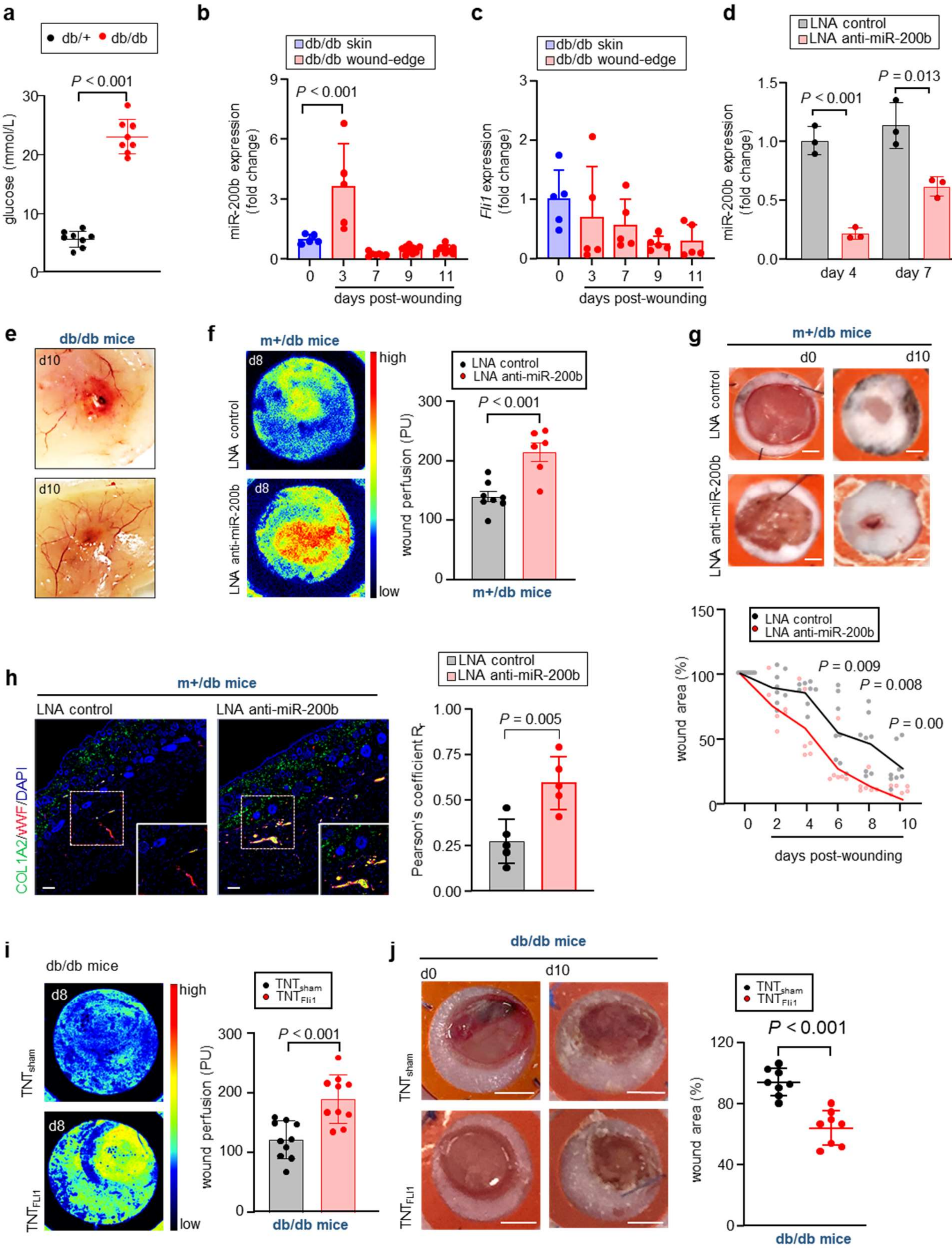
a
db/+ db/db
P < 0.001
glucose (mmol/L)
b
db/db skin
db/db wound-edge
P < 0.001
miR-200b expression (fold change)
days post-wounding
c
db/db skin
db/db wound-edge
Fli1 expression (fold change)
days post-wounding
d
LNA control
LNA anti-miR-200b
P < 0.001
P = 0.013
miR-200b expression (fold change)
day 4
day 7
e
db/db mice
d10
f
m+/db mice
d8
high
low
LNA control
LNA anti-miR-200b
P < 0.001
wound perfusion (PU)
m+/db mice
g
m+/db mice
d0
d10
LNA control
LNA anti-miR-200b
P = 0.009
P = 0.008
P = 0.00
wound area (%)
days post-wounding
h
m+/db mice
LNA control
LNA anti-miR-200b
COL1A2/vWF/DAPI
P = 0.005
Pearson's coefficient Rr
i
db/db mice
d8
high
low
TNTsham
TNTFLI1
P < 0.001
wound perfusion (PU)
db/db mice
j
db/db mice
d0
d10
TNTsham
TNTFli1
P < 0.001
wound area (%)
db/db mice

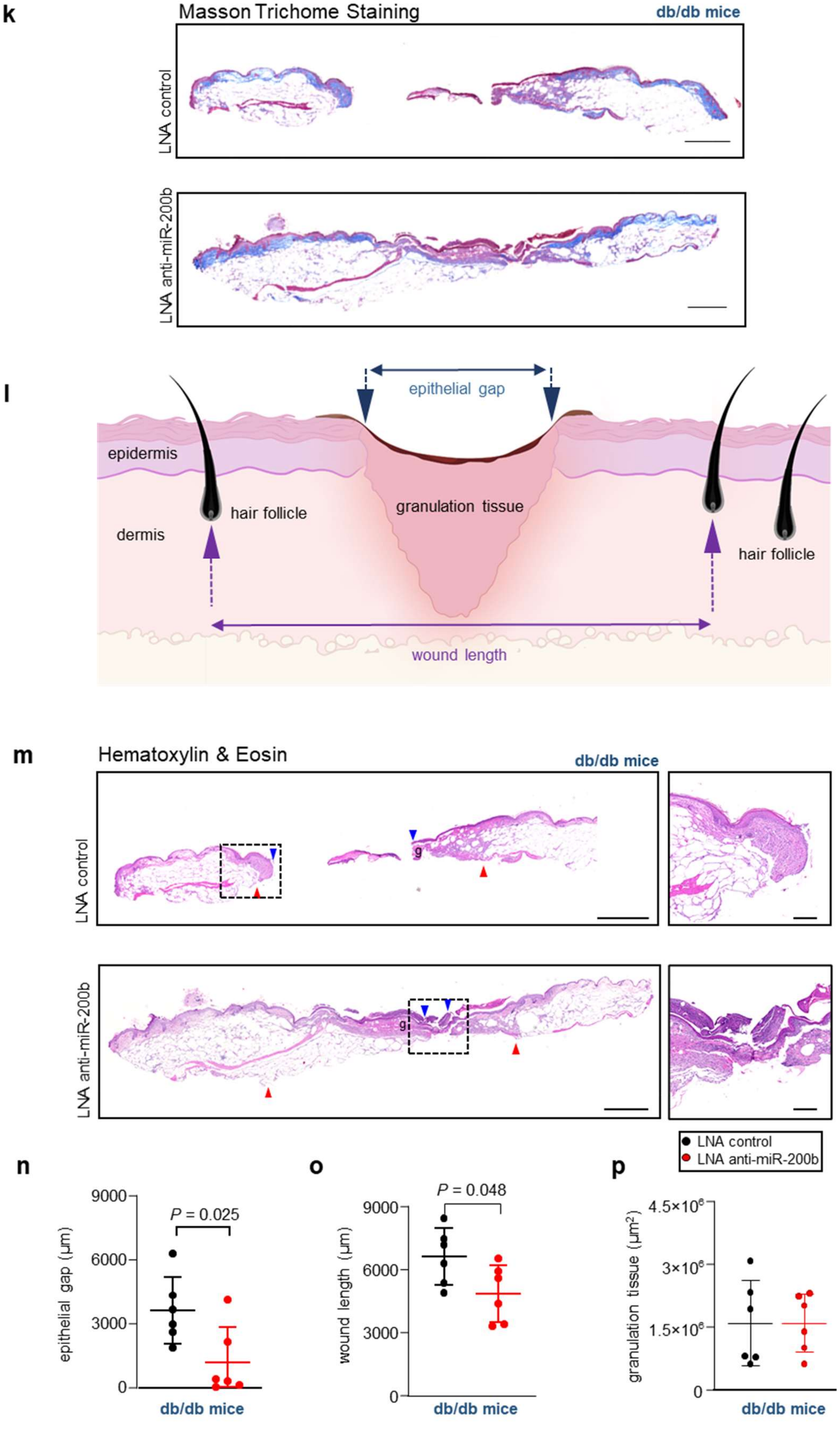
k
Masson Trichome Staining
db/db mice
LNA control
LNA anti-miR-200b
l
epithelial gap
epidermis
hair follicle
granulation tissue
dermis
hair follicle
wound length
m
Hematoxylin & Eosin
db/db mice
LNA control
g
LNA anti-miR-200b
g
LNA control
LNA anti-miR-200b
n
epithelial gap (µm)
P = 0.025
9000
6000
3000
0
db/db mice
o
wound length (µm)
P = 0.048
9000
6000
3000
0
db/db mice
p
granulation tissue (µm²)
4.5×10⁶
3×10⁶
1.5×10⁶
0
db/db mice

Supplementary Fig. 10 | Administration of anti-miR-200b-LNA attenuates impairment of wound healing in db/db mice. a) Blood glucose level in non-diabeic (db/+) and diabetic (db/db) mice. (n = 8). b-c) miR-200b (b) and Fli1 expression (c) in skin and WE tissue of db/db mice. (n = 5-9). d) miR-200b expression at d4 and d7 WE tissue of db/db mice treated with either $LNA_{control}$ or $LNA_{anti\text{-}miR\text{-}200b}$ inhibitor. (n = 3). e) Wound vasculature of $LNA_{control}$ or $LNA_{anti\text{-}miR\text{-}200b}$ inhibitor treated db/db skin at d10. f) Cutaneous blood perfusion images and quantification at d8 WE tissue of db/db mice treated with either $LNA_{control}$ or $LNA_{anti\text{-}miR\text{-}200b}$ inhibitor. (n= 6) g) Digital photograph of m+/db mice WE tissue at d0 and d10 treated with either $LNA_{control}$ or $LNA_{anti\text{-}miR\text{-}200b}$ inhibitor. Scale, 5 mm. Digital planimetry of the wound area was quantified and plotted graphically. (n=6). h) Immunofluorescence confocal image of d10 WE tissue in m+/db mice stained for COL1A2 and vWF. Colocalization of COL1A2 and vWF was determined by Pearson correlation (r). (n = 5). i) Representative cutaneous blood perfusion images and quantification at d8 WE tissue of db/db mice treated with either control or Fli1 over expressing plasmids. (n= 6) j) Digital photograph of db/db mice WE tissue at day 0 and day 10 treated with either control or Fli1 over expressing plasmids. Scale, 5 mm. (n=10) Digital planimetry of the wound area was quantified and plotted graphically. (n=6-8). k) Masson trichrome staining of day 10 wound-edge tissue in db/db mice treated with either $LNA_{control}$ or $LNA_{anti\text{-}miR\text{-}200b}$ inhibitor. Scale, 1000 µm. (n = 6). l) Schematic diagram showing the wound-section showing the regions used for analytical histology. d, dermis; e, epidermis, g, granulation tissue; he, hyperproliferative epithelium; hf, hair follicle. m) Representative H&E staining of d10 wounds of db/db mice treated with either $LNA_{control}$ or $LNA_{anti\text{-}miR\text{-}200b}$ inhibitor. Scale, 1000 µm. (n = 6). n-p) Morphometric analysis showing epithelial gap (n), wound-length (o) and granulation tissue area (p) in db/db mice treated with either $LNA_{control}$ or $LNA_{anti\text{-}miR\text{-}200b}$ inhibitor. (n=6) All data were shown as mean ± S.D. Fig.

S10l created with BioRender.com Data in a, d, f, g, h, i, j, n, o and p were analyzed by two-tailed unpaired Student's t test. Data in b and c were analyzed by one-way analysis of variance with the *post-hoc* Bonferroni multiple comparison test.

Supplementary Table 1

List of primers used in this study

**Primer Name**	**Primer Sequence**
H_FLI1_F	5’-GGGCTGGGCTGCAGACTTGG-3’
H_FLI1_R	5’-GGGGCTGCCCGTAGTCAGGA-3’
H_S100A4_F	5’-TCTTGGTTTGATCCTGACTGCT-3’
H_S100A4_R	5’-TCGTTGTCCCTGTTGCTGTC-3’
H_VEGFB_F	5’-AGCACCAAGTCCGGATG -3’
H_VEGFB_R	5’-GTCTGGCTTCACAGCACTG -3’
H_COL1A2_F	5’-GAGGGCAACAGCAGGTTCACTTA-3’
H_COL1A2_R	5’-TCAGCACCACCGATGTCCAA-3’
H_18S_F	5’-GTAACCCGTTGAACCCCATT-3’
H_18S_R	5’-CCATCCAATCGGTAGTAGCG-3’
H_VWF_F	5’-CTGGCAGCTGTTCTTATGTCCTATT-3’
H_VWF_R	5’-CTCATGCATGATGGCACCATAA-3’
H_CD31_F	5’-AAATGCTCTCCCAGCCCAGGAT-3’
H_CD31_R	5’-GCAACACACTGGTATTCGACGTCTT-3’

Supplementary Table 2

List of vasculogenic genes in different subcluster of cluster 1 (Figure 4B) selected for RT2 PCR study

	**Subclusters**					
**Gene_ name**	**A**	**B**	**C**	**D**	**E**	**Vasculature development**
JUNB	-0.42	-0.41	-0.44	0.41	-0.35	Yes
ANXA2	0.55	0.39	-0.08	0.32	0.37	Yes
MAP2K1	-0.17	-0.12	-0.29	0.10	-0.06	Yes
GLUL	-0.32	-0.21	-0.97	0.25	-0.53	Yes
ACTA2	0.03	0.04	-0.23	0.23	-0.12	Yes
ADM	-0.74	-0.68	-0.78	0.17	-0.64	Yes
CDC42	-0.25	-0.10	-0.56	0.06		Yes
EFEMP2		0.42	0.31	0.22		Yes
MMP14	-0.74	-0.14	-0.57	0.09	-0.67	Yes
TNFRSF12A	-0.96	-0.63	-1.07	0.14	-0.49	Yes
THY1	-0.07		0.05	0.33	-0.26	Yes
NAXE	0.10	0.02	-0.10	0.26		Yes
FOSL1	-0.55	-0.49	-0.60	0.08	-0.48	Yes
TMEM204	-0.05	0.01	-0.05	0.28	-0.10	Yes
CLIC4		0.39	-0.17	0.28		Yes
CIB1	0.20	0.30	-0.25	0.29		Yes
HIF1A	-1.23	-0.69	-1.62	0.16	-0.98	Yes
VEZF1	-0.19	-0.19	-0.27	0.08	-0.14	Yes
LAMA4	-0.66		-0.62	0.25	-0.61	Yes
AKT1	0.15	0.07	-0.02	0.11		Yes
EPAS1	0.03	0.04	0.01	0.05	-0.04	Yes
HAND2	-0.14	-0.10	-0.21	0.15	-0.02	Yes
TGFBI	-0.06			0.10		Yes
AAMP	-0.17	-0.07	-0.37	0.09	-0.05	Yes
VEGFA	-0.20	-0.19	-0.23	0.03	-0.15	Yes
VEGFB	0.36	0.27	0.59	0.17		Yes

Supplementary Table 3

Gene sets used to calculate fibrosis and mechanotransduction score in cells subjected to miR-200b inhibition	
**genes used for computing fibrosis score**	**genes used for computing mechanotransduction score**
AGT	ABL1
AGTR1	ABL2
AKT1	ACTA1
ALOX12	ACTA2
BLMH	ACTB
BRCA1	ACTC1
BRCA2	ACTG1
CASP1	ACTG2
CASP3	ACTN1
CAV1	ACTN2
CCL2	ACTN3
CCL7	ACTN4
CD109	ACTR2
CD19	ACTR3
CD226	AJUBA
CD40	AKT1
CNR1	AKT2
COL1A1	AKT3
COL7A1	AMOT
CSF3	ANLN
CTGF	ARF1
CTNNB1	ARF3
CX3CL1	ARF4
CX3CR1	ARF5
CXXC5	ARF6
DGKA	ARHGAP1
DKK1	ARHGAP12
DPT	ARHGAP26
DSP	ARHGAP35
EN1	ARHGAP4
FAAH	ARHGAP5
FBN1	ARHGAP6
FBRS	ARHGAP9
FGF7	ARHGEF1
FLI1	ARHGEF11
FN1	ARHGEF12
FOSL2	ARHGEF7
FOXP3	ARPC1A
GATA1	ARPC1B
GNAS	ARPC2
GSK3B	ARPC3
HAX1	ARPC4
HIF1A	ARPC5

HPS1	ARPC5L
HPS4	ASAP1
HPS5	BAIAP2
HSP90AB1	BCAR1
ICAM1	BCAR3
IL1RN	BRAF
ITGAV	BTRC
ITGB1	CAPN1
ITGB3	CAPN10
JUN	CAPN2
KLF5	CAPN3
LAMP1	CAPN5
LEP	CAPN7
LOXL2	CAPN8
LSP1	CAPNS1
MFAP5	CAV1
MMP1	CD44
MMP14	CDC42
MMP2	CDC42EP1
MMP9	CDC42EP2
NOTCH1	CDC42EP3
NOX4	CDC42EP4
NR1H2	CDC42EP5
NR1H3	CFL1
NR4A1	CFL2
PANX1	CIT
PDGFRA	CRB1
PDGFRB	CRK
PDPK1	CRKL
PDZK1	CSNK1D
PLAUR	CSNK1E
PLOD2	CTTN
PNPLA2	CUL1
PPARD	DIRAS3
PPARG	DLC1
PTEN	DLG1
PTK2	DLG2
RBFOX2	DLG3
REST	DLG4
RLN1	DLG5
RRP9	DOCK1
S100A8	EPHA1
S100A9	ERAS
SCNN1A	EZR
SDC1	FAT4
SERPINF2	FNBP1
SFRP1	FRMD6
SIRT1	FYN
SMAD1	GIT1

SMAD2
SMAD3
SMAD7
SMO
SPARC
SPI1
SPP1
STAT3
STAT4
SYT7
TBX21
TGFB1
TIMP1
TNFSF14
TNPO3
TPH1
TRIB3
TSLP
VDR
VEGFA
VEGFB
WNK1

GNA12
GNA13
GRB2
GRB7
GSK3B
GSN
HRAS
IGF1R
ILK
ILKAP
ITCH
ITGA1
ITGA10
ITGA11
ITGA2
ITGA2B
ITGA3
ITGA4
ITGA5
ITGA6
ITGA7
ITGA8
ITGA9
ITGAE
ITGAL
ITGAV
ITGAX
ITGB1
ITGB2
ITGB3
ITGB4
ITGB5
ITGB7
ITGB8
KRAS
KTN1
LATS1
LATS2
LIMK1
LIMK2
LIMS1
LLGL1
LPAR1
LPAR2
LPAR3
LPAR4
LPAR6
MAP2K1
MAP2K2

MAP2K4
MAP3K11
MAPK1
MAPK3
MAPK8
MOB1A
MPRIP
MRAS
MSN
MST1
MYL12A
MYL12B
MYL3
MYL4
MYL5
MYL6
MYL6B
MYL7
MYL9
MYLK
MYLK2
MYLK3
MYLPF
NCK1
NCK2
NEDD4
NEDD9
NF2
NGEF
NRAS
NRP2
PAK1
PAK2
PAK3
PAK4
PAK6
PARD3
PARVA
PARVB
PATJ
PFN1
PFN2
PFN3
PFN4
PI4KA
PIEZO1
PIK3C2A
PIK3C2B
PIK3C3

PIK3CA
PIK3CB
PIK3CD
PIK3R1
PIK3R2
PIK3R3
PIK3R4
PIK3R6
PIKFYVE
PIP4K2A
PIP4K2B
PIP4K2C
PIP5K1A
PIP5K1B
PIP5K1C
PIP5KL1
PKN1
PLCG1
PLCG2
PLD1
PLEKHG5
PLXNA1
PPM1J
PPM1L
PPP1CA
PPP1CB
PPP1CC
PPP1R10
PPP1R11
PPP1R12A
PPP1R12B
PPP1R14A
PPP1R14B
PPP1R14C
PPP1R3C
PPP1R3D
PPP1R7
PPP2CA
PPP2CB
PPP2R1A
PPP2R1B
PPP2R2A
PPP2R2C
PPP2R3A
PPP2R3B
PPP2R5A
PPP2R5B
PPP2R5C
PPP2R5D

PPP2R5E
PTEN
PTK2
PTK2B
PTPA
PXN
RAC1
RAC2
RAC3
RAF1
RALA
RALB
RAP1A
RAP1B
RAP2A
RAP2B
RAPGEF1
RAPGEF2
RAPGEF6
RASD1
RASSF1
RASSF6
RDX
RHOA
RHOB
RHOBTB1
RHOBTB2
RHOC
RHOD
RHOF
RHOG
RHOH
RHOJ
RHOQ
RHOT1
RHOT2
RHOU
RHOV
RHPN1
RHPN2
RND1
RND2
RND3
ROCK1
ROCK2
RRAS
RRAS2
RTKN
SAV1

SCRIB
SEMA3F
SFN
SHC1
SKP1
SKP2
SMAD1
SMAD2
SMAD3
SMAD4
SMAD5
SOS1
SOS2
SRC
STK3
STK4
TEAD1
TEAD2
TEAD3
TEAD4
TJP2
TLN1
TLN2
TNK2
TP53BP2
TRPM7
TRPM8
TRPV4
TSPAN1
TSPAN2
TSPAN3
TSPAN4
TSPAN5
TSPAN6
TSPAN7
TTN
VASP
VCL
WAS
WASF1
WASL
WIPF1
WWC1
WWTR1
YAP1
YWHAB
YWHAE
YWHAG
YWHAH

YWHAQ
YWHAZ
ZYX
