# Supplementary material for: Identification of a physiologic vasculogenic fibroblast state to achieve tissue repair
Source: Nat Commun. 2023 Feb 28;14:1129. doi: 10.1038/s41467-023-36665-z (PMC9975176; doi:10.1038/s41467-023-36665-z)
Supplement: Supplementary file 2 — Description of Additional Supplementary Files [file 41467_2023_36665_MOESM2_ESM.pdf]

Description of Additional Supplementary Files:

Supplementary Movie 1: Video showing 3D rendering of the image shown in Figure 7h.
