# Supplementary material for: Identification of a physiologic vasculogenic fibroblast state to achieve tissue repair
Source: Nat Commun. 2023 Feb 28;14:1129. doi: 10.1038/s41467-023-36665-z (PMC9975176; doi:10.1038/s41467-023-36665-z)
Supplement: Supplementary file 4 — Reporting Summary [file 41467_2023_36665_MOESM4_ESM.pdf]

Corresponding author(s): Chandan K Sen

Last updated by author(s): Dec 29, 2022

Reporting Summary

Nature Portfolio wishes to improve the reproducibility of the work that we publish. This form provides structure for consistency and transparency in reporting. For further information on Nature Portfolio policies, see our Editorial Policies and the Editorial Policy Checklist.

Statistics

For all statistical analyses, confirm that the following items are present in the figure legend, table legend, main text, or Methods section.

n/a	Confirmed	
☐	☒	The exact sample size (*n*) for each experimental group/condition, given as a discrete number and unit of measurement
☐	☒	A statement on whether measurements were taken from distinct samples or whether the same sample was measured repeatedly
☐	☒	The statistical test(s) used AND whether they are one- or two-sided<br>*Only common tests should be described solely by name; describe more complex techniques in the Methods section.*
☐	☒	A description of all covariates tested
☐	☒	A description of any assumptions or corrections, such as tests of normality and adjustment for multiple comparisons
☐	☒	A full description of the statistical parameters including central tendency (e.g. means) or other basic estimates (e.g. regression coefficient) AND variation (e.g. standard deviation) or associated estimates of uncertainty (e.g. confidence intervals)
☐	☒	For null hypothesis testing, the test statistic (e.g. F, t, r) with confidence intervals, effect sizes, degrees of freedom and P value noted<br>*Give P values as exact values whenever suitable.*
☒	☐	For Bayesian analysis, information on the choice of priors and Markov chain Monte Carlo settings
☒	☐	For hierarchical and complex designs, identification of the appropriate level for tests and full reporting of outcomes
☒	☐	Estimates of effect sizes (e.g. Cohen's *d*, Pearson's *r*), indicating how they were calculated

Our web collection on statistics for biologists contains articles on many of the points above.

Software and code

Policy information about availability of computer code

Data collection	CellRanger v3.0.2 (http://support.10xgenomics.com/) was utilized to process the raw sequence data generated. Briefly, the FASTQ files were aligned to the human reference genome hg38 with RNAseq aligner STAR. The gene expression level of individual genes were quantified based on the number of UMIs (unique molecular identifiers) detected in each cell. The generated filtered gene-cell barcode metrices were used for further analysis.
Data analysis	Seurat package (v.3.1.1 and v4.0) in R (v.3.5.1) was used for preprocessing and visualization. The initial dataset contained 40,212 cells from 5 samples (HADF and 4 samples transfected with anti-miR-200b at day 1, 3, 5 and 7). Gene expression values were log normalized and scaled to 10,000 transcripts per cell. The top 2,000 variable genes were identified. Then, principal component analysis (PCA) was performed. For quality control, cells with more than 10% mitochondrial RNA, less than 200 or more than 8,000 detected transcripts and cells with less than 500 or more than 60,000 of total number of counts were excluded. Additionally, genes that were detected in fewer than 3 cells were excluded. After quality control, 36,308 cells were maintained for downstream analysis. PCA was performed again after filtration and the top 15 principal components were used for clustering the cells, resulting in 4 main clusters. Cytoscape (v3.7.1) was used to visualize biological processes along with the genes.<br><br>Monocle package in R was used to infer the trajectory and to order the cells across the pseudotime. It uses a reversed graph embedding algorithm to identify the sequence of change in gene expression each cell must go through across developmental processes.<br><br>To check the cycling difference between the vasculogenic cluster and parent cluster different clusters post miR-200b inhibition, cell cycle analyses were performed to identify the ratio of dividing cells and non-proliferating cells within clusters 0 and 1. Assigning cell cycle phase for each cell within clusters 0 and 1 was performed using the function CellCycleScoring in Seurat.

To investigate the role of VF in communication with the other cell types identified in DFU samples, CellChat was employed. The connectome thus established for VF with cell types was compared with non-VF cells directed connectome with other cell types. Top 10% differential connectome from the analyses further indicated strong interaction of VF with endothelial cells (cluster 3), smooth muscle cells 1-2 and basal cells/keratinocytes.

For perfusion analysis, color coded perfusion maps were acquired at all time points and average perfusion was calculated using PimSoft v1.4 software (Perimed Inc., Sweden).

Comparative analysis for identifying vasculogenic fibroblasts or endogenous vascular endothelium was performed using Axiovision Rel 4.8 software and Olympus FV1000 software. The number of all CD31+ elements or FSP-1+CD31+ elements was counted using the automatic particle counting tool feature of ImageJ after adoption of a fixed threshold for every image.

Densitometric quantification of Western blot data were performed using ImageJ (v1.53T, 24 Aug 2022) software.

Imaging Mass Cytometry. Data acquisition was performed on a Helios time-of-flight mass cytometer coupled to the Hyperion Imaging System (Fluidigm). Before laser ablation, optical images of slides were acquired using the Hyperion v1.0.560.6 software. Laser ablation was performed at a resolution of approximately 1 mm and a frequency of 200 Hz.

All codes used in the study are provided in the supplementary files.

For manuscripts utilizing custom algorithms or software that are central to the research but not yet described in published literature, software must be made available to editors and reviewers. We strongly encourage code deposition in a community repository (e.g. GitHub). See the Nature Portfolio guidelines for submitting code & software for further information.

Data

Policy information about availability of data

All manuscripts must include a data availability statement. This statement should provide the following information, where applicable:

- Accession codes, unique identifiers, or web links for publicly available datasets
- A description of any restrictions on data availability
- For clinical datasets or third party data, please ensure that the statement adheres to our policy

Source data are provided with this paper.
The raw single cell RNA sequencing analysis was deposited in the Gene Expression Omnibus (GEO) https://www.ncbi.nlm.nih.gov/geo/query/acc.cgi?acc=GSE167406
Processed single cell datasets were downloaded from Gene Expression Omnibus (GEO) with accession number GSE165816 (https://www.ncbi.nlm.nih.gov/geo/query/acc.cgi?acc=GSE165816)
The other data that support the findings of this study are available from the corresponding authors upon reasonable request.

Human research participants

Policy information about studies involving human research participants and Sex and Gender in Research.

Reporting on sex and gender	Finding apply to both sexes and not gender as reported in EMR.
Population characteristics	Human skin and wound biopsy samples were obtained from healthy adult human subjects or chronic wound patients, respectively, at Indiana University and The Ohio State University Comprehensive Wound Center (CWC). The covariate-relevant population characteristics of the human research participants are presented in Extended Data Figure 9b.
Recruitment	Wound biopsy samples were obtained from clinically diagnosed diabetic and non-diabetic patients at Indiana University or The Ohio State University. Participants were recruited using the IRB approved exclusion and inclusion criteria and do not had any self-selection bias or other biases.
Ethics oversight	All human tissue-based experiments were reviewed and approved by Institutional Review Board (IRB) of Indiana University or The Ohio State University. Declaration of Helsinki protocols was followed, and patients gave their written informed consent. Additionally, under IRB–approved protocols, surgically discarded and de-identified samples were collected. This included the collection of wound tissue obtained from individuals undergoing elective surgeries. In the cases of surgically discarded and de-identified samples, informed consent was not required from the subjects.

Note that full information on the approval of the study protocol must also be provided in the manuscript.

Field-specific reporting

Please select the one below that is the best fit for your research. If you are not sure, read the appropriate sections before making your selection.

☒ Life sciences ☐ Behavioural & social sciences ☐ Ecological, evolutionary & environmental sciences

For a reference copy of the document with all sections, see nature.com/documents/nr-reporting-summary-flat.pdf

Life sciences study design

All studies must disclose on these points even when the disclosure is negative.

Sample size	Immunohistochemistry, Immunocytochemistry, flow cytometry, RT-qPCR, Western blot, ultrasound based imaging, Laser speckle perfusion imaging, hind-limb ischemic study, miRNA target luciferase reporter assay were performed on multiple independent biological replicates (n shown in figure legends). No statistical methods were used to predetermine sample size. Patient number was selected to give balanced representation of diabetic and non diabetic wounds. For all animal studies, group sizes are reported in the main text, figure legend or within the figure. We used group sizes sufficient to detect statistically reliable effects.
Data exclusions	For single cell RNA sequencing quality control, cells with more than 10% mitochondrial RNA, less than 200 or more than 8,000 detected transcripts and cells with less than 500 or more than 60,000 of total number of counts were excluded. Additionally, genes that were detected in fewer than 3 cells were excluded. Data were excluded from self-administration experiments only if animals died or the skin hair growth started hindering the perfusion measurements during the experiment.
Replication	All experimental findings reported here were successfully replicated across multiple biological samples (n reported in each figure legend). All immunofluorescence was performed on a minimum 3 independent skin or wound-edge tissue samples.
Randomization	One group of randomly selected healthy skin samples and another set of randomly selected diabetic and non-diabetic wound-edge samples were analyzed in this study. For mouse experiments, age-matched litter-mate were randomly assigned using a computer based algorithm (www.random.org).
Blinding	Samples were coded and data analysis were performed by individual who was blinded to the origin of the samples. All analyses were performed in an automated manner across conditions.

Reporting for specific materials, systems and methods

We require information from authors about some types of materials, experimental systems and methods used in many studies. Here, indicate whether each material, system or method listed is relevant to your study. If you are not sure if a list item applies to your research, read the appropriate section before selecting a response.

Materials & experimental systems

n/a	Involved in the study	
☐	☒	Antibodies
☐	☒	Eukaryotic cell lines
☒	☐	Palaeontology and archaeology
☐	☒	Animals and other organisms
☒	☐	Clinical data
☒	☐	Dual use research of concern

Methods

n/a	Involved in the study	
☒	☐	ChIP-seq
☐	☒	Flow cytometry
☒	☐	MRI-based neuroimaging

Antibodies

Antibodies used

1. Anti-FLI-1; Abcam (Cat# ab15289); Dilution- 1:50
2. Anti-S100A4/Anti-FSP-1; Abcam (Cat# ab27957); Dilution-1:200
3. Anti-CD105; Abcam (Cat# ab107595); Dilution-1:500
4. Anti-CD31; BD Pharmingen (Cat# 550274); Dilution-1:200
5. Anti-Vwf; Abcam (Cat# ab6994); Dilution-1:200
6. APC anti-human CD144 (VECadherin) Antibody (Clone: BV9); Biolegend; (Cat# 348508); Dilution-5 µl per 10^6 cells
7. PE anti-human CD31; Biolegend; (Cat# 303106); Dilution- 5 µl per 10^6 cells
8. PE anti-human CD309 (VEGFR2) [Clone: 7D4-6]; Biolegend; (Cat# 359904); Dilution- 5 µl per 10^6 cells
9. FITC anti-human CD90 (Thy1) antibody (Clone: 5E10);Biolegend; (Cat# 328107); Dilution- 5 µl per 10^6 cells
10. Monoclonal Anti-β-Actin antibody (Clone: AC-15);Sigma; (Cat# A5441); Dilution-1:5000
11. F4/80 antibody (Clone: Cl:A3-1); Biorad; (Cat# MCA497); Dilution - 1:200
12. APC anti-human CD49B; Biolegend; (Cat#359310-100 test); Dilution - 1:200
13.PE anti-human β2-microglobulin Antibody; Biolegend; (Cat#316306); Dilution - 1:200
14. Goat anti-Rabbit IgG (H+L) Highly Cross-Adsorbed Secondary Antibody, Alexa Fluor™ Plus 488 ;Thermo Fisher Scientific; (Cat # A32731) Dilution 1:200
15. Goat anti-Rabbit IgG (H+L) Cross-Adsorbed Secondary Antibody, Alexa Fluor™ 568 hermo Fisher Scientific; (Cat # A11011) Dilution 1:200
16. Amersham ECL Rabbit IgG, HRP-linked whole Ab. CiteAb; (Cat NA934-1ML); Dilution 1:3000

Validation

All antibodies used are commercially available and validated by the vendor for the assay and species used in the study. Specific validation information for each antibody is available on the vendor's website. Additional validation for immunohistochemistry was performed using isotype controls. The specificity of flow cytometry antibody was validated by staining directly against species-matched isotype, no-primary controls or unstained controls.

Eukaryotic cell lines

Policy information about cell lines and Sex and Gender in Research

Cell line source(s)	1. Primary Dermal Fibroblast; Normal, Human, Adult (ATCC® PCS-201-012™); Bacteria and Yeasts: Negative. Mycoplasma: Negative<br>2. CDC-HMEC-1 cells (SV40 T antigen transformed human microvascular endothelial cells) were provided by the Center for Disease Control (CDC Atlanta, GA); Mycoplasma: Negative
Authentication	Primary Dermal Fibroblasts were tested for surface marker and were found to be CD90+CD31-/FSP-1+/VEGFR2-.<br>The HMEC cells were tested for surface marker and were found to be CD31+/VEGFR2+
Mycoplasma contamination	Primary human dermal fibroblast used were Mycoplasma negative as per the information available on the Vendor's website. The tdTomato-ECFCs cells were not tested for mycoplasma contamination.
Commonly misidentified lines (See ICLAC register)	None

Animals and other research organisms

Policy information about studies involving animals; ARRIVE guidelines recommended for reporting animal research, and Sex and Gender in Research

Laboratory animals	1. Male C57BL/6 mice (8-10 weeks old) were obtained from Harlan Laboratory.<br>2. Mice homozygous (BKS.Cg-m+/+Leprdb/J, or db/db; stock no 000642) for spontaneous mutation of the leptin receptor (Leprdb) or their respective non-diabetic lean control littermates m+/db (8-10 weeks old) were obtained from Jackson Laboratory, Bar Harbor, ME.<br>3. FSP1-Cre mouse was a kind gift from Dr. Arjun Deb (University of California, Los Angeles, California). Fsp1-Cre mice were crossed with the R26RtdTomato mice (JAX) carrying floxed tdTomato allele. Both male and female Fsp1-Cre:R26RtdTomato mice (8-12 weeks old) were used in the experiment.<br>4. Floxed miR-200b-429 mice (miR-200b-429fl/fl were generated by introducing loxP sites around miR-200b-429 gene in C57BL/6J mice. Fibroblast-specific miR-200b knockout mice were generated by breeding our miR-200b-429fl/fl with tamoxifen-inducible Col1a2-CreER mice (Stock: 029235, Jackson Laboratories) to obtain fibroblast specific inducible miR-200b knockout progeny [miR-200b-429fl/fl-Col1a2CreER].<br>5. For lineage tracing experiments, miR-200b-429fl/fl-Col1a2CreER mice were crossed with ROSAmT/mG mice (Stock: 007576, Jackson Laboratories).<br>6. Male NSG mice (NOD.Cg-Prkdcscid Il2rgtm1Wjl/SzJ ; Strain #:005557) of 12 weeks were obtained from Jackson Laboratories.<br>The animals were housed in IU laboratory animal facility with 12h light and dark cycles with access to food and water ad libitum.
Wild animals	Study did not involve wild animals
Reporting on sex	Both sexes were used
Field-collected samples	Study did not involve samples collected in the field.
Ethics oversight	All animal studies were performed in accordance with protocols approved by the Laboratory Animal Care and Use Committee of Indiana University and The Ohio State University.

Note that full information on the approval of the study protocol must also be provided in the manuscript.

Flow Cytometry

Plots

Confirm that:

☒ The axis labels state the marker and fluorochrome used (e.g. CD4-FITC).

☒ The axis scales are clearly visible. Include numbers along axes only for bottom left plot of group (a 'group' is an analysis of identical markers).

☒ All plots are contour plots with outliers or pseudocolor plots.

☒ A numerical value for number of cells or percentage (with statistics) is provided.

Methodology

Sample preparation	Please see Method for detailed sample preparation protocol for FACS and flow cytometry
Instrument	BD Acuri, BD LSR II and Aria III flow cytometers were used for flow cytometry and cell sorting.
Software	BD FACS Diva software v.8 was used for cell sorting. Data was analyzed with FlowJo software (version 10.0.7).

Cell population abundance	Sort purity was routinely over 95% on post-sort checks
Gating strategy	Initial gating for all experiments: Cells (FSC-A vs SSC-A or FSC-A vs FSC-H). Gates were defined by comparison with control inhibitor transfected samples.

☒ Tick this box to confirm that a figure exemplifying the gating strategy is provided in the Supplementary Information.
